# Pd-Catalyzed Ring-Opening Polymerization of Cyclobutanols through C(sp^3^)–C(sp^3^) Bond Cleavage

**DOI:** 10.1021/acs.macromol.4c01089

**Published:** 2024-07-09

**Authors:** Sergio Parra-García, Isabel Saura-Llamas, Delia Bautista, Juan Gil-Rubio, José-Antonio García-López

**Affiliations:** † Departamento de Química Inorgánica, Universidad de Murcia, Campus de Espinardo, 30100 Murcia, Spain; ‡ ACTI, Universidad de Murcia, Campus de Espinardo, 30100 Murcia, Spain

## Abstract

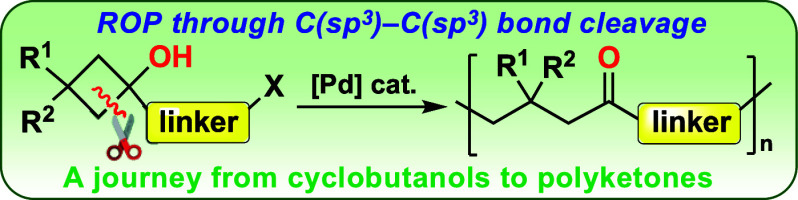

A new approach to ring-opening polymerization (ROP) based on C(sp^3^)–C(sp^3^) bond cleavage is reported. This process is based on the ability of Pd to promote both the β-carbon elimination of a bifunctional cyclobutanol precursor and the C–C coupling process with the resulting Pd-alkyl intermediate. Consequently, novel polyketone materials are obtained. Owing to the modular synthesis of the used cyclobutanol monomers, the present ROP reaction allows the introduction of substitution patterns in the polymeric chain that are not accessible by current polyketone synthesis methodologies. We have explored in detail the initiation, propagation, and termination steps of this new polymerization process.

## Introduction

**Scheme 1:**
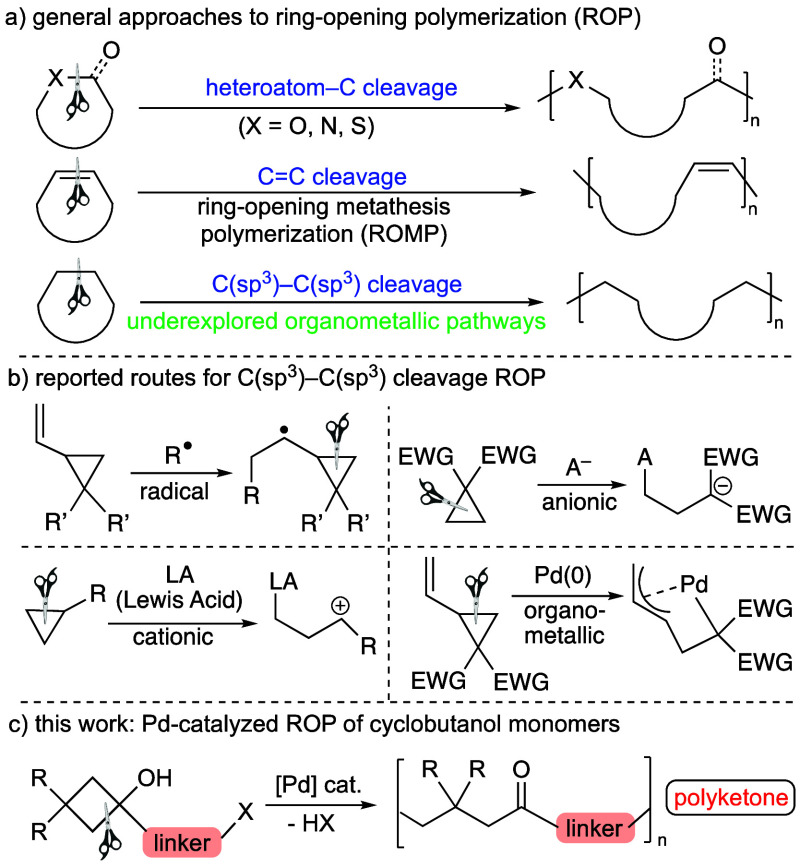
Previously Described (a, b) and New (c) Approaches to ROP

The polymerization processes based on the opening of cyclic monomers (ring-opening polymerization, ROP) have been deeply studied during the last years^1−4^ and keep on being at the forefront of the materials science research arena.^5−7^ Two different types of ROP methodologies can be distinguished: (a) those based on the cleavage of C–heteroatom bonds and (b) those in which C–C or C=C bonds of cyclic monomers are split. In the first case, these processes commonly rely on the use of heterocyclic rings, such as lactides, carbonates, or epoxides, in which a C–O bond is broken (Scheme 1a).^2,4,8^ Within the second group, the most studied processes deal with the use of cyclic alkenes, such as norbornene, in which the C=C bond undergoes a metathesis reaction (known as ROMP) (Scheme 1a).^9,10^ The possibility to harness the cleavage of single carbon–carbon bonds of molecular skeletons offers huge opportunities to develop new synthetic routes, given the ubiquitous presence of such chemical linkages in organic compounds.^11−14^ However, the availability of ROP methods that make use of the cleavage of C(sp^3^)–C(sp^3^) bonds is much more restricted compared to those based on the C–heteroatom or C=C bond cleavage.^15^ These methodologies are based on the use of strained carbocycles, which can be polymerized through radical, anionic, or cationic mechanisms (Scheme 1b). For instance, vinyl-substituted cyclopropanes have proven to be useful substrates for radical-initiated ROP.^16^ The cyclopropyl rings bearing electron-withdrawing groups are, however, especially suited for anionic ROP.^17^ Furthermore, the use of adequate Lewis acids can lead to the cationic polymerization of electron-rich cyclopropyl monomers.^18^ In contrast, the polymerization of strained carbocycles involving organometallic intermediates formed upon C(sp^3^)–C(sp^3^) bond cleavage has rarely been reported. A remarkable example of this approach was reported by Saegusa and co-workers, who described the use of 2-vinylcyclopropane-1,1-dicarboxylate as a suitable monomer for Pd-catalyzed ROP, relying on the oxidative addition of the cyclopropyl moiety to Pd(0) and the subsequent formation of a π-allyl intermediate (Scheme 1b).^19^

We aim to extend the applicability of TM-catalyzed C–C bond cleavage to the discovery of complementary ROP processes occurring via organometallic intermediates, thus allowing the preparation of polymeric materials with new substitution patterns difficult to access through other routes.

Strained cycloalkanols have been successfully incorporated into synthetic routes involving C–C cleavage, given their ability to coordinate to transition metals and generate σ-alkyl organometallic intermediates upon a β-C elimination step. These last species can undergo further coupling processes depending on the reaction conditions.^20−26^ While they have been widely used for small molecule functionalization, there is only one reported application for the synthesis of polymeric materials. It relates the obtention of a polythiophene chain from a thiophene derivative of 9-fluorenol.^27^ We envisioned that the use of a bifunctional cyclobutanol monomer bearing a suitable leaving group tethered to the cycloalkanol moiety through a linker could lead to the formation of a polyketone (Scheme 1c). Aliphatic^28^ or aromatic^29,30^ polyketones have received a longstanding interest because of their useful properties and photodegradability.^31^ In contrast, hybrid polyketones containing both aliphatic and aromatic units in the main chain are almost unexplored.^32,33^

## Results and Discussion

We began our study by synthesizing the cyclobutanol monomer **1a**, containing a phenylene group as the linker between the two complementary reactive sites, which could be obtained on gram scale by reacting 4-bromophenylmagnesium bromide and 3-methyl-3-phenylcyclobutanone. In order to assess the feasibility of the polymerization process, we set an initial experiment heating the monomer **1a** in toluene at 100 °C in the presence of a 10 mol % of [Pd(PPh_3_)_4_] and Cs_2_CO_3_ (1.1 equiv). To our delight, a 34 % yield of polyketone **P1** could be isolated from the reaction mixture. Next, we carried out a systematic study of different conditions (Pd source, ligands, solvent, base) to outline the main characteristics of the polymerization process (Table 1, see full optimization study in the SI file). The combination of Pd(OAc)_2_/PPh_3_ seemed to work better than [Pd(PPh_3_)_4_], while [Pd(dba)_2_]/PPh_3_ afforded just a minor amount of polymer. Among the ligands that were screened, there were no significant differences in the yields and average molecular weights of the polyketones obtained when using PPh_3_, DPPF, DPPE, or DPE as ligands. Among the aliphatic phosphines tested, PCy_3_ and P^*n*^Bu_3_ were productive in the reaction albeit did not outperform PPh_3_. Other ligands such as 2,2′-bipyridine or the NHC precursor 1,3-bis(2,6-diisopropylphenyl)imidazolium chloride failed to provide the desired polyketone. In terms of solvent, toluene was a slightly better option than 1,4-dioxane, 1,2-dichloroethane, THF, chlorobenzene, or *t*-amyl-alcohol, with the reaction being shut down in DMF, NMP, or MeCN. The polymerization progressed similarly with Cs_2_CO_3_ or CsF as the base, while K_2_CO_3_ gave polyketones with slightly lower *M*_w_, and Et_3_N blocked the process. A dependence of the polymer average molecular weight with the concentration of the catalyst was observed. Thus, the lowering of the Pd catalyst loading from 2 to 0.5 mol % afforded higher molecular weights albeit with an increase of the polydispersity of the material. Further reduction of the Pd loading to 0.25 or 0.125 mol % led to good yields of polymer but with a decrease in *M*_w_ values.

**Table 1:**
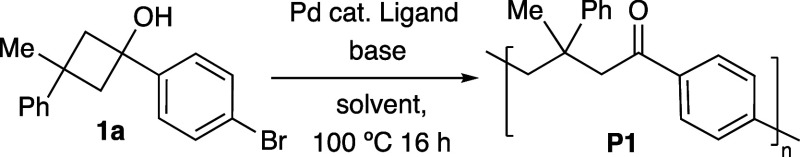
Selected Conditions for Pd-Catalyzed ROP Polymerization^a^

Pd (mol %)	ligand (mol %)	base (equiv)	yield^b^ (%)	*M*_w_ (kDa)/ *M*_n_ (kDa)	DP^c^	Đ
Pd(OAc)_2_ (2)	PPh_3_ (5)	Cs_2_CO_3_ (1.1)	88	6.3/3.6	15	1.75
[Pd(PPh_3_)_4_] (1)		Cs_2_CO_3_ (1.1)	64	5.5/3.1	13	1.75
Pd(OAc)_2_ (2)	DPPF (2)	Cs_2_CO_3_ (1.1)	89	5.7/2.9	12	1.96
Pd(OAc)_2_ (2)	PCy_3_ (5)	Cs_2_CO_3_ (1.1)	71	4.6/2.8	12	1.61
Pd(OAc)_2_ (0.5)	PPh_3_ (1)	CsF (1.5)	71	7.5/4.2	18	1.75
Pd(OAc)_2_ (1)	PPh_3_ (2)	Cs_2_CO_3_ (1.1)	78	10.9/5.7	24	1.92
Pd(OAc)_2_ (0.5)	PPh_3_ (1)	Cs_2_CO_3_ (1.1)	85	17.5/6.7	28	2.59
Pd(OAc)_2_ (0.125)	PPh_3_ (0.25)	Cs_2_CO_3_ (1.1)	97	9.4/3.9	17	2.38

a Reactions carried out using 0.5 mmol of **1a**, in 3 mL of dry toluene under the N_2_ atmosphere, heating to 100 °C for 16 h.

b Isolated yields.

c Mean degree of polymerization estimated with the *M*_n_ values and a (C_17_H_16_O)_*n*_ composition.

**Scheme 2:**
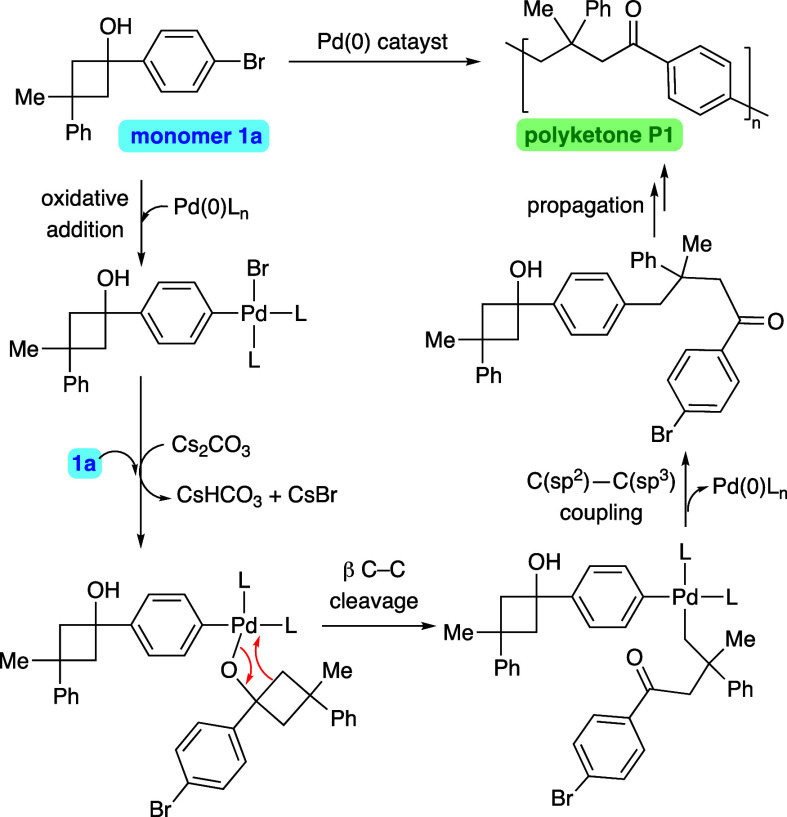
Proposed Reaction Pathway for the Polymerization Process

The polymerization process would be triggered by the oxidative addition of the C–Br bond present in the monomer to Pd(0). The first organometallic intermediate generated in this way could coordinate a second molecule of deprotonated cyclobutanol **1a** (Scheme 2). Subsequently, the above-described β-carbon elimination process would lead to a σ-alkyl Pd(II) intermediate. Next, reductive elimination with concomitant C–C bond formation would afford an enlarged bifunctional cyclobutanol, which in turn could restart the cycle.

This Pd-catalyzed ROP reaction provides a completely new approach to functionalized polyketones, materials that are mainly produced through olefin/CO copolymerization.^30,34−38^ In addition, our strategy allows the introduction of structural modifications in the chains using cyclobutanols with different substitution patterns or bearing different linker groups between the two reactive sites of the monomer. Thus, polymer **P2** containing two phenyl substituents was obtained from cyclobutanol **2** (Table 2). Nevertheless, a lower molecular weight compared to **P1** was observed, which may be due to the more probable occurrence of chain-end processes associated with higher number of aromatic rings in the structure (see below). The linking aryl group was modified in monomers **3**, **4**, and **5**, which gave rise to polymers **P3**, **P4**, or **P5**, containing fluorenylene, biphenylene, or 1,3-phenylene linkers, respectively. Finally, the palladium-catalyzed ROP reaction was also applied to fluorenol **6**, containing a less strained five-membered ring, although a low molecular weight polymer **P6** was obtained in 41 % yield, likely due to the enhanced difficulty of the ring-opening process in a five-membered ring compared to the four-membered one of the rest of monomers.

**Table 2:**
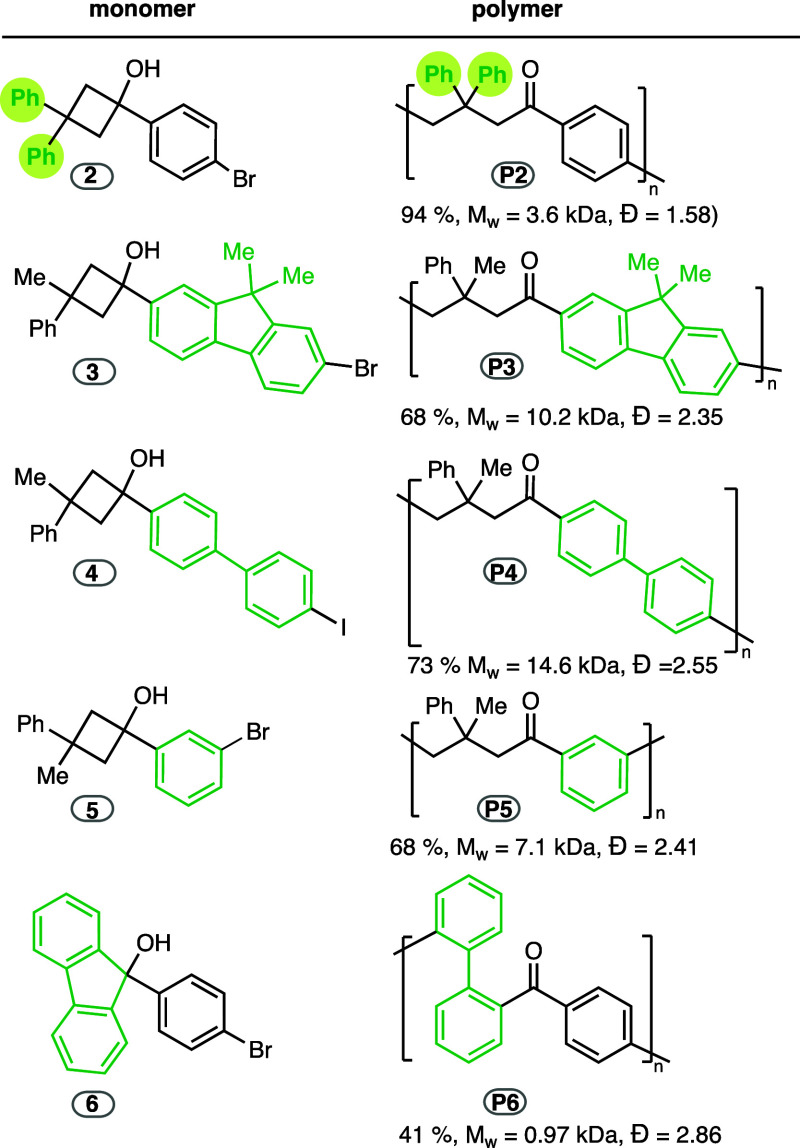
Scope of Polyketones with Different Substitution Patterns

TGA analyses showed that the decomposition temperatures of polymers **P2**, **P3**, **P4**, and **P5** were in the range of 338–387 °C, whereas that of **P6** was 280 °C (temperatures corresponding to a 5 % weight loss). Glass transition temperatures between 122 and 168 °C were determined by DSC for **P1**–**4**, respectively, while **P5** and **P6** did not show a clear glass transition (see SI).

**Figure 1:**
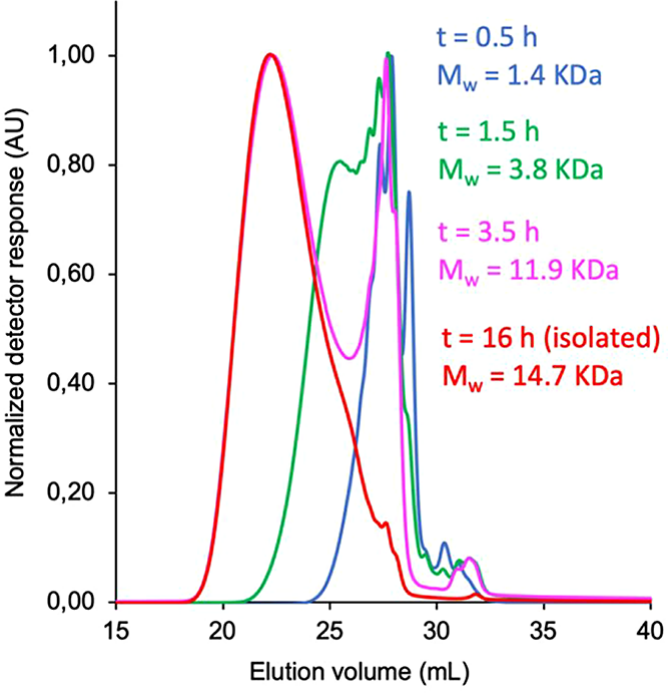
GPC traces of the polymerization reaction mixture of cyclobutanol **1a** at different reaction times and the isolated polymer.

The Pd-catalyzed formation of polyketones through β-carbon elimination seems to proceed in a stepwise manner. First, the monomers react to render oligomers that react with each other to produce chains with a higher molecular weight. This behavior was observed by monitoring the polymerization of **1a** in the presence of 1 mol % of Pd(OAc)_2_ and 2 mol % of PPh_3_. The aliquots taken from the mixture at 0.5 h (*M*_w_ = 1.4 kDa) and 1.5 h (*M*_w_ = 3.8 kDa) reaction times still showed the presence of unreacted **1a** (TLC) in the mixture. The GPC traces of such samples displayed several peaks of low molecular weights (Figure 1). With the increase of time (3.5 h), no monomer was detected by TLC and two main distributions appeared in the chromatogram, with a *M*_w_ value of 11.9 kDa. Upon 8 h, the isolated polymer displayed a *M*_w_ value of 14.7 kDa.

**Scheme 3:**
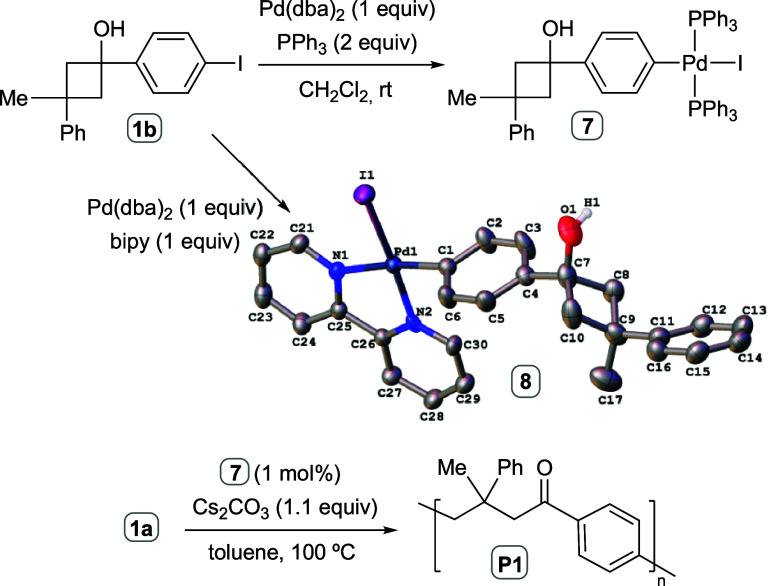
Stoichiometric Model Reactions of the Polymerization Initiation Steps Crystal structure of [Pd(C_17_H_17_O)I(bipy)] (**8**) is displayed.

In order to test our proposed mechanism, we carried out the stoichiometric reaction of iodinated monomer **1b** and Pd(dba)_2_ in the presence of PPh_3_ (2 equiv). The palladated cyclobutanol **7** could be isolated in 41 % yield from the reaction mixture (Scheme 3). Although it was not possible to obtain suitable crystals of **7** for X-ray diffraction studies, an analogue derivative **8** arising from the use of 2,2′-bipyridine instead of PPh_3_ as ligand could be properly crystallized (Scheme 3). When a 1 mol % of the complex **7** was used as a catalyst in the polymerization reaction, the expected polyketone **P1** was obtained in 63 % yield (*M*_w_ = 10.3 kDa), demonstrating its competence to promote the reaction. When the complex **8** was employed as a catalyst no polymerization was observed, recovering the cyclobutanol monomer **1a** unreacted. This behavior is not surprising since during the optimization study we performed an experiment using Pd(dba)_2_ as catalyst precursor and bipy as ligand, finding that no polymerization reaction took place (see SI).

**Figure 2:**
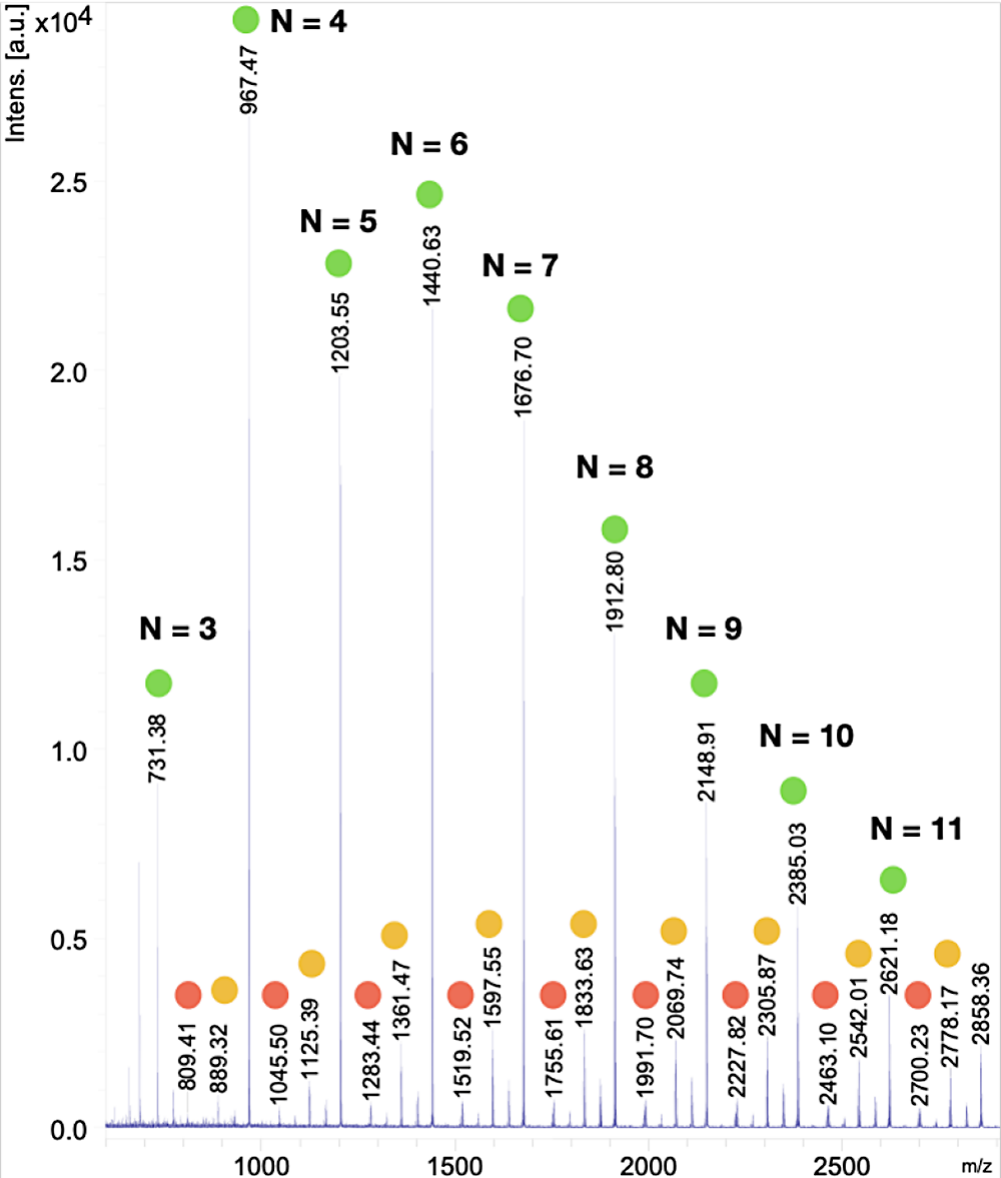
Region of MALDI spectrum from a sample of polyketone **P1** (Table 1, last entry). Color code: main polymer series [(C_17_H_16_O)_*n*_] + Na^+^ (green circles), selected secondary series [(C_17_H_16_O)_*n*_ + Ph + H] + Na^+^ (red circles), and [(C_17_H_16_O)_*n*_ + Br + Ph] + Na^+^ (yellow circles). The masses of the observed oligomers are smaller than the average MW determined by GPC (*M*_n_ = 3.9 kDa, *M*_w_ = 9.4 kDa). This is attributed to the mass discrimination of MALDI against high-mass oligomers in polydisperse polymers.^43,44^

**Scheme 4:**
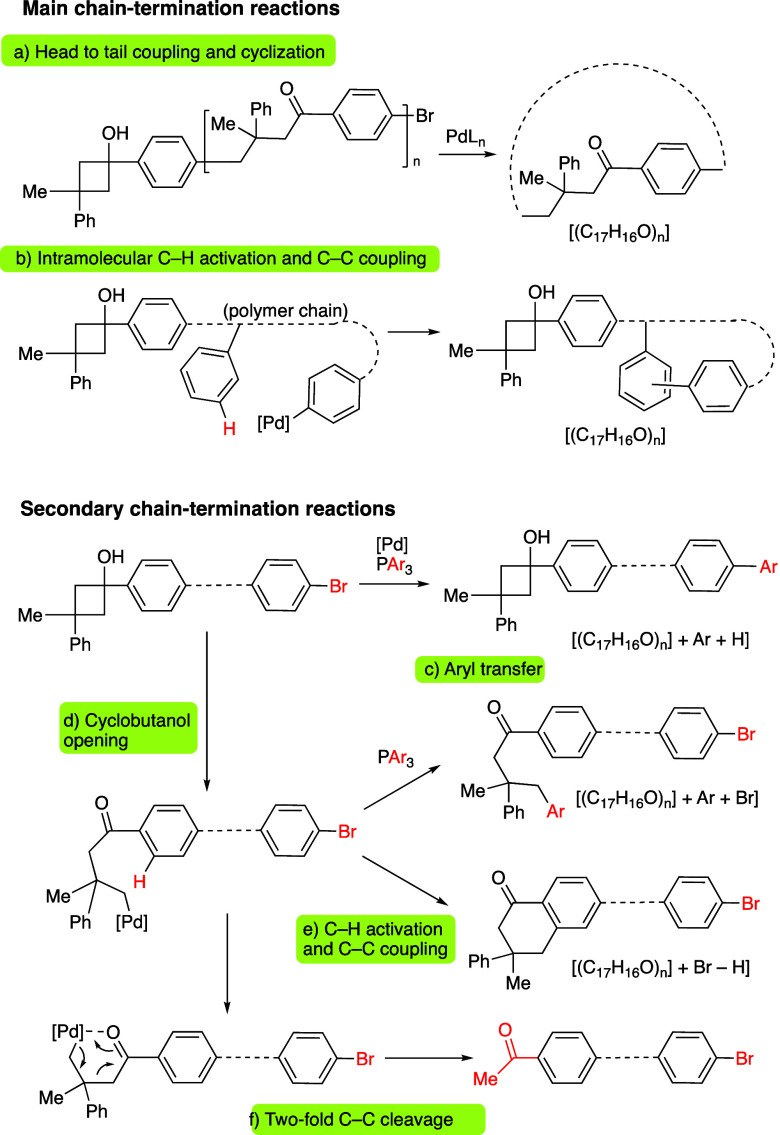
Proposed Chain-Termination Pathways According to MALDI MS Analyses

The analysis of the polymeric chain ends was assessed by measuring the MALDI-TOF mass spectra of the obtained polymers. In the case of **P1**, the spectrum showed the existence of a main series of polymeric chains with the formula [(C_17_H_16_O)_*n*_] (Figure 2), corroborating the incorporation of monomer units upon the formal loss of HBr from **1a** (molecular formula: C_17_H_17_BrO). These data suggest the existence of cyclic structures formed by a head to tail cyclization process. However, although the presence of cyclic oligomers cannot be discarded, the relatively high monomer concentration used (0.17 M) would statistically favor the intermolecular reaction between the active ends of two different chains versus the intramolecular head to tail cyclization (Scheme 4a), particularly for long chains. In addition, the NMR signals of the polymers appear typically broadened due to the slight differences in the chemical environments of the monomers as they are placed closer to the end groups of the polymeric chain. Then, the detected molecular formula [(C_17_H_16_O)_*n*_] might be explained through a C–H activation-mediated cyclization. For instance, the C_6_H_4_Br chain-end could undergo oxidative addition to Pd(0) rendering a palladated chain-end which, instead of incorporating a new monomer unit, could promote an intramolecular C–H activation in any of the nearby aryl groups of the polymer (Scheme 4b). A subsequent C–C coupling with reductive elimination would afford a cyclized structure with the same molecular mass as a head to tail cyclic polymer.

Some secondary series of the MALDI mass spectra showed the incorporation of a Ph group at one chain end (Scheme 4c). Considering that arylphosphines are able to transfer aryl groups to Pd,^39−41^ we carried out a polymerization reaction using P(*p*-OMe-C_6_H_4_)_3_ as ligand instead of PPh_3_. The MALDI mass and ^1^H NMR spectra of the obtained polymer showed the presence of a minor polymeric series containing a MeO-C_6_H_4_ group (see SI), thus confirming the aryl transfer from P to Pd under the reaction conditions.

Interestingly, a minor polymeric series showed a mass corresponding to a Me(C=O)-C_6_H_4_ end-group (Scheme 4f). We have previously observed that cyclobutanol reagents can undergo a Pd-catalyzed 2-fold C(sp^3^)–C(sp^3^) bond cleavage, especially when using JohnPhos as a ligand.^42^ This process provokes the formal [2 + 2]-retrocyclization of the cyclobutanol fragment, giving the corresponding alkene and acetophenone. In our case, we checked that the use of JohnPhos fully avoids polymerization of monomer **1a** in favor of the [2 + 2]-retrocyclization process. Under the standard polymerization conditions, using PPh_3_ as a ligand, this path occurs in a small proportion. The ^1^H NMR signal of the acetyl group arising from this termination pathway can be observed as a small singlet at 2.6 ppm.

The MALDI mass spectrum of **P1** also showed other secondary series in much lower proportion, each of them linked to the different types of quenching reactions that can happen in both the cyclobutanol and the C–Br ends of the chain (Scheme 4b–e; see also the Supporting Information for more details).

To gain a deeper insight on the polymerization termination process, we performed the polymerization of monomer **1a** with an increasing Pd loading. A decrease in the average *M*_w_ of the polymer was observed for higher catalyst loading, ranging from 6.3 kDa for a 2 mol % of Pd to 0.74 kDa for a 25 mol % of Pd. This fact may indicate the existence of further quenching processes such as transmetalation between two palladated chains and C–C coupling. Accordingly, the MALDI MS showed a pronounced decrease of the relative abundance of the [(C_17_H_16_O)_*n*_] series and a subsequent increase of other polymeric series with different end groups. Furthermore, when a polymerization reaction of monomer **1a** using a 2 mol % of Pd(OAc)_2_ was carried out under dilute conditions ([**1a**] = 0.025 M), low molecular weight oligomers were formed (*M*_w_ = 0.63 kDa) compared to the polymer obtained in concentrated conditions for the same Pd catalyst loading (*M*_w_ = 6.3 kDa). These data suggest the promotion of intramolecular quenching processes (such as C–H activation or head to tail cyclization) versus the intermolecular coupling, since the MALDI mass spectrum shows mainly the polymeric [(C_17_H_16_O)_*n*_] series, without a relevant presence of brominated chain-ends indicative of an incomplete reaction.

In summary, we have explored a new approach to the ROP field through C(sp^3^)–C(sp^3^) bond cleavage, a clearly underdeveloped area compared to ROP processes relying on C–heteroatom or C=C bond cleavage. This strategy takes advantage of conveniently designed bifunctional cyclobutanol monomers, which can undergo a Pd-catalyzed β-carbon elimination step, a route that we are currently exploring to outline further applications.

## Data Availability

CCDC 2350833 contains the supplementary crystallographic data for this paper. These data can be obtained free of charge via www.ccdc.cam.ac.uk/data_request/cif, or by emailing data_request@ccdc.cam.ac.uk, or by contacting The Cambridge Crystallographic Data Centre, 12 Union Road, Cambridge CB2 1EZ, UK; fax: + 44 1223 336033.

## Supporting Information Available

General remarks; synthesis and characterization of the cyclobutanol starting materials; synthesis and characterization of the polymers; mechanistic studies; GPC traces of the polymers; NMR spectra of the non-previously reported compounds; TGA and DSC traces of the polymers; and references (PDF)
