# Supplementary material for: Pd-Catalyzed Ring-Opening Polymerization of Cyclobutanols through C(sp3)–C(sp3) Bond Cleavage
Source: Macromolecules. 2024 Jul 9;57(14):6577–82. doi: 10.1021/acs.macromol.4c01089 (PMC11271690; doi:10.1021/acs.macromol.4c01089)
Supplement: Supplementary file 1 — ma4c01089_si_001.pdf [file ma4c01089_si_001.pdf]

Supporting Information

Pd-catalyzed ring-opening polymerization of cyclobutanols through C(sp^3)-C(sp^3) bond cleavage

Sergio Parra-García,[1] Isabel Saura-Llamas,[1] Delia Bautista,[2] Juan Gil-Rubio,[*,1] and José-Antonio García-López[*,1]

[1]Grupo de Química Organometálica, Departamento de Química Inorgánica, Facultad de Química, Universidad de Murcia, E-30100 Murcia, Spain. [2]ACTI, Universidad de Murcia, E-30100 Murcia, Spain.

E-mail: jgr@um.es, joangalo@um.es

TABLE OF CONTENTS

1. General remarks S3

2. Synthesis and characterization of the cyclobutanol starting materials S4

2.1. Chart of starting materials S4

2.2. Representative cyclobutanol synthesis S4

2.3. Characterization data of the cyclobutanol starting materials S5

3. Synthesis and characterization of the polymers S7

3.1. Representative polymerization procedure S7

3.2. Optimization of the polymerization reaction S7

3.3. Scope of the polymerization reaction S10

3.4. Characterization of the polymers S10

3.4.1. Spectroscopic and analytic data S10

3.4.2. MALDI-TOF MS studies of the polymer end groups and chain termination mechanisms S12

4. Mechanistic studies S24

4.1. Monitoring of the polymerization reaction S24

4.2. Synthesis and characterization of intermediate complexes S25

4.3. Crystal structure of complex **8** S27

4.4. Polymerization reaction employing the intermediate complex **7** as initiator S28

5. GPC traces of the polymers S29

6. NMR spectra of the non-previously reported compounds S30

7. TGA and DSC traces of the polymers S44

8. References S46

1. General remarks

Infrared spectra were recorded on a Jasco FT/IR-4600 spectrophotometer employing the ATR technique. High-resolution ESI mass spectra were recorded on an Agilent 6220 Accurate Mass TOF LC/MS spectrometer. GC/MS spectra were recorded on an Agilent GC/MS/QTOF 7250B spectrometer. Elemental analyses were performed on a LECO CNHS-932 instrument. TGA analyses were performed on a TA Instruments SDT 2960 thermal analyzer. DSC analyses were carried out on a TA Instruments DSC 2920 differential scanning calorimeter. The samples were heated under nitrogen at 10 ºC/min. The given decomposition temperatures for the polymers correspond to a 5 % mass loss. GPC measurements were carried out on a Waters Breeze chromatograph equipped with a Waters 248 UV-Visible detector operating at 254 nm and three Styragel columns (HR3, HR4E, and HR4) stabilized at 32 °C. Measurements were carried out in degassed, HPLC-grade $CHCl_3$, at 1 mL/min flow rate. The system was calibrated with eleven low-polydispersity polystyrene standards. Nuclear Magnetic Resonance (NMR) spectra were recorded on 300 or 400 MHz Bruker NMR spectrometers in $CDCl_3$ or DMSO-d_6 at 298 K. ^{1}H spectra were referenced to TMS, except those measured in DMSO-d_6, which were referenced to the residual protonated solvent signal. ^{13}C{^{1}H}-NMR spectra were referenced to solvent signal. ^{31}P{^{1}H}-NMR spectra were referenced to external H_3PO_4. Abbreviations used: br (broad), vd (virtual doublet). MALDI-TOF mass spectra were obtained in a Bruker Ultraflex MALDI TOF/TOF instrument in the positive reflection mode. DCTB doped with NaI was used as the matrix. In these conditions, (M + Na^+) molecular ions were observed. Toluene and THF were dried with a Pure Solv MD-5 solvent purification system from Innovative Technology and stored under N_2 over 4 Å molecular sieves. Other chemicals and solvents were used as received. TLC tests were run on TLC Alugram® Sil G plates and visualized under UV light at 254 nm. Preparative chromatographic separations were carried out in a Teledyne ISCO CombiFlash Netxgen chromatograph equipped with an UV detector and a silica gel column. A mixture of *n*-hexane and ethyl acetate was used as eluent. The ethyl acetate proportion usually was increased from 0% to 40%.

2. Synthesis and characterization of the cyclobutanol starting materials

2.1. Chart of starting materials

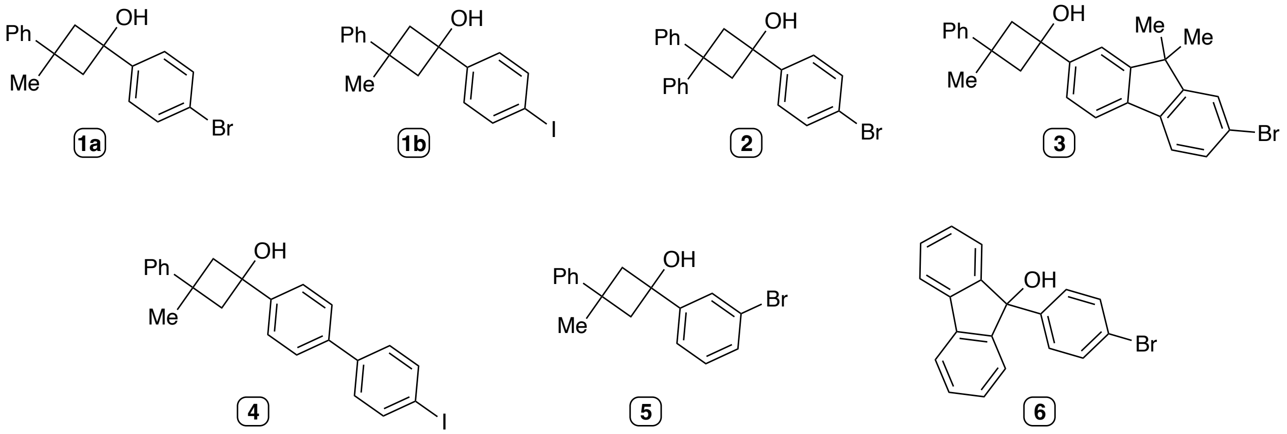

2.2. Representative cyclobutanol synthesis

All used cyclobutanol derivatives were prepared by a modification of a previously reported procedure. [1]

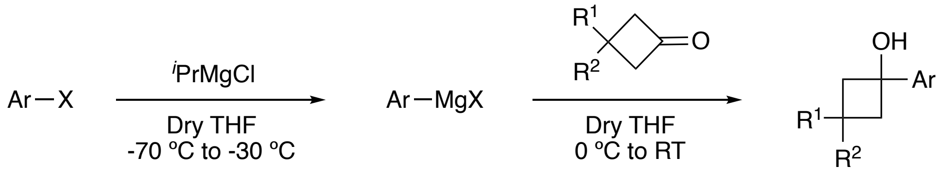

A 2 M solution of isopropylmagnesium chloride in THF was added dropwise under N_2 atmosphere to a solution of the corresponding aryl halide in dry THF at −70 °C. After 20 minutes of stirring, the bath temperature was increased to −30 °C, and the mixture was further stirred for 20 minutes. The resulting solution was added dropwise to a solution of the corresponding ketone in dry THF at 0 °C under N_2 atmosphere. The reaction was warmed to RT with continuous stirring (see the characterization data below to check the particular reaction times and quantities). The reaction was quenched with water (0.5 mL), the mixture was filtered and the solid residue was washed with Et_2O (2 × 5 mL). The filtrate was concentrated to ca. 2 mL, diluted with Et_2O (50 mL), and washed with water (30 mL). The aqueous layer was extracted with Et_2O (2 × 20 mL), and the combined organic layers were washed with brine, dried over $MgSO_4$, filtered, and concentrated in vacuum. The product was precipitated with *n*-pentane, filtered, and dried under reduced pressure or purified by flash column chromatography.

2.3. Characterization data of the cyclobutanol starting materials

The NMR data of **1a**, **1b** and **6** are in agreement with previously reported data.[2, 3]

1-(4-bromophenyl)-3,3-diphenylcyclobutan-1-ol (2). It was obtained from 1-bromo-4-iodobenzene (1275 mg, 4.50 mmol, 1.00 equiv.), isopropylmagnesium chloride (4.60 mmol, 1.05 equiv.) and 3,3-diphenylcyclobutan-1-one (1000 mg, 4.50 mmol, 1.00 equiv.) by following the representative procedure. After 3 h of reaction time, the product was isolated pure as a white solid by precipitation (436 mg, 1.15 mmol, 26%). IR (cm^{-1}): ν 3375 (ν(OH)), 2981, 2945, 1484, 1443, 1372, 1088, 1072, 1001, 967, 911, 818, 746, 694, 586, 559, 503. ^{1}H-NMR (300.1 MHz, $CDCl_3$): δ 7.46−7.06 (m, 14 H, Ar), 3.41 (vd, *J* = 13.3 Hz, 2 H, CH_2), 3.30 (vd, *J* = 13.4 Hz, 2 H, CH_2), 2.05 (s, 1 H, OH). ^{13}C{^{1}H}-NMR (75.5 MHz, $CDCl_3$): δ 149.2, 148.9, 145.0 (Ar C_q), 131.3, 128.6, 128.4, 126.9, 126.3, 126.0, 125.8, 125.7, 121.1 (Ar CH), 73.0 (C–O), 50.0 (*C*Ph_2), 43.8 (CH_2). HR-MS (+ESI) *m/z* calculated for $C_{22}H_{18}Br$ $[M-OH]^+$ 361.0586, found 361.0587.

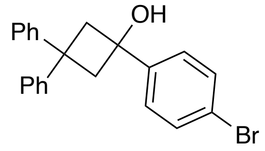

1-(7-bromo-9,9-dimethyl-9H-fluoren-2-yl)-3-methyl-3-phenylcyclobutan-1-ol (3). It was obtained from 2-bromo-7-iodo-9,9-dimethyl-9H-fluorene (1565 mg, 3.92 mmol, 1.14 equiv.), isopropylmagnesium chloride (2.1 mL, 4.10 mmol, 1.20 equiv.) and 3-methyl-3-phenylcyclobutan-1-one (550 mg, 3.43 mmol, 1.00 equiv.), by following the representative procedure. After 3 hours of reaction time, the product was isolated pure by flash column chromatography with *n*-hexane:EtOAc as eluents (814 mg, 1.88 mmol, 55%). The isolated white solid was a mixture of diastereoisomers in approx. 1:0.9 ratio. IR (cm^{-1}): ν 3319 (ν(OH)), 2960, 2928, 1452, 1256, 1187, 1028, 812, 759, 702, 543, 448. ^{1}H-NMR (400.9 MHz, $CDCl_3$): δ 7.71–7.14 (several m, 11 H, Ar), 3.06 (vd, *J* = 13.7 Hz, 2 H, CH_2, main isomer), 3.01 (vd, *J* = 12.7 Hz, 2 H, CH_2, minor isomer), 2.95 (vd, *J* = 12.7 Hz, 2 H, CH_2, minor isomer), 2.68 (vd, *J* = 13.5, 2 H, CH_2, main isomer), 2.03 (s, 1 H, OH, main isomer), 1.91 (s, 1 H, OH minor isomer), 1.73 (s, 3 H, Me, main isomer), 1.51 (s, 6 H, CMe_2, minor isomer), 1.40 (s, 6 H, CMe_2, main isomer), 1.30 (s, 3 H, Me, minor isomer). ^{13}C{^{1}H}-NMR (100.8 MHz, $CDCl_3$): δ 156.0, 155.9, 153.7, 153.4, 151.5, 151.5, 147.1, 146.1, 137.8, 137.5, 137.0 (Ar C_q), 130.1, 130.0, 128.4, 128.2, 126.2, 126.1, 125.5, 125.4, 125.3, 125.2, 124.9, 124.1, 121.4, 121.3 (Ar C_q), 121.1, 120.9, 120.1, 119.9, 119.9, 118.9 (Ar CH), 73.2, 72.8 (C–O), 49.1, 48.6 (CH_2), 47.2, 47.1, 36.0, 34.5 (*C*Me), 32.9, 31.6, 27.1, 27.0 (Me). One signal

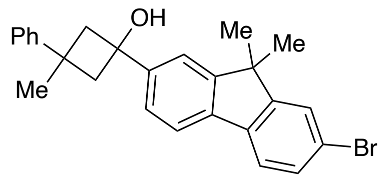

of both diastereoisomers is overlapped. HR-MS (+ESI) *m/z* calculated for $C_{26}H_{24}Br$ [M-OH]$^+$ 415.1061, found 415.1056.

1-(4'-iodo-[1,1'-biphenyl]-4-yl)-3-methyl-3-phenylcyclobutan-1-ol (4). It was obtained

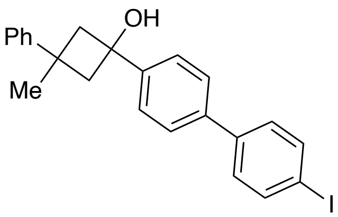

from 4,4'-diiodo-1,1'-biphenyl (1340 mg, 3.30 mmol, 1.13 equiv.), isopropylmagnesium chloride (1.5 mL, 3.10 mmol, 1.06 equiv.) and 3-methyl-3-phenylcyclobutan-1-one (468 mg, 2.93 mmol, 1.00 equiv.), by following a modification of the representative procedure (a mixture of dry THF and dry Et_2O in 9:1 proportion was used as solvent and the addition of isopropylmagnesium chloride was carried out at -30 ºC). After 16 hours of reaction time, the product was isolated pure by flash column chromatography with *n*-hexane:EtOAc as eluents (708 mg, 1.61 mmol, 54%). The isolated white solid was a mixture of diastereoisomers in approx. 1:0.6 ratio. IR (cm^{-1}): ν 3322 (ν(OH)), 2974, 2928, 1477, 1442, 1382, 1238, 1166, 1030, 996, 810, 761, 696, 575, 545, 511. ^{1}H-NMR (300.1 MHz, $CDCl_3$): δ 7.78−7.14 (several m, 13 H, Ar), 3.02 (vd, *J* = 13.5 Hz, 2 H, main isomer), 2.98 (vd, *J* = 12.4 Hz, 2 H, CH_2, minor isomer), 2.91 (vd, *J* = 12.4 Hz, 2 H, CH_2, minor isomer), 2.65 (vd, *J* = 13.5 Hz, 2 H, CH_2, main isomer), 2.02 (s, 1 H, OH, main isomer), 1.89 (s, 1 H, OH, minor isomer), 1.73 (s, 3 H, Me, main isomer), 1.30 (s, 1 H, Me, minor isomer). $^{13}C\{^1H\}$-NMR (75.5 MHz, $CDCl_3$): 151.5, 151.4, 146.9, 145.9, 140.2, 139.1, 138.7 (Ar C_q), 137.9, 137.8, 128.9, 128.9, 128.4, 128.2, 127.1, 126.8, 126.3, 125.5, 125.4, 125.3, 125.3, 125.1 (Ar CH), 93.1, 93.0 (C–I), 72.8, 72.3 (C–O), 49.0, 48.6 (CH_2), 36.0, 34.5 (*C*Me), 32.8, 31.5 (Me). Two C_{Ar} signals are overlapped. HR-MS (+ESI) *m/z* calculated for $C_{23}H_{20}I$ [M-OH]$^+$ 423.0604, found 423.0611.

1-(3-bromophenyl)-3-methyl-3-phenylcyclobutan-1-ol (5) was obtained from 1,3-

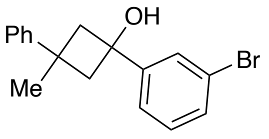

dibromobenzene (743 mg, 3.15 mmol, 1.05 equiv.), isopropylmagnesium chloride (2.1 mL, 3.2 mmol, 1.07 equiv.) and 3-methyl-3-phenylcyclobutan-1-one (481 mg, 3.00 mmol, 1.00 equiv.), by following a modification of the representative procedure (the addition of isopropylmagnesium chloride was carried out at 0 ºC, the bath temperature was increased to RT, and the mixture was further stirred for 2 hours before the addition to 3-methyl-3-phenylcyclobutan-1-one). After 16 hours of reaction time, the product was isolated by precipitation (332 mg, 1.05 mmol, 35%). The isolated white solid was a mixture of diastereoisomers in approx. 1:1 ratio. IR (cm^{-1}): ν 3327 (ν(OH)), 3247, 2976, 2951, 2930, 1596, 1561, 1492, 1443, 1415, 1245, 1026, 783, 762, 734, 699, 689. ^{1}H-NMR (300.1 MHz,

$CDCl_3$): δ 7.69 (t, $^4J_{HH}$ = 1.9 Hz, 1 H, Ar), 7.49 (t, $^4J_{HH}$ = 1.9 Hz, 1 H, Ar), 7.47−7.12 (several m, 8 H, Ar), 2.97–2.85 (m, 6 H, CH_2), 2.60 (vd, J = 13.4 Hz, 2 H, CH_2), 2.01 (s, 1 H, OH), 1.89 (s, 1 H, OH), 1.71 (s, 3 H, Me), 1.29 (s, 3 H, Me). $^{13}C\{^1H\}$-NMR (75.5 MHz, $CDCl_3$): δ 151.3, 151.1, 149.6, 148.6 (Ar C_q), 130.5, 130.2, 130.1, 130.0, 129.1, 128.4, 128.3, 128.0, 125.6, 125.5, 125.2, 125.0, 124.3, 123.5 (Ar CH), 122.8, 122.6 (Ar C_q), 72.6, 72.2 (C–O), 49.0, 48.4 (CH_2), 36.1, 34.5 (*C*Me), 32.7, 31.6 (Me). GC-MS *m/z* calculated for $C_{17}H_{17}BrO$ $[M]^+$ 316.0457, found 316.0452.

3. Synthesis and characterization of the polymers

3.1. Representative polymerization procedure

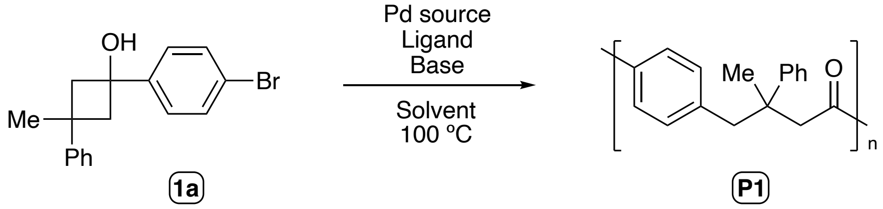

Cyclobutanol **1a** (0.5 mmol, 1 equiv.), the catalytic precursor, the ligand, and Cs_2CO_3 (1.1 equiv) were placed in a Carius tube under N_2 atmosphere. Dry toluene (3 mL) was added and the tube was sealed. The mixture was stirred and heated in an oil bath at 100 ºC for 16 hours. The resulting suspension was diluted with dichloromethane (10 mL), filtered over Celite, concentrated in vacuum up to ca. 1 mL and precipitated in methanol (20 mL). The obtained solid was filtered and dried under reduced pressure.

3.2. Optimization of the polymerization reaction

Only the displayed parameters in each table were modified. The other parameters were kept constant at the values indicated in the representative polymerization procedure. The yields were calculated from the isolated polymers masses by assuming a $(\text{monomer})_n$ composition, where "monomer" represents the cyclobutanol precursor with formal loss of HX (HBr or HI depending on the starting material).

Table S1. Influence of the catalytic precursor and the amount of added precursor.

Entry	Precursor (mol%)	Ligand (mol%)	Yield (%)	M_n (kDa)	M_w (kDa)	Đ
1	$[Pd(PPh_3)_4]$ (10)	-	34	1.9	3.0	1.53
2	$[Pd(PPh_3)_4]$ (1)	-	64	3.1	5.5	1.75
3	$Pd(dba)_2$ (2)	PPh_3 (5)	7	-	-	-
4	$Pd(dba)_2$ (2)	Bipy (5)	-	-	-	-
5	$Pd(OAc)_2$ (2)	PPh_3 (5)	88	3.6	6.3	1.75
6	$Pd(OAc)_2$ (2)	PPh_3 (2)	78	4.3	7.3	1.70
7	$Pd(OAc)_2$ (1)	PPh_3 (2)	78	5.7	10.9	1.92
8	$Pd(OAc)_2$ (0.5)	PPh_3 (1)	85	6.7	17.5	2.59
9	$Pd(OAc)_2$ (0.25)	PPh_3 (0.5)	62	2.8	5.3	1.87
10	$Pd(OAc)_2$ (0.125)	PPh_3 (0.25)	97	3.9	9.4	2.38
11	$Pd(OAc)_2$ (5)	PPh_3 (10)	82	4.5	7.4	1.63
12	$Pd(OAc)_2$ (10)	PPh_3 (20)	58	4.1	5.9	1.45
13	$Pd(OAc)_2$ (20)	PPh_3 (40)	66	0.11	0.74	6.51
14	$Pd(OAc)_2$ (2)	-	-	-	-	-
15	-	PPh_3 (4)	-	-	-	-
16	$[NiCl_2(PPh_3)_2]$ (2)	-	-	-	-	-
17	$[Ni(acac)_2]$ (2)	PMe_2Ph (6)	-	-	-	-
18	$[Ni(P(OEt)_3)_4]$ (3)	-	-	-	-	-

Table S2. Influence of the ligand.

Entry	Pd source (mol%)	Ligand (mol%)	Yield (%)	M_n (kDa)	M_w (kDa)	Đ
1	$Pd(OAc)_2$ (2)	PPh_3 (5)	88	3.6	6.3	1.75
2	$Pd(OAc)_2$ (2)	DPPF (2)	89	2.9	5.7	1.96
3	$Pd(OAc)_2$ (2)	PCy_3 (5)	71	2.8	4.6	1.61
4	$Pd(OAc)_2$ (2)	NHC (3)	-	-	-	-
5	$Pd(OAc)_2$ (2)	DPPE (3)	71	3.2	5.8	1.84
6	$Pd(OAc)_2$ (2)	DPE (3)	90	3.6	7.12	2.00
7	$Pd(OAc)_2$ (0.25)	DPE (0.3)	81	4.1	7.7	1.86
8	$Pd(OAc)_2$ (2)	JohnPhos (5)	-	-	-	-
9	$Pd(OAc)_2$ (1)	$P(4\text{-}OMe\text{-}Ph)_3$ (2)	85	4.4	10.8	2.43
10	$Pd(OAc)_2$ (1)	$P(n\text{-}Bu)_3$ (2)	63	1.1	1.7	1.49

DPPF = 1,1´-bis(diphenylphosphino)ferrocene
NHC = 1,3-bis-(2,6-diisopropylphenyl)imidazolium chloride
DPPE = 1,2-bis(diphenylphosphino)ethane
DPE = bis[(2-diphenylphosphino)phenyl]ether

Table S3. Influence of the base.

Entry	Pd source (mol%)	Ligand (mol%)	Base (equiv.)	Yield (%)	M_n (kDa)	M_w (kDa)	Đ
1	$Pd(OAc)_2$ (2)	PPh_3 (5)	Cs_2CO_3	88	3.6	6.3	1.75
2	$Pd(OAc)_2$ (2)	PPh_3 (5)	^{t}BuOK (1.5)	89	6.4	11.6	1.82
3	$Pd(OAc)_2$ (0.5)	PPh_3 (1)	CsF (1.5)	71	4.2	7.5	1.75
4	$Pd(OAc)_2$ (2)	PPh_3 (4)	Et_3N (1.1)	-	-	-	-
5	$Pd(OAc)_2$ (2)	PPh_3 (4)	K_2CO_3 (1.1)	72	2.9	4.5	1.55
6	$Pd(OAc)_2$ (2)	PPh_3 (4)	NaH (1.1)	29	1.9	2.5	1.34
7	$Pd(OAc)_2$ (1)	PPh_3 (2)	K_2HPO_4 (1.1)	-	-	-	-
8	$Pd(OAc)_2$ (2)	PPh_3 (4)	-	-	-	-	-

Table S4. Influence of the solvent.

Entry	Pd source (mol%)	Ligand (mol%)	Solvent	Yield (%)	M_n (kDa)	M_w (kDa)	Đ
1	$Pd(OAc)_2$ (2)	PPh_3 (5)	Toluene	88	3.6	6.3	1.75
2	$Pd(OAc)_2$ (2)	PPh_3 (3)	DMF	-	-	-	-
3	$Pd(OAc)_2$ (2)	PPh_3 (5)	1,4-dioxane	80	3.4	5.5	1.62
4	$Pd(OAc)_2$ (0.25)	PPh_3 (0.5)	1,4-dioxane	79	4.5	9.2	2.04
5	$Pd(OAc)_2$ (2)	PPh_3 (5)	MeCN	-	-	-	-
6	$Pd(OAc)_2$ (2)	PPh_3 (5)	1,2-DCE	74	2.5	3.9	1.56
7	$Pd(OAc)_2$ (2)	PPh_3 (5)	THF	63	2.7	4.2	1.57
8	$Pd(OAc)_2$ (2)	PPh_3 (5)	^{t}AmylOH	57	1.9	3.2	1.69
9	$Pd(OAc)_2$ (2)	PPh_3 (5)	NMP	-	-	-	-
10	$Pd(OAc)_2$ (2)	PPh_3 (5)	C_6H_5Cl	79	2.9	4.5	1.57

Table S5. Influence of the temperature and the concentration. ($Pd(OAc)_2$ [2 mol%] was used as catalyst).

Entry	Ligand (mol%)	T (ºC)	[**1**] (M)	Yield (%)	M_n (kDa)	M_w (kDa)	Đ
1	PPh_3 (5)	100	0.17	88	3.6	6.3	1.75
2	PPh_3 (4)	80	0.17	97	-	-	-
3	PPh_3 (5)	135	0.17	68	1.1	3.9	3.44
4	PPh_3 (4)	100	0.025	71	0.26	0.63	2.38

3.3. Scope of the polymerization reaction

These reactions were carried out by following the representative procedure with 1 mol% of $Pd(OAc)_2$, 2 mol% of PPh_3, 1.1 equiv. of Cs_2CO_3 and 3 mL of dry toluene as solvent.

Table S6. Scope of the polymerization reaction.

Substrate	Product	Yield (%)	M_n (kDa)	M_w (kDa)	Đ
**1b**[a]	**P1**	72	3.1	5.9	1.94
**2**	**P2**	94	2.3	3.6	1.58
**3**	**P3**	68	4.3	10.2	2.35
**4**	**P4**	73	5.7	14.6	2.55
**5**	**P5**	68	2.9	7.1	2.41
**6**[b]	**P6**	41	0.34	0.97	2.86

[a] The reaction was carried out with 0.5 mol% of $Pd(OAc)_2$ and 1 mol% of PPh_3.
[b] The reaction was carried out with 7 mol% of $Pd(OAc)_2$ and 14 mol% of PPh_3.

3.4. Characterization of the polymers

3.4.1. Spectroscopic and analytic data

Polymer P1. IR (cm^{-1}): ν 2970, 1672 (br, ν(C=O)), 1602, 1218, 1182, 1007, 760, 697. ^{1}H-NMR (300.1 MHz, $CDCl_3$): δ 7.58 (vd, *J* = 8.2 Hz, 2 H), 7.27–7.10 (br m, 5 H), 6.81 (vd, *J* = 8.0 Hz, 2 H), 3.43 (vd, *J* = 16.6 Hz, 1 H, CH_2), 3.26–3.10 (m, 3 H, CH_2), 1.44 (br s, 3 H, Me). $^{13}C\{^1H\}$-NMR (75.5 MHz, $CDCl_3$): δ 198.3 (C=O), 146.2, 143.7, 136.1 (C_q), 130.7, 128.1, 127.3, 126.1, 126.0 (Ar), 48.6, 47.7 (CH_2), 41.5 (*C*Me), 24.8 (Me). Anal. Calcd for $C_{17}H_{16}O$: C, 86.40; H, 6.82. Found: C, 86.49; H, 6.79. T_{dec}: 374 ºC. T_g: 122 ºC.

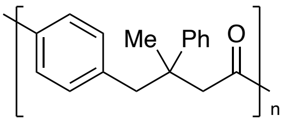

Polymer P2. IR (cm^{-1}): ν 3058, 3027, 1672 (br, ν(C=O)), 1603, 1496, 1443, 1413, 1354, 1205, 1184, 1001, 756, 697. ^{1}H-NMR (300.1 MHz, $CDCl_3$): δ 7.37–7.04 (m, 13 H, Ph), 6.55 (m, 1 H), 3.90–3.38 (m, 4 H, CH_2). $^{13}C\{^1H\}$-NMR (75.5 MHz, $CDCl_3$): δ 198.9 (C=O), 147.3, 143.6, 136.0, 130.7, 128.0, 127.8, 126.9, 126.1 (Ar), 49.7, 43.5, 42.8 (CH_2 and CPh_2). Only the data of the main set of signals are given. Smaller intensity signals were observed near every main signal, which are attributed to oligomers with different ending groups or additional unsaturations. T_{dec}: 338 ºC. T_g: 155 ºC.

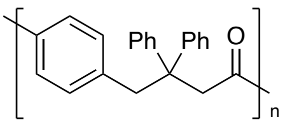

Polymer P3. IR (cm^{-1}): ν 2958, 2922, 1676 (br, ν(C=O)), 1604, 1466, 1442, 1415, 1191, 1078, 1031, 1007, 814, 767, 745, 698. ^{1}H-NMR (300.1 MHz,

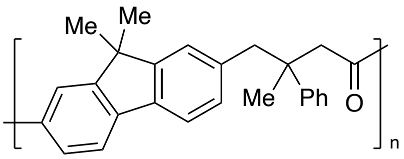

CDCl$_3$): δ 7.87–7.83 (m, 2 H), 7.62 (d, *J* = 8.0 Hz, 1 H), 7.52 (d, *J* = 7.8 Hz, 1 H), 7.35–7.15 (m, 5 H), 6.93 (d, *J* = 7.8 Hz, 1 H), 6.66 (s, 1 H), 3.64–3.58 (m, 1 H), 3.35–3.21 (m, 3 H), 1.53 (s, 3 H), 1.31 (s, 3 H), 1.18 (s, 3 H). ^{13}C{^{1}H}-NMR (75.5 MHz, CDCl$_3$): δ 198.3 (C=O), 154.1, 153.8, 146.8, 143.9, 138.8, 136.7, 135.9, 129.7, 128.1, 127.6, 126.3, 125.9, 125.1, 122.1, 119.9, 119.3 (Ar), 49.3, 47.8, 46.6, 41.7 (CH$_2$ and *C*Me), 26.7, 25.0 (Me). T_{dec}: 364 ºC. T_g: 168 ºC.

Polymer P4. IR (cm^{-1}): ν 3024, 2967, 2920, 1672 (br, ν(C=O)), 1597, 1494, 1443, 1396, 1354, 1214, 1193, 1003, 803, 764, 695. ^{1}H-NMR (300.1 MHz, CDCl$_3$): δ 7.89 (vd, *J* = 8.3 Hz, 2 H), 7.57 (vd, *J* = 8.2 Hz, 2 H), 7.38 (vd, *J* = 8.0 Hz, 2 H), 7.29–7.16 (m, 5 H), 6.91 (vd, *J* = 8.0 Hz, 2 H), 3.57 (vd, *J* = 16.5 Hz, 1 H), 3.30 (vd, *J* = 16.5 Hz, 1 H), 3.23 (br s, 2 H), 1.52 (br s, 3 H). ^{13}C{^{1}H}-NMR (75.5 MHz, CDCl$_3$): δ 198.2, 146.7, 145.2, 138.4, 137.5, 136.6, 131.2, 128.5, 128.1, 126.8, 126.4, 126.2, 126.0 (Ar), 48.5, 47.8 (CH$_2$), 41.5 (*C*Me), 25.1 (Me). T_{dec}: 387 ºC. T_g: 148 ºC.

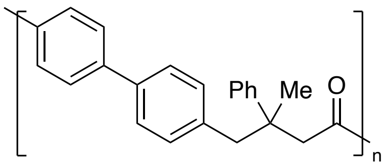

Polymer P5. IR (cm^{-1}): ν 3058, 3030, 2971, 2932, 1685 (br, ν(C=O)), 1602, 1580, 1497, 1442, 1355, 1244, 1181, 1028, 765, 694, 553. ^{1}H-NMR (300.1 MHz, CDCl$_3$): δ 7.64 (d, *J* = 7.6 Hz, 1 H), 7.25–6.95 (m, 8 H), 3.27–3.02 (m, 3 H, CH$_2$), 2.91 (br vd, *J* = 16.8 Hz, 1 H, CH$_2$), 1.36 (br s, 3 H, Me). ^{13}C{^{1}H}-NMR (75.5 MHz, CDCl$_3$): δ 198.5 (C=O), 146.3, 138.2, 137.3, 135.1, 130.1, 128.0, 127.8, 126.3, 126.1, 125.6 (Ar), 48.7, 47.6 (CH$_2$), 41.4 (*C*Me), 24.4 (Me). T_{dec}: 380 ºC. This polymer did not show a well-defined glass transition.

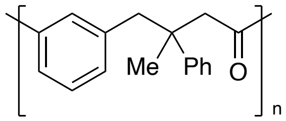

Polymer P6. IR (cm^{-1}): ν 3058, 1736 (br, ν(C=O)), 1656, 1599, 1446, 1286, 1263, 1164, 1099, 1031, 1004, 905, 825, 726, 646. ^{1}H-NMR (400.9 MHz, CDCl$_3$): δ 7.93–6.61 (br m, 12 H). ^{13}C{^{1}H}-NMR (100.8 MHz, CDCl$_3$): δ 196.2 (br, ν(C=O)), 150.4 (br), 132.1, 131.3–124.8 (set of broad aromatic CH signals), 120.2. T_{dec}: 280 ºC. This polymer did not show a well-defined glass transition.

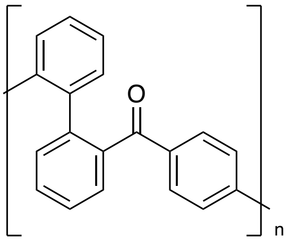

3.4.2. MALDI-TOF MS studies of the polymer end groups and chain termination mechanisms

Polymer P1

The MALDI-TOF mass spectrum of **P1** displayed a main polymeric series and several secondary series with much lower relative abundance (Figure S1). All of them showed the expected repeating unit ($C_{17}H_{16}O$, 236 Da). The mass values of the main series correspond to exact multiples of the repeating unit plus a Na^+ cation, which agrees with the formation of $(C_{17}H_{16}O)_n$ cyclic chains by head to tail cyclization (Scheme S1).

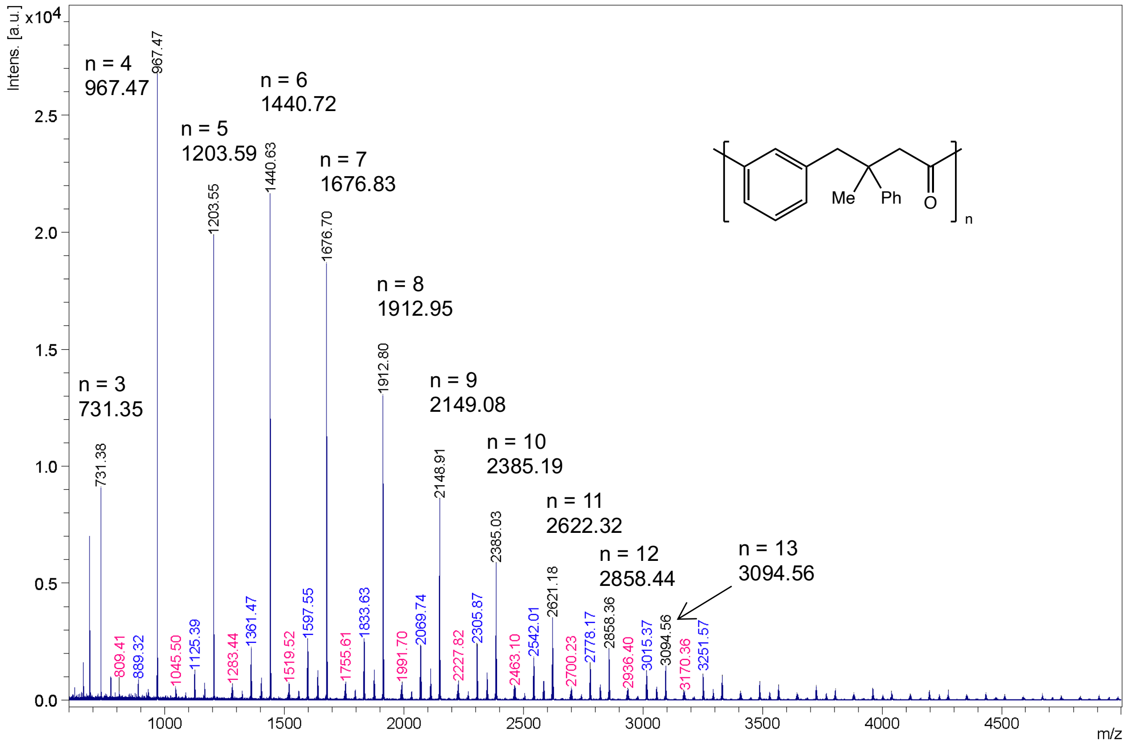

Figure S1. MALDI-TOF MS of **P1**. The *m*/*z* values of the most abundant peaks of the main polymeric series have been calculated according to the molecular formula $[(C_{17}H_{16}O)_nNa]^+$.

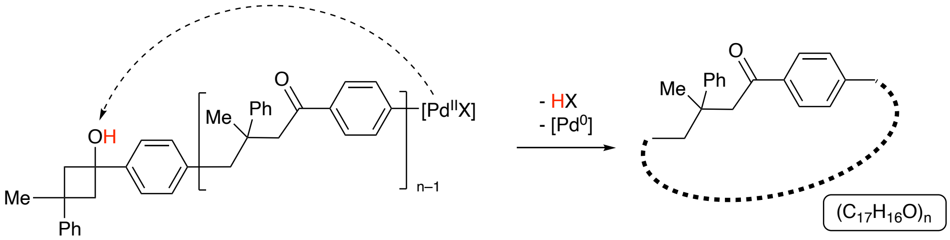

Scheme S1. Formation of cyclic polymers by head to tail cyclization.

However, on increasing *n*, head to tail cyclization becomes less likely than other chain termination events. Thus, instead of reacting with the terminal cyclobutanol group, the active

aryl-[Pd] chain end could metalate nearby phenyl groups and the resulting Pd(II) diaryl intermediate would undergo a reductive elimination (Scheme S2). This sequence of reactions would stop the polymer chain propagation with loss of a H atom, giving a shorter cycle. Then, the resulting polymers would have mass values equal to $(C_{17}H_{16}O)_n$ provided that on the other end of the chain there is an unreacted cyclobutanol moiety or other group with the same mass. The ^{1}H NMR spectrum of the polymer did not show signals with significant intensity in the region where the characteristic signals of the cyclobutanol group usually appear. However, the ^{1}H NMR spectrum of **P1** showed small signals at 1.49 and 3.29 ppm (Figure S23), which are compatible with the presence of a $C_6H_4C(O)CH_2CMe_2Ph$ end group. This group would be formed by opening of a terminal cyclobutanol, followed by a protodepalladation of the resulting intermediate (Scheme S2).

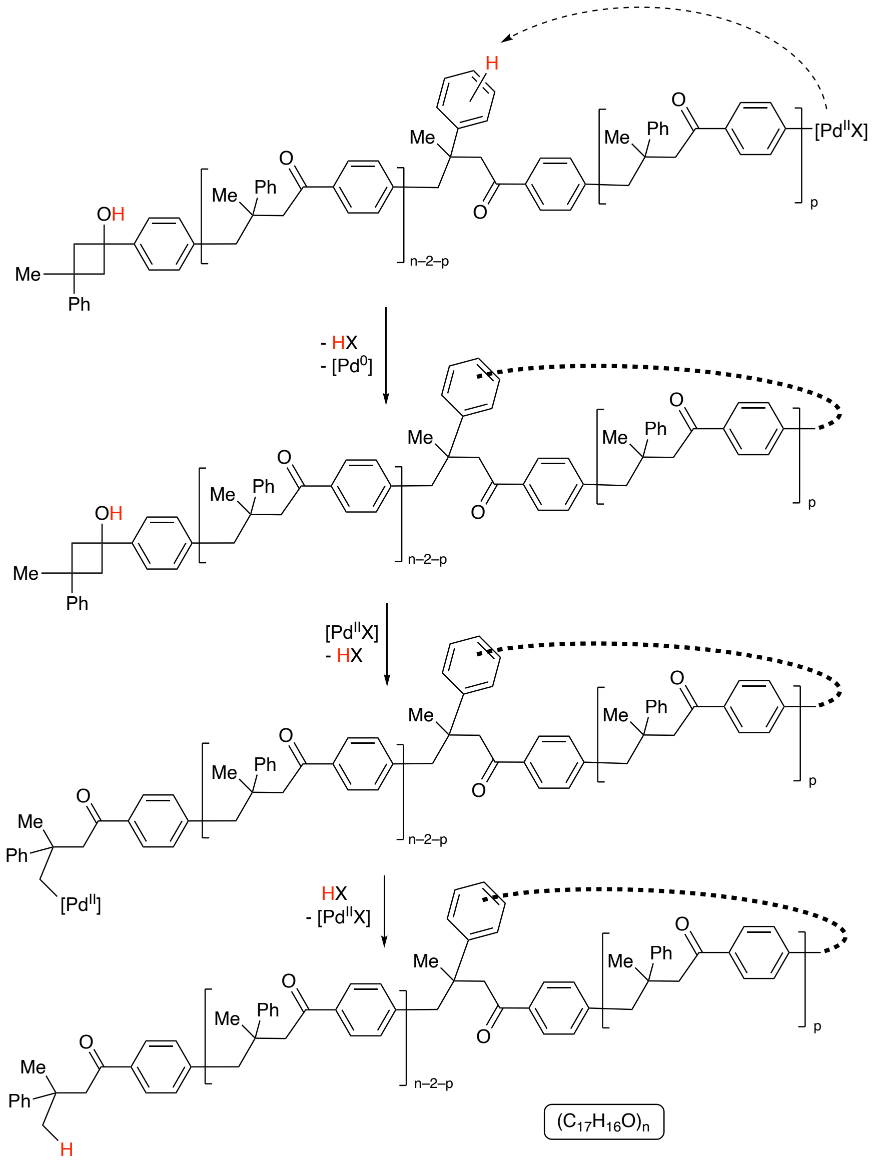

Scheme S2. C–H activation, C–C coupling and protodepalladation reactions that give rise to formation of $(C_{17}H_{16}O)_n$ polymers.

A second polymeric series with mass values 156 Da higher than those of the main series was observed (Figure S2, blue). These mass values and the observed isotopic distributions suggest that this series is formed by linear $(C_{17}H_{16}O)_n$ polymers with bromo and phenyl as end groups. The phenyl group may be incorporated by reductive elimination from an intermediate Pd(II) complex containing both a polymer chain and a phenyl ligand attached to the same metal center (Scheme S3). This suggests that, under the polymerization conditions, Ph-transfer from a PPh_3 ligand is possible.[4] In line with this hypothesis, when the polymerization was carried out with $P(4\text{-}MeOC_6H_4)_3$ instead of PPh_3 as auxiliary ligand, molecular ions containing $4\text{-}MeOC_6H_4$ groups were detected by MALDI in the secondary polymeric series (Figure S3). For $n \geq 11$, the relative abundances of this series becomes comparable to that of the main series.

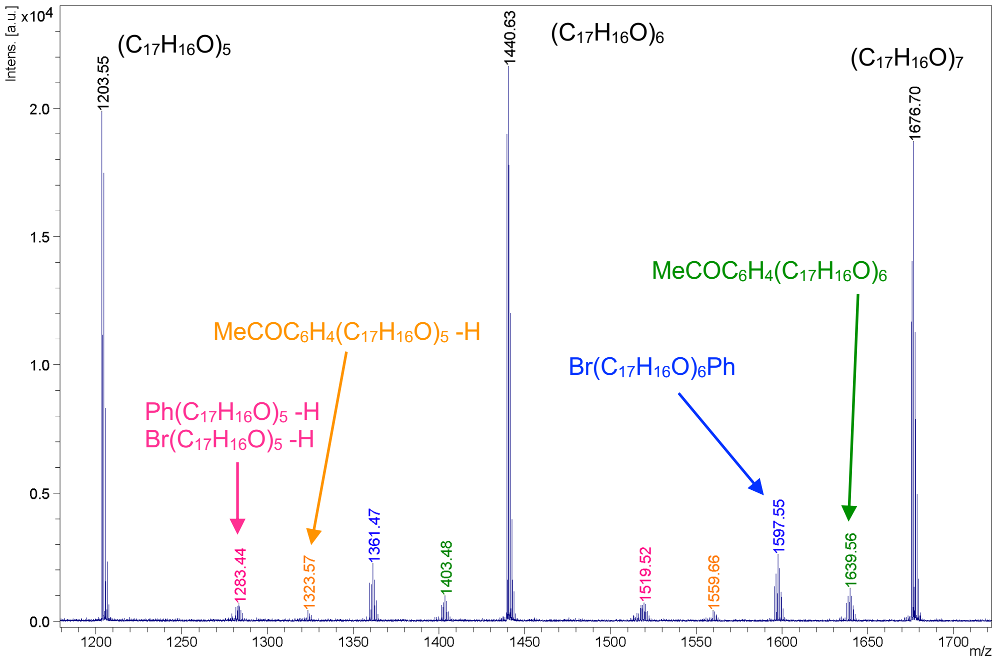

Figure S2. Detail of the MALDI-TOF MS of polymer **P1** showing the proposed composition for the main and secondary polymeric series. All observed *m*/*z* values correspond to the Na^+ adducts of the indicated molecules.

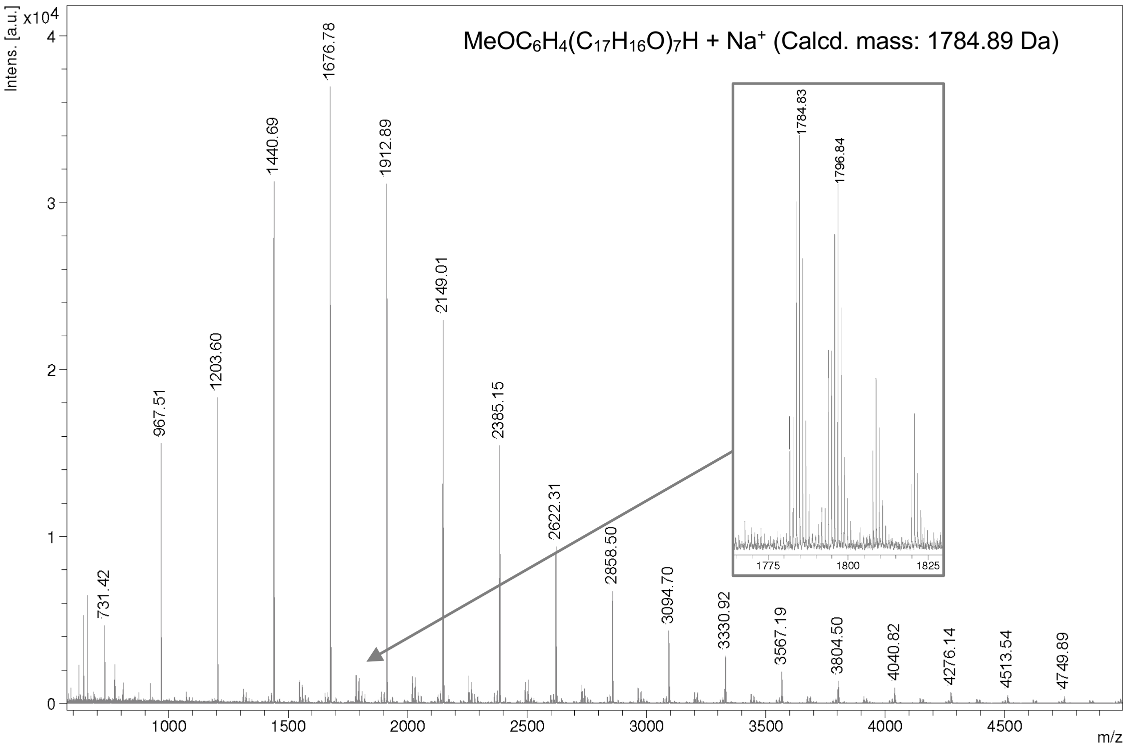

Figure S3. Detail of the MALDI-TOF MS of polymer **P1** obtained by using P(4-$MeOC_6H_4)_3$ instead of PPh_3. The inset shows a representative mass distribution of a molecular ion containing a 4-$MeOC_6H_4$ group.

A third series showing mass values 198 Da higher than the main series was also detected (Figure S2, green). The mass values and isotopic distributions of the peak clusters of this series agree with linear polymers with bromo and $C_6H_4C(O)CH_3$ end groups. The presence of the 4-acetylphenyl end group was corroborated by ^{1}H-NMR spectroscopy, and it is explained by a Pd-mediated [2+2]-retrocyclization of a terminal cyclobutanol unit, which generates 1-methylstyrene and a 4-acetylphenyl end group (Scheme S3, right). This double C-C cleavage process would compete with the single C-C cleavage which gives rise to opening of the cyclobutanol ring. We have recently reported that Pd-catalyzed cyclobutanol retrocyclization takes place almost exclusively when Johnphos ligand was used.[2] A low-abundance polymer series containing H and $C_6H_4C(O)CH_3$ as end groups was also observed (Figure S2, orange). In this case, the terminal 4-bromophenyl group would undergo oxidative addition to a Pd(0) species followed by protodepalladation of the resulting aryl-Pd(II) intermediate (Scheme S3, left).

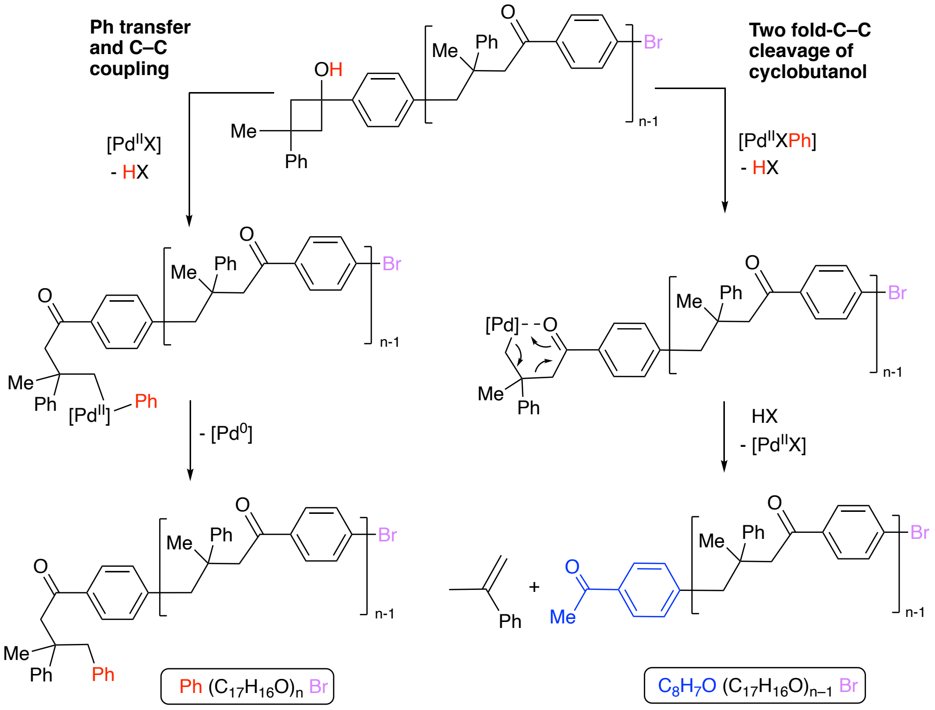

Scheme S3. Proposed structures and chain termination reactions for the second and third polymer series.

Finally, a fourth series formed by groups of peaks with very low abundance and a complex isotopic pattern was observed (Figure S2, red). The masses and isotopic distributions of these peak clusters are compatible with an overlap of several isotopic distributions corresponding to linear polymers containing *(i)* a cyclic group resulting from C-H activation at one end of the chain and Br or Ph at the other chain end and *(ii)* Ph and unreacted cyclobutanol end groups (Scheme S4).

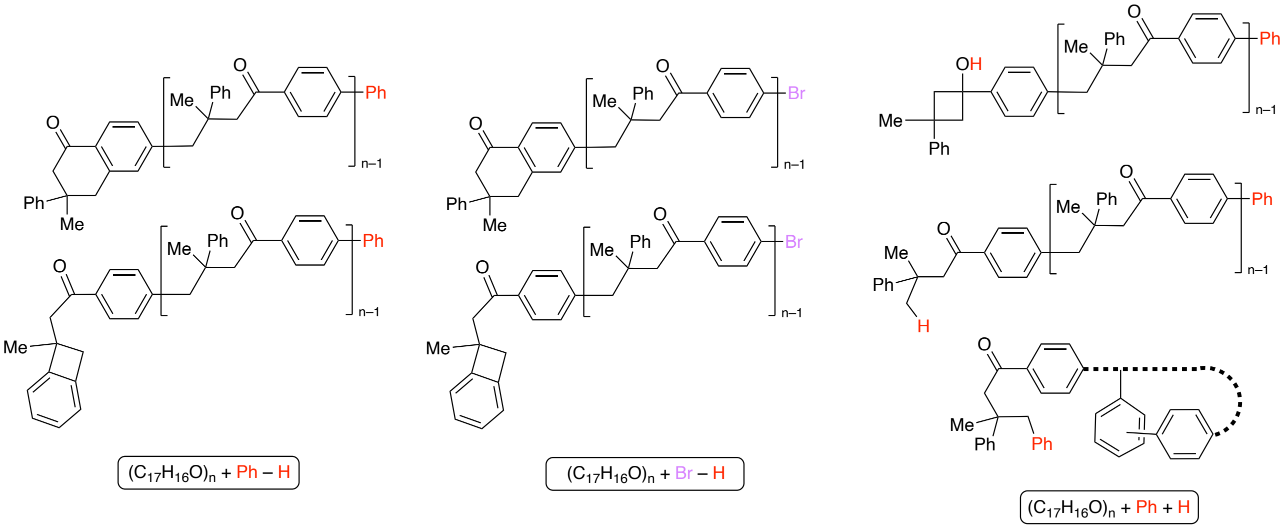

Scheme S4. Proposed combinations of end groups for the fourth polymeric series.

Polymer P2

This polymer showed the expected series of peaks with a $(C_{22}H_{18}O)_n$ composition, corresponding to cyclic chains or to linear chains with protonated and unsaturated end groups (Figure S4). The abundances of the secondary polymeric series were much lower than in the mass spectrum of **P1**. A detailed analysis of the isotopic distributions of the main series (Figure S5) revealed a complex pattern resulting from an overlap of the expected isotopic distribution for the $[(C_{22}H_{18}O)_nNa]^+$ ions with two distributions corresponding to ions containing two more or two less H atoms. This suggests that, in addition to the main chain-termination mechanisms (see polymer **P1**), other mechanisms involving protodepalladation or C-H activation events at both chain ends are competent. In line with this, the ^{1}H- and ^{13}C{^{1}H}-NMR spectra of this polymer show additional signals with lower intensity near the main set of signals.

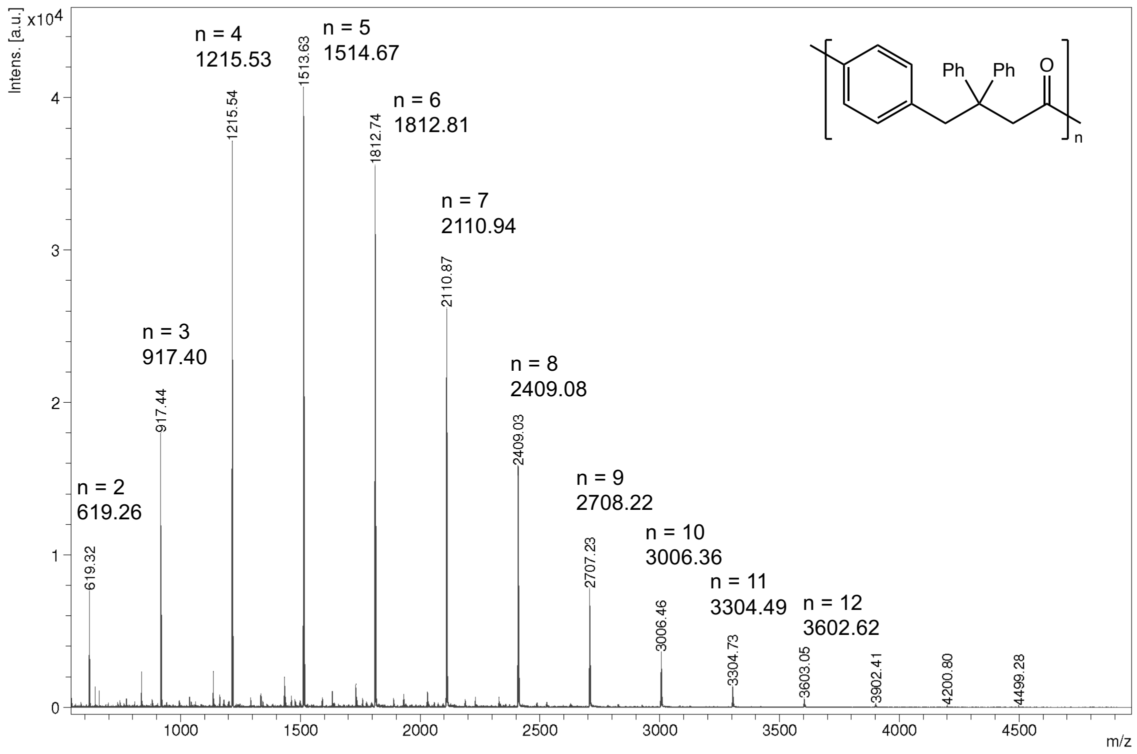

Figure S4. MALDI-TOF MS of **P2**. The *m*/*z* values of the most abundant peaks of the main polymeric series have been calculated according to the molecular formula $[(C_{22}H_{18}O)_nNa]^+$.

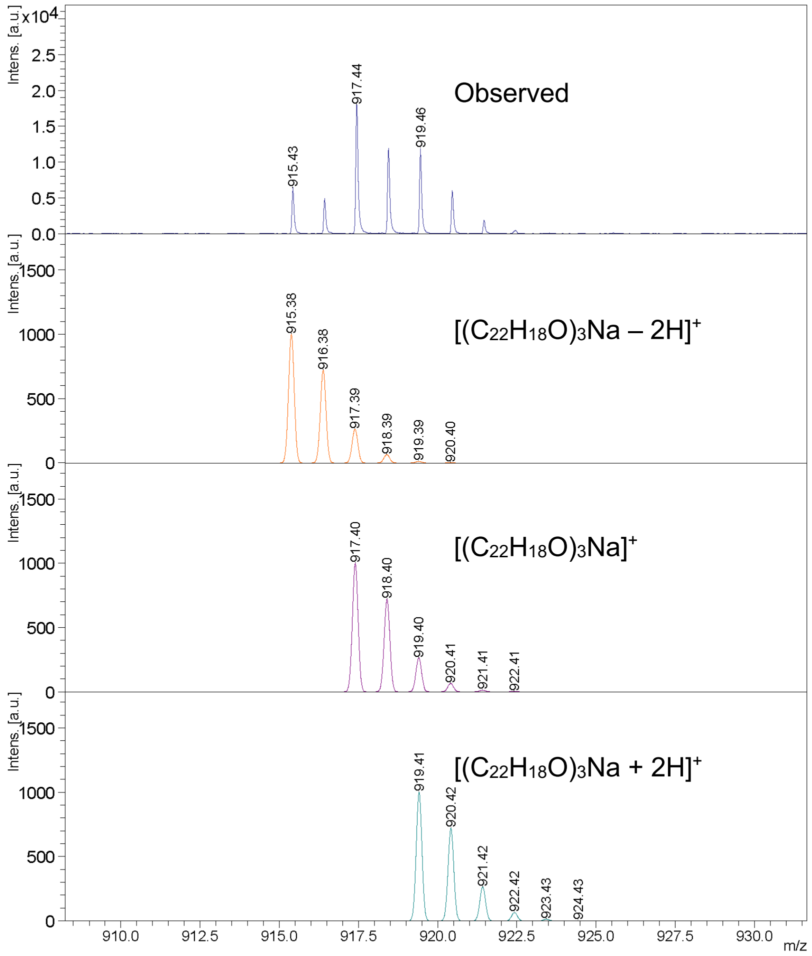

Figure S5. Comparison of the observed peak cluster around 918 Da of the MALDI-TOF MS of polymer **P2** with the simulated isotopic distributions of the ion $[(C_{22}H_{18}O)_3Na]^+$, and the ions resulting of removing or adding or two H atoms from it.

Polymer P3

The MALDI TOF mass spectrum of **P3** was similar to that of **P1**. It shows a main distribution corresponding to Na^+ adducts of the $(C_{26}H_{24}O)_n$ chains (Figure S6). The secondary series show very low abundance.

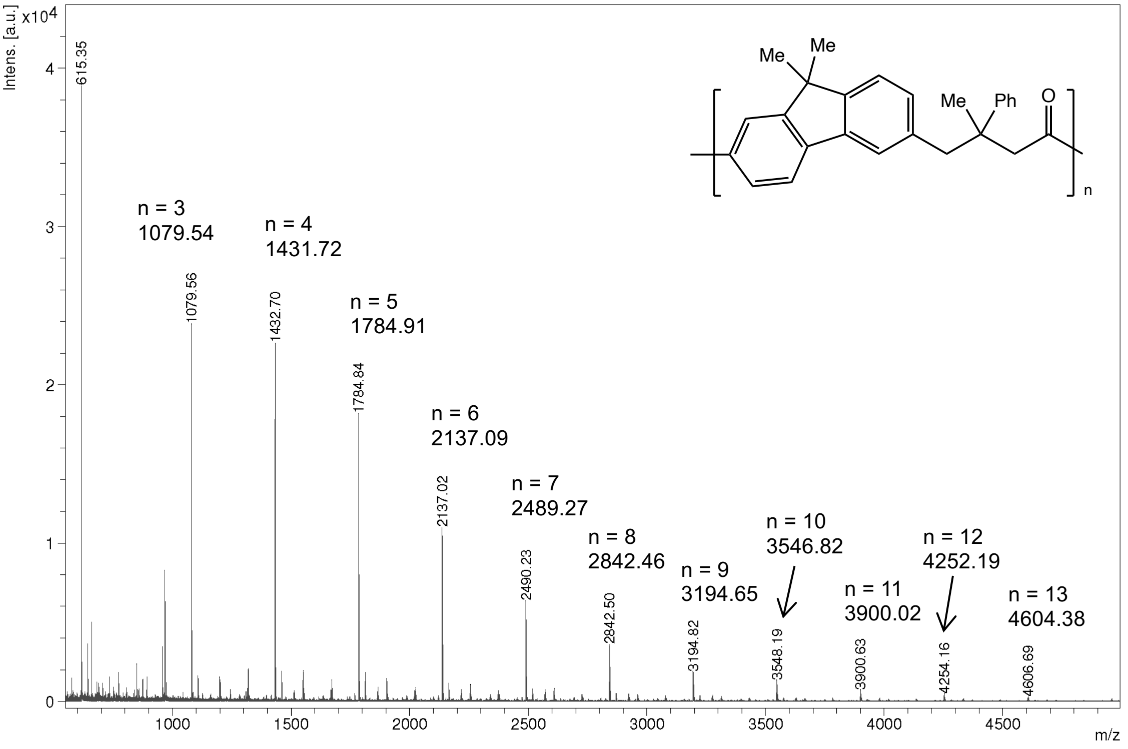

Figure S6. MALDI-TOF MS of **P3**. The *m/z* values of the most abundant peaks of the main polymeric series have been calculated according to the molecular formula $[(C_{26}H_{24}O)_nNa]^+$.

Polymer P4

The MALDI TOF mass spectrum of **P4** shows a main series of $[(C_{23}H_{20}O)_nNa]^+$ ions and two secondary series of chains with (a) Ph and H, (b) $MeCOC_6H_4$ and H as terminal groups (Figures S7 and S8).

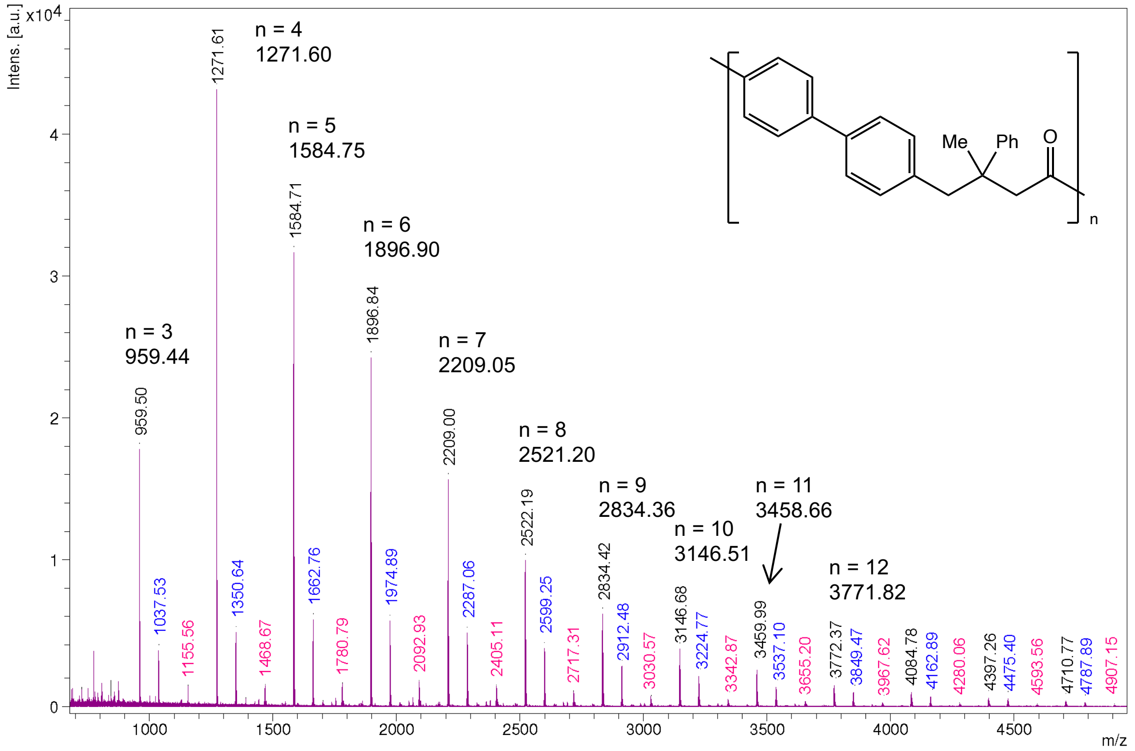

Figure S7. MALDI-TOF MS of polymer **P4**. The *m/z* values of the most abundant peaks of the main polymeric series have been calculated according to the molecular formula $[(C_{23}H_{20}O)_nNa]^+$.

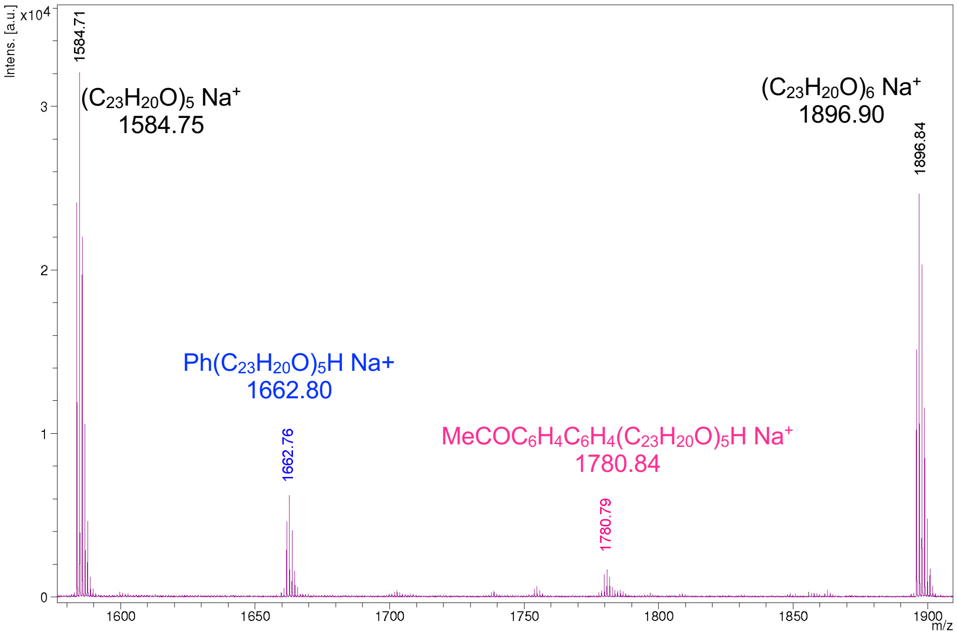

Figure S8. Zoom of the MALDI-TOF MS of polymer **P4** showing the proposed composition and calculated masses for the significant peaks of the main and secondary polymeric series.

Polymer P5

The MALDI-TOF mass spectrum of this polymer closely resembles that of its isomer **P1**. The main series is formed by $(C_{17}H_{16}O)_n$ chains (Figure S9). The secondary series are mainly formed by $(C_{17}H_{16}O)_n$ chains with (a) Ph and H, (b) $MeCOC_6H_4$ and H, (c) $MeCOC_6H_4$ and Ph as terminal groups (Figure S10).

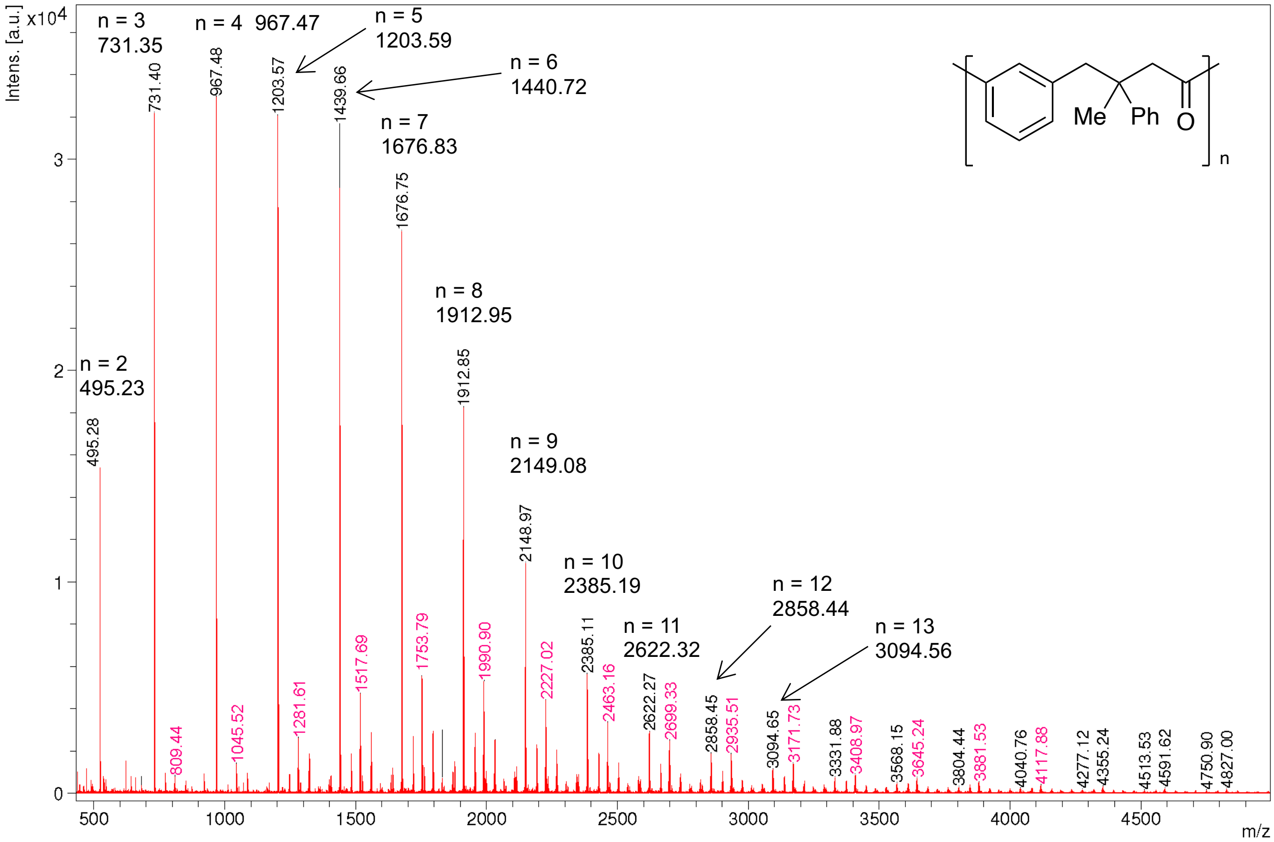

Figure S9. MALDI-TOF MS of polymer **P5**. The *m*/*z* values of the most abundant peaks of the main polymeric series have been calculated according to the molecular formula $[(C_{17}H_{16}O)_nNa]^+$.

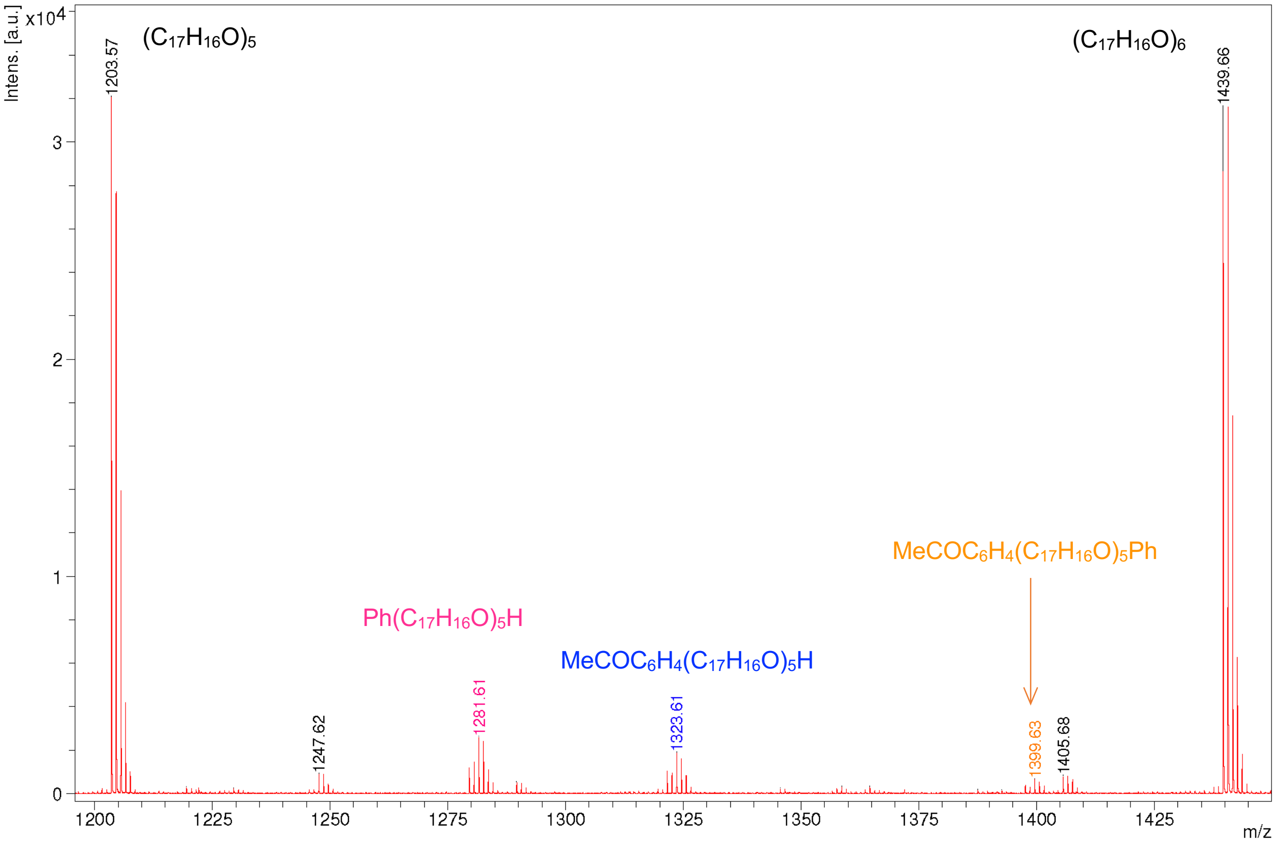

Figure S10. Detail of the MALDI-TOF MS of polymer **P5** showing the proposed composition for the main and secondary polymeric series. All observed *m*/*z* values correspond to the Na^+ adducts of the indicated molecules.

Polymer P6

The MALDI-TOF mass spectrum of this polymer shows two main series of ions corresponding to linear oligomers with either (a) two H atoms or (b) a H atom and a Ph group as terminal groups. (Figure S11).

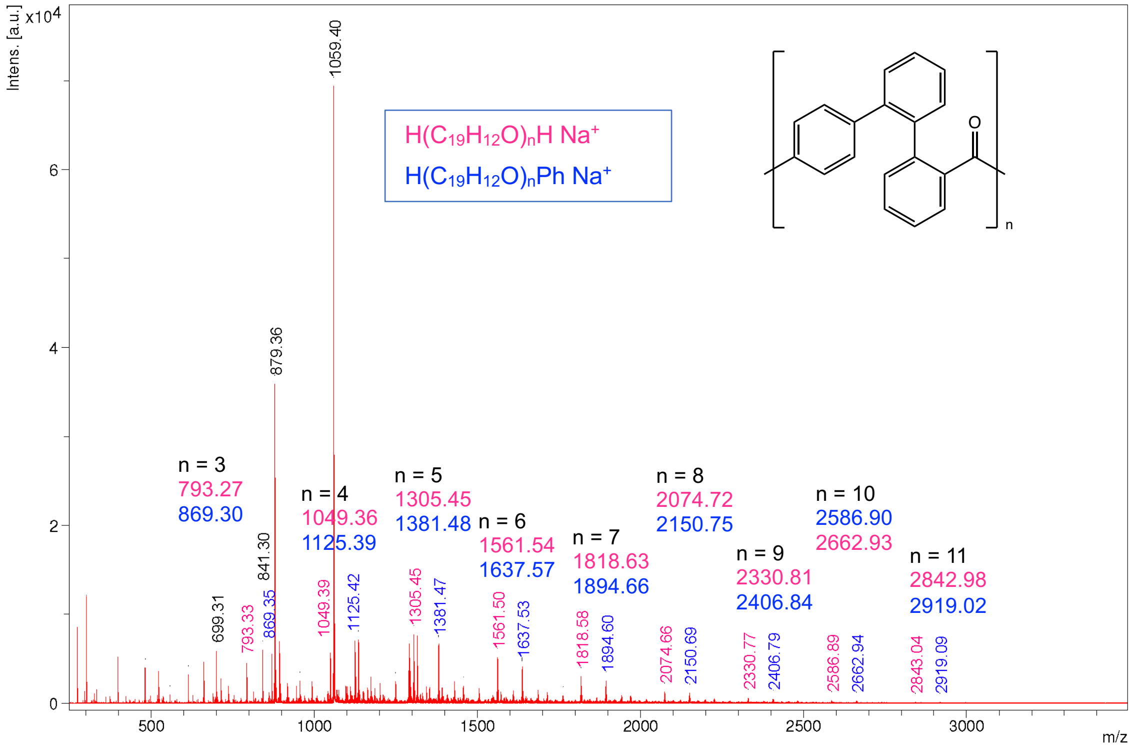

Figure S11. MALDI-TOF MS of polymer **P6**. The *m*/*z* values corresponding to the marked peak of each observed isotopic distribution have been calculated with the displayed formulae.

4. Mechanistic studies

4.1. Monitoring of the polymerization reaction

A mixture of cyclobutanol **1a** (158 mg, 0.50 mmol, 1 equiv.), $Pd(OAc)_2$ (1.12 mg, 5.0 × 10^{-3} mmol, 1 mol%), PPh_3 (2.62 mg, 0.01 mmol, 2 mol%), and Cs_2CO_3 (179 mg, 0.55 mmol, 1.1 equiv.) in dry toluene (3 mL) under N_2 atmosphere was heated in an oil silicon bath at 100 ºC in a Carius tube. 0.3 mL aliquots were taken from the reaction mixture at the times specified in the table S7. The samples were evaporated, dissolved in $CHCl_3$, and injected in the GPC chromatograph. After 16 hours, the reaction mixture was processed by following the isolation steps indicated in the general polymerization procedure.

The GPC chromatogram at short reaction time only showed low molecular weight components which were gradually consumed, with a consequent increase on the amount of polymeric material. From 3.5 to 16 h, the increase in the molecular weight of the formed polymer is unsignificant (Figure S12).

Table S7. Change of the average molecular weight of the polymer with the polymerization time.

Entry	Reaction time (h)	M_n (kDa)	M_w (kDa)	Đ
1	0.5	0.45	1.4	3.23
2	1.5	0.64	3.8	5.99
3	3.5	3.8	11.9	3.14
4	16	7.6	14.7	1.93

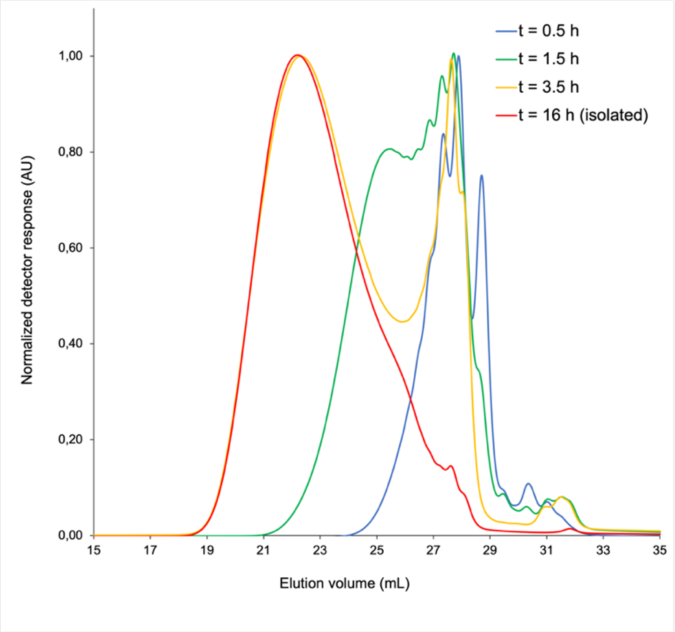

Figure S12. GPC traces of the polymerization reaction mixture of cyclobutanol **1a** at different reaction times and the isolated polymer.

4.2. Synthesis and characterization of intermediate complexes

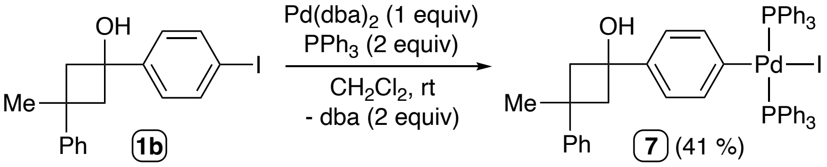

Complex 7. A Carius tube was charged with the monomer **1b** (200 mg, 0.549 mmol), [Pd(dba)$_2$] (315 mg, 0.549 mmol), PPh_3 (288 mg, 1.098 mmol) and a magnetic stirrer. The tube was set under a nitrogen atmosphere, and dry CH_2Cl_2 was added (10 mL). The tube was sealed, and the mixture was stirred at room temperature for 18 h. Partial decomposition to metallic palladium was observed. The mixture was filtered through a Celite plug. The filtrate was concentrated to ca. 0.5 mL, and Et_2O (15 mL) was added. The mixture was stirred in an ice bath until precipitation of a yellow solid took place. The suspension was filtered, and the solid was washed with cold Et_2O (2 $\times$ 2 mL) and air-dried to give crude **7** as a mixture of diastereoisomers (ratio ca. 1:1 by ^{1}H-NMR) containing a small amount of dba. The crude product was recrystallized from CH_2Cl_2/Et_2O to give a spectroscopically and analytically pure sample of **7**. The diastereoisomeric ratio of the purified sample was 1:2.4. Yield (crude): 224 mg, 0.225 mmol, 41 %. ^{1}H-NMR (300.1 MHz, $CDCl_3$): δ 7.60–7.43 (m, 24 H, C_6H_5), 7.36–7.12 (m, 46 H, C_6H_5), 6.74 (vdt, *J* = 8.1, *J* = 2.1 Hz, 2 H, C_6H_4, main diastereoisomer), 6.54 (vdt, *J* = 8.1, *J* = 2.1 Hz, 2 H, C_6H_4, minor diastereoisomer), 6.42 (vd, *J* = 8.4, Hz, 2 H, C_6H_4, main diastereoisomer), 6.42 (vd, *J* = 8.1, Hz, 2 H, C_6H_4, minor diastereoisomer), 2.68 (m, 4 H, CH_2, both diastereoisomers), 2.58 (vd, *J* = 12.8 Hz, 2 H, main diastereoisomer), 2.46 (vd, *J* = 13.5 Hz, 2 H, CH_2, minor diastereoisomer), 1.62 (s, 3 H, Me, minor diastereoisomer), 1.43 (s, 1 H, OH, minor diastereoisomer), 1.28 (s, 1 H, OH, main diastereoisomer), 1.18 (s, 3 H, Me, main diastereoisomer). ^{13}C{^{1}H}-NMR (75.5 MHz, $CDCl_3$): δ 158.8 (s, C_q, both diastereoisomers), 152.3 (s, C_q), 151.7 (s, C_q), 141.3 (s, C_q), 140.4 (s, C_q), 135.9 (vt, *J* = 5.1 Hz, CH, C_6H_4), 135.5 (vt, *J* = 5.0 Hz, CH, C_6H_4), 135.0 (vt, *J* = 6.5 Hz, *o*-CH, PPh_3), 134.9 (vt, *J* = 6.5 Hz, *o*-CH, PPh_3), 132.2 (vt, *J* = 23.2 Hz, *i*-C, PPh_3), 132.0 (vt, *J* = 23.2 Hz, *i*-C, PPh_3), 129.8 (s, *p*-CH, PPh_3), 129.7, (s, *p*-CH, PPh_3), 129.0 (s, *p*-CH, Ph), 128.4 (s, *p*-CH, Ph), 128.2 (s, CH, Ph), 128.1 (s, CH, Ph), 127.8 (vt, *J* = 5.3 Hz, *p*-CH, PPh_3), 127.7 (vt, *J* = 5.3 Hz, *p*-CH, PPh_3), 125.2 (s, CH, Ph), 125.0 (s, CH, Ph), 124.6 (s, CH, C_6H_4), 124.0 (s, CH, C_6H_4), 72.4 (s, C–OH), 72.2 (s, C–OH), 72.1 (s, C–OH), 71.9 (s, C–OH), 48.2 (s, CH_2, both diastereoisomers), 47.7 (s, CH_2, both diastereoisomers), 35.6 (s, C_q), 34.0 (s, C_q), 32.5 (s, Me), 31.8 (s, Me). Surprisingly, the ^{13}C signal corresponding to the quaternary carbon bonded to the alcohol group (C–OH) appeared split for both

diastereoisomers. We attribute this feature to a possible association of two molecules through hydrogen bond interaction in $CDCl_3$ solution. This splitting only affected to this carbon and was also observed in the $^{13}C\{^1H\}$-NMR spectrum of complex **8** in $CDCl_3$. For complex **8**, the splitting disappeared when the $^{13}C\{^1H\}$-NMR was recorded in DMSO-d_6. $^{31}P\{^1H\}$-NMR (121.5 MHz, $CDCl_3$): δ 20.8 (s), 20.6 (s). IR (cm^{-1}): ν 3561 (w), 3048 (w), 1477 (m), 1434 (s), 1092 (s), 1009 (m), 813 (m), 741 (s), 688 (vs), 512 (s), 492 (s), 456 (m). Anal. Calcd for $C_{53}H_{47}IOP_2Pd$: C, 63.96; H, 4.76. Found: C, 64.09; H, 4.73.

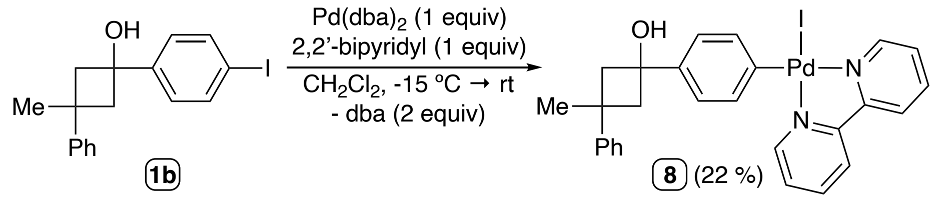

Complex 8. A Carius tube was charged with the monomer **1b** (200 mg, 0.549 mmol), [Pd(dba)$_2$] (318 mg, 0.553 mmol), 2,2'-bipyridyl (89 mg, 0.570 mmol) and a magnetic stirrer. The tube was set under a nitrogen atmosphere, placed in an ice/salt-bath, and dry CH_2Cl_2 was added (15 mL). The tube was sealed, and the mixture was stirred at –15 ºC for 30 min and then allowed to warm to room temperature, while stirring for another 4 h. Decomposition to metallic palladium was observed. The mixture was filtered through a Celite plug. The filtrate was concentrated to ca. 0.5 mL, and Et_2O (15 mL) was added. The suspension was filtered, and the pink solid was washed with Et_2O (2 × 3 mL) and air-dried to give complex **8** as a mixture of diastereoisomers (ca. 1:1 ratio by ^1H-NMR). Yield: 75 mg, 0.12 mmol, 22 %. ^1H-NMR (300.1 MHz, DMSO-d_6): δ 9.36 (br s, 2 H, CH), 8.61 (vt, J = 7.5 Hz, 4 H, CH), 8.26–8.23 (br m, 4 H, CH), 7.77 (br s, 2 H, CH), 7.57 (br s, 2 H, CH), 7.46 (br s, 2 H, CH), 7.37–7.13 (m, 16 H, CH), 6.93 (vd, J = 8.1 Hz, 2 H, CH), 5.37 (s, 1 H, OH), 5.15 (s, 1 H, OH), 2.86–2.77 (m, 4 H, CH_2), 2.68 (vd, J = 12.1 Hz, 2 H, CH_2), 1.63 (s, 3 H, Me), 1.22 (s, 3 H, Me). The signal corresponding to 2 H of CH_2 was obscured by the deuterated solvent. $^{13}C\{^1H\}$-NMR (75.5 MHz, DMSO-d_6): δ 155.5 (br s, C_q), 153.6 (br s, C_q), 152.5 (s, C_q), 152.0 (s, C_q), 151.3 (br s, CH), 148.8 (br s, CH), 144.4 (s, C_q), 143.6 (s, C_q), 143.4 (s, C_q), 142.5 (s, C_q), 139.9 (br s, CH), 135.7 (s, CH), 135.5 (s, CH), 128.2 (s, CH), 128.1 (s, CH), 127.3 (br s, CH), 127.0 (br s, CH), 125.1 (s, CH), 125.0 (s, CH), 124.7 (s, CH), 123.6 (s, CH), 123.1 (s, CH), 70.5 (s, C–OH), 70.1 (s, C–OH), 49.2 (s, CH_2), 35.6 (s, C_q), 33.7 (s, C_q), 33.0 (s, Me), 31.3 (s, Me). IR (cm^{-1}): ν 3428 (w), 1600 (m), 1471 (m), 1439 (m), 1100 (m), 812 (m), 761 (s), 728 (m), 702 (m), 552 (m), 417 (m). Anal. Calcd for $C_{27}H_{25}IN_2OPd$: C, 51.74; H,

4.02; N, 4.47. Found: C, 51.79; H, 4.27; N, 4.50. Single crystals of **8**·CH_2Cl_2, suitable for an X-ray diffraction study, were obtained by slow diffusion of *n*-pentane into a solution of **8** in CH_2Cl_2.

4.3. Crystal structure of complex 8

The crystal structure of **8**·CH_2Cl_2 was solved by X-ray diffraction studies (Figure S13). The palladium atom was in a square-planar environment, with a mean deviation of the Pd(II)-coordination plane of 0.016 Å. The N(1)−Pd(1)−N(2) angle was 79.03(15)°, quite smaller than the standard value of 90° for an ideal square-planar complex, due to the steric constraints imposed by the bite angle of the bipyridine ligand. The Pd(1)−N(1) bond length (2.130 Å; *trans* to C1) was significantly longer than the Pd(1)−N(2) bond length (2.087 Å; *trans* to I1), reflecting the greater *trans* influence of the *C*-donor ligand. The aryl ring (C1–C6) bonded to palladium formed an angle of 83.1° with respect to the Pd(II)-coordination plane, avoiding steric hindrance. The discrete molecules **8**·CH_2Cl_2 are associated through hydrogen bonds, giving double chains along the (110) direction.

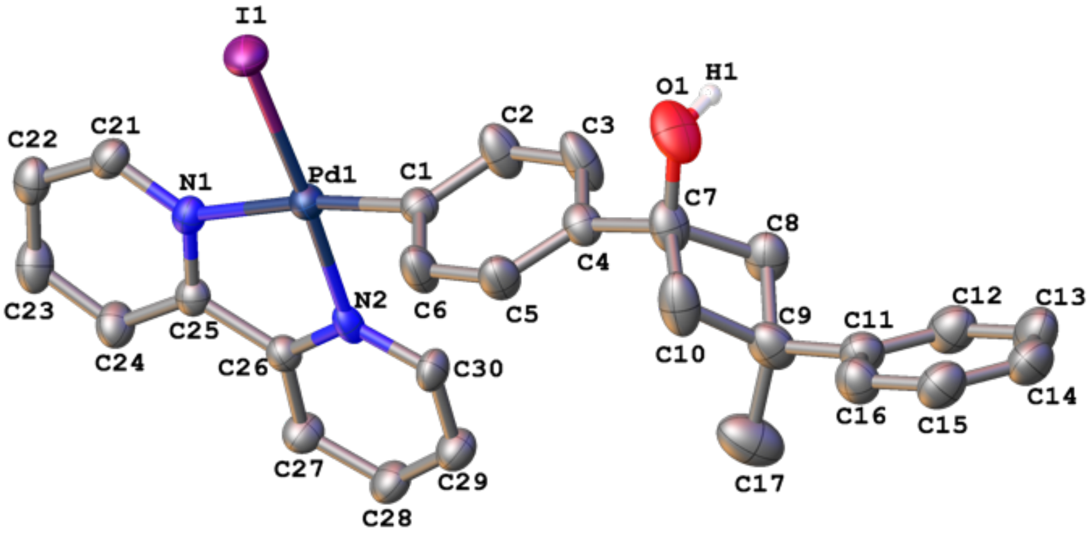

Figure S13. Thermal ellipsoid plot (50% probability) of complex **8**·CH_2Cl_2 along with the labeling scheme. The solvent molecule and the hydrogen atoms bonded to carbon have been omitted for clarity. Selected bond lengths (Å) and angles (deg): Pd1(1)−I(1) = 2.5655(6), Pd(1)−N(1) = 2.130(4), Pd(1)−N(2) = 2.087(4), Pd(1)−C(1) = 1.985(5), O(1)−C(7) = 1.452(8); I(1)−Pd(1)–N(1) = 87.35(15), N(1)−Pd(1)−N(2) = 79.03(15), N(2)−Pd(1)−C(1) = 94.63(18), C(1)−Pd(1)−I(1) = 98.87(12)

Data Collection. A crystal suitable for X-ray diffraction was mounted in inert oil on a glass fiber and transferred to a Bruker diffractometer. Data were recorded at 100(2) K, using

graphite-monochromated Mo-Kα radiation (λ = 0.71073 Å) and omega and phi scan mode. Multiscan absorption correction was applied.

Structure Solution and Refinements. The crystal structure was solved by dual method, and all non-hydrogen atoms were refined anisotropically on F^2 using the program SHELXL-2019/1. [5] Hydrogen atoms were refined as follows: methyl, rigid group; all others, riding.

Table S8. Crystal data and structure refinement for complex **8**·CH_2Cl_2.

**Empirical formula**	$C_{27.50}H_{26}ClIN_2OPd$
**Formula weight**	669.25
**Temperature**	100(2) K
**Wavelength**	0.71073 Å
**Crystal system**	Monoclinic
**Space group**	*C*2/*c*
**Unit cell dimensions**	a = 24.3564(15) Å α = 90°<br>b = 9.7658(6) Å β = 92.190(2)°<br>c = 23.2958(14) Å γ = 90°
**Volume**	5537.1(6) Å^3
Z	8
**Density (calculated)**	1.606 mg/m^3
**Absorption coefficient**	1.903 mm^{-1}
***F*(000)**	2632
**Crystal size**	0.380 x 0.110 x 0.030 mm^3
**Theta range for data collection**	1.750 to 28.834°
**Index ranges**	$-32 \le h \le 32$, $-13 \le k \le 13$, $-31 \le l \le 31$
**Reflections collected**	103 608
**Independent reflections**	7242 (R_{int} = 0.0418)
**Completeness to theta = 28.000°**	100.0 %
**Absorption correction**	Semi-empirical from equivalents
**Max. and min. transmission**	0.7458 and 0.6010
**Refinement method**	Full-matrix least-squares on F^2
**Data / restraints / parameters**	7242 / 0 / 318
**Goodness-of-fit on F^2**	1.155
**Final R indices (*I* > 2sigma(*I*))**	R1 = 0.0574, wR2 = 0.1147
**R indices (all data)**	R1 = 0.0735, wR2 = 0.1292
**Largest diff. peak and hole**	2.008 and –1.809 e·Å^{-3}

4.4. Polymerization reaction employing the intermediate complex 7 as initiator

This reaction was carried out with cyclobutanol **1a** (163 mg, 0.51 mmol, 1 equiv.), complex **7** (5.11 mg, 5.1×10^{-3} mmol, 1 mol%), Cs_2CO_3 (201 mg, 0.62 mmol, 1.2 equiv.), and dry toluene as solvent following the representative procedure for the polymerization reaction. Yield (%): 63. M_n: 4.7 kDa. M_w: 10 kDa. *Đ*: 2.18.

5. GPC traces of the polymers

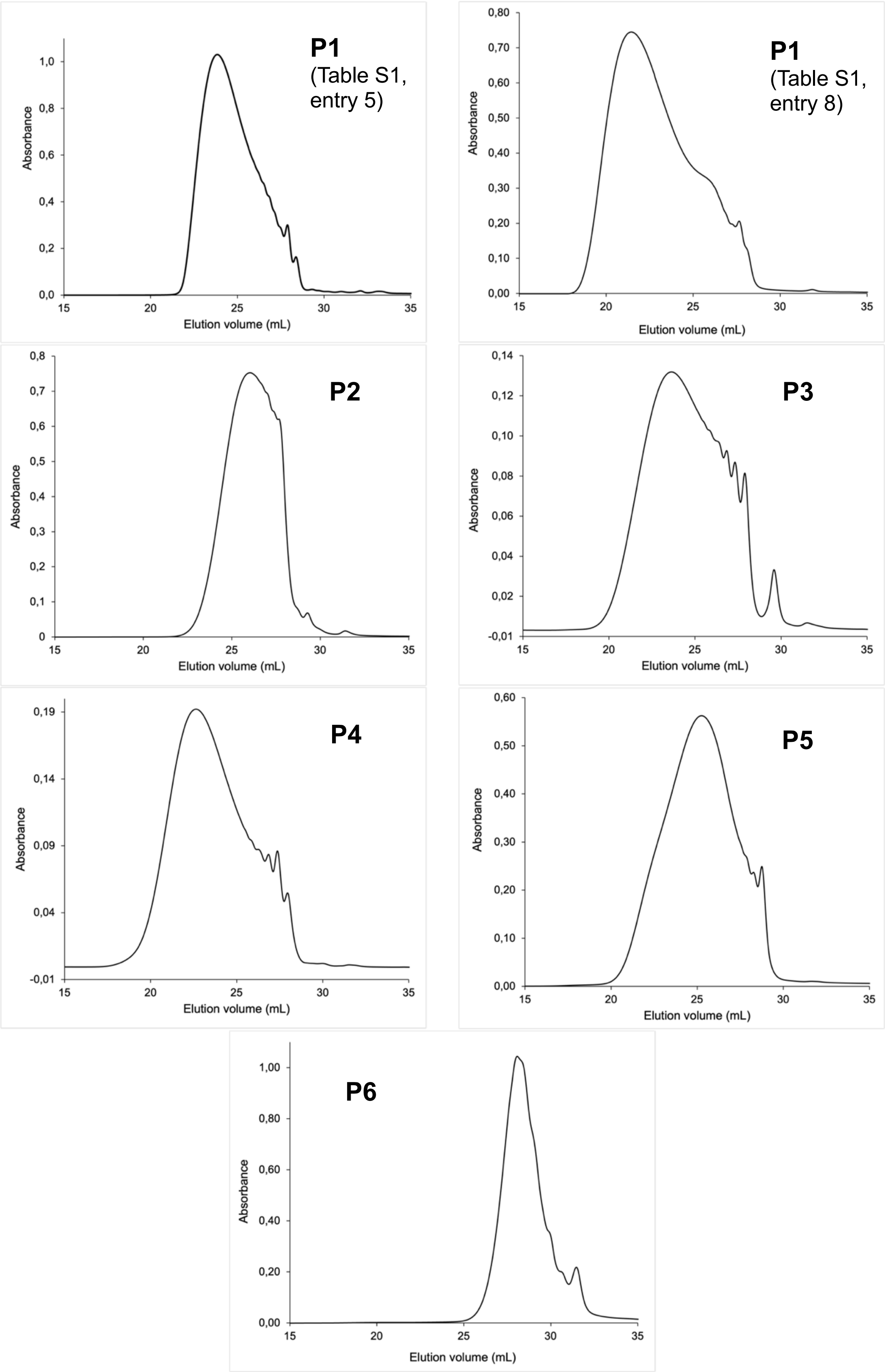

Figure S14. GPC traces of the isolated polymers.

6. NMR spectra of the non-previously reported compounds

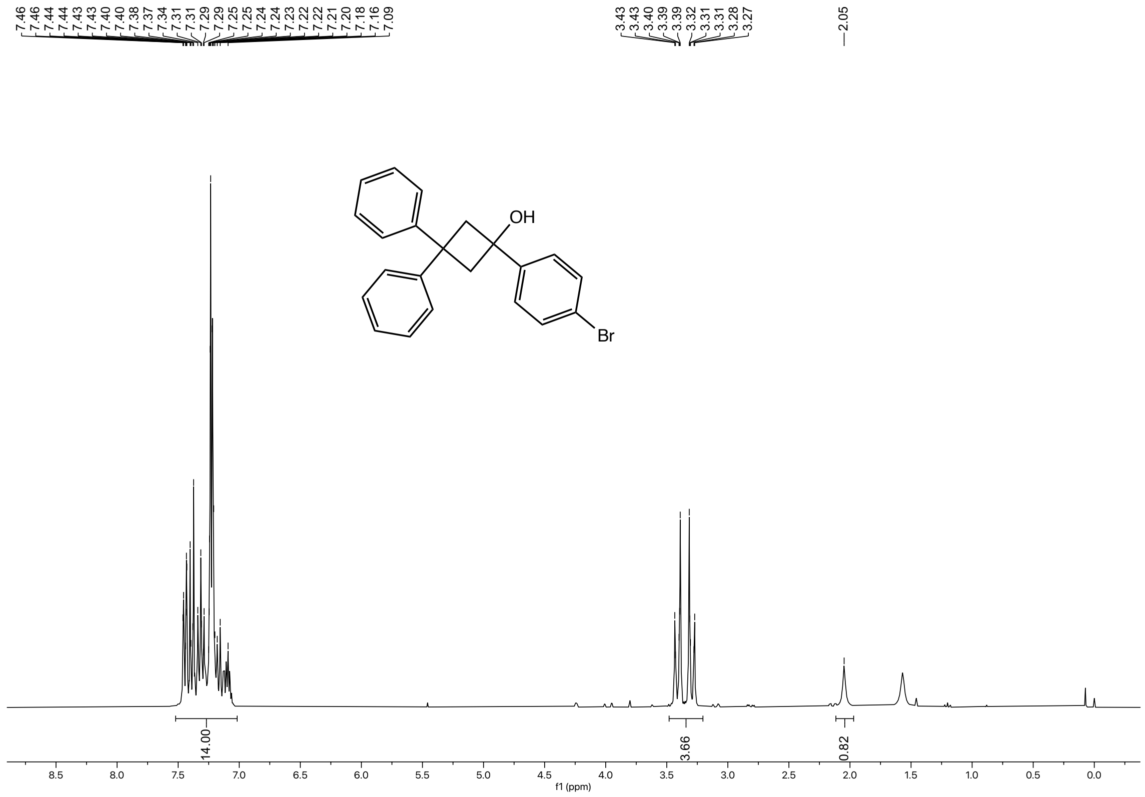

Figure S15. ^{1}H-NMR spectrum of **2** (300.1 MHz, $CDCl_3$).

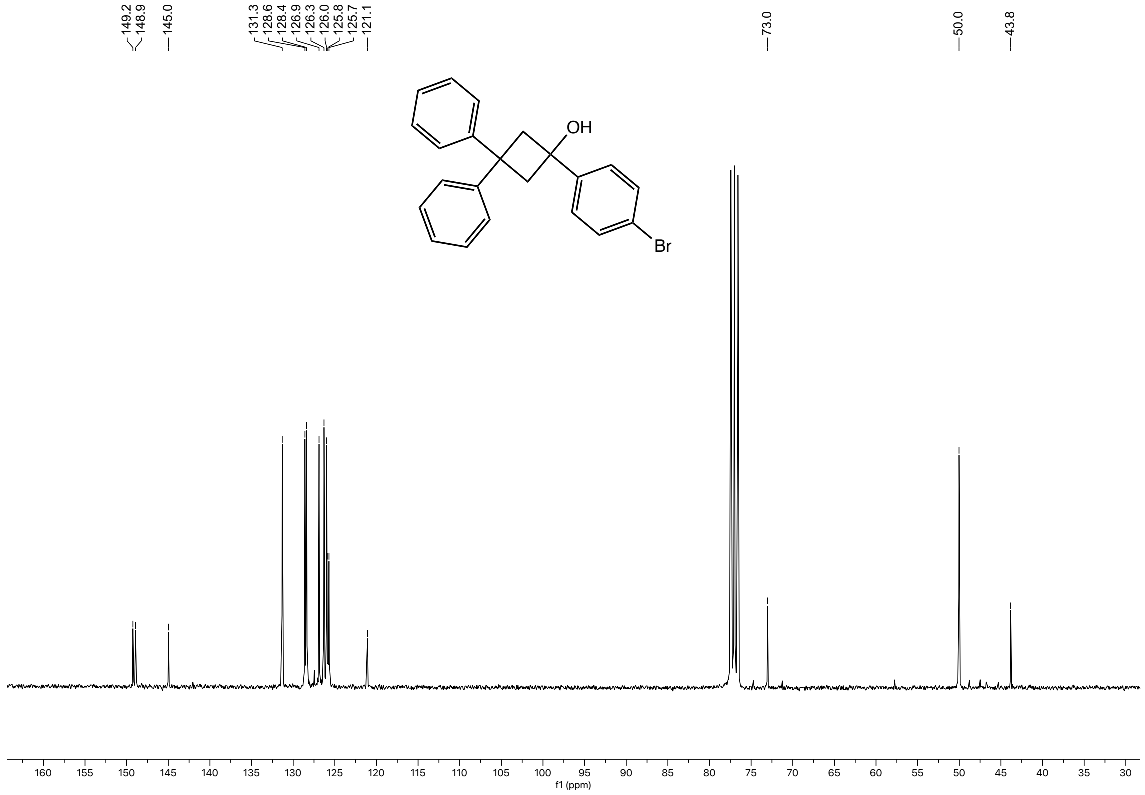

Figure S16. ^{13}C{^{1}H}-NMR spectrum of **2** (75.5 MHz, $CDCl_3$).

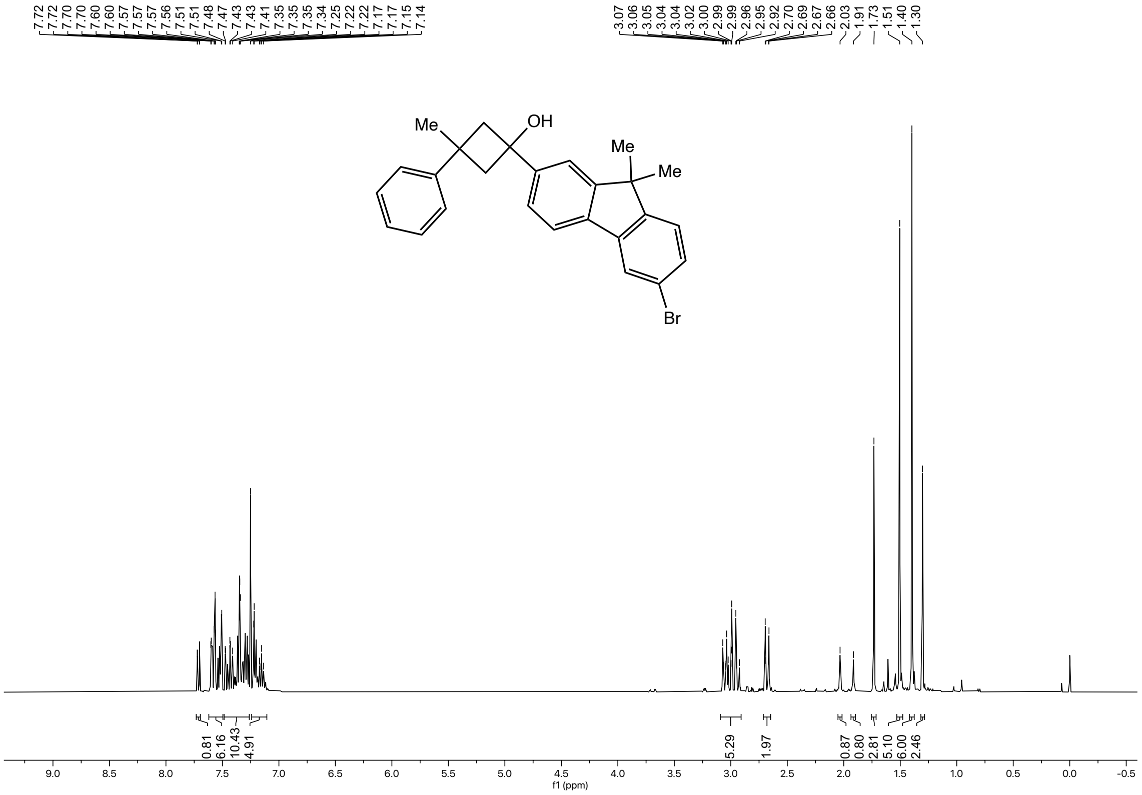

Figure S17. ^{1}H-NMR spectrum of **3** (ca. 1:0.9 mixture of diastereoisomers, 400.9 MHz, $CDCl_3$).

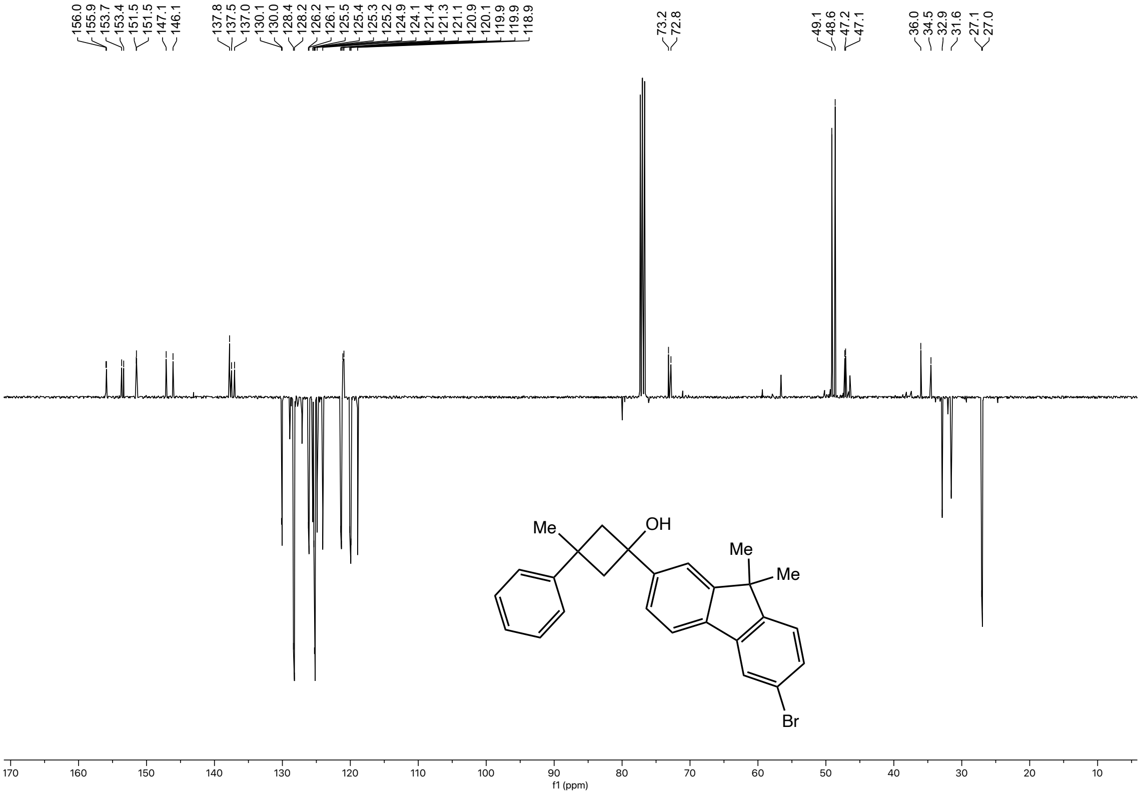

Figure S18. APT $^{13}C\{^{1}H\}$-NMR spectrum of **3** (ca. 1:0.9 mixture of diastereoisomers, 100.8 MHz, $CDCl_3$).

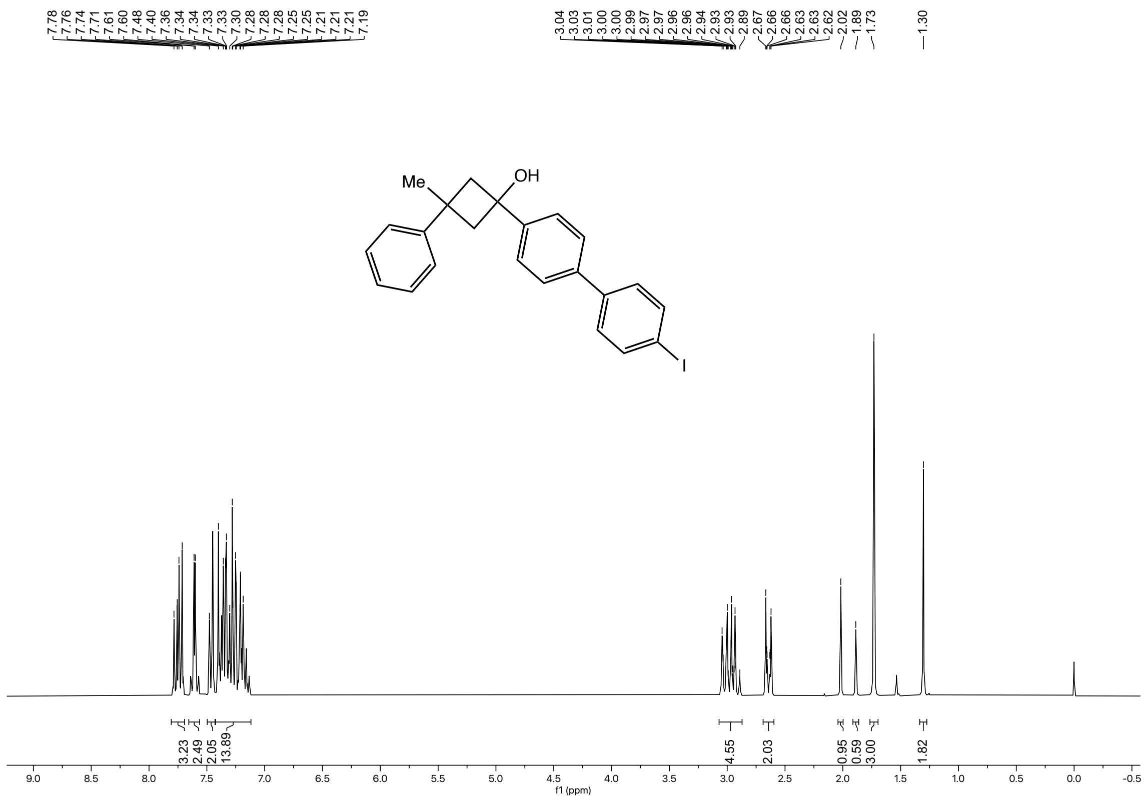

Figure S19. ^{1}H-NMR spectrum of **4** (ca. 1:0.6 mixture of diastereoisomers, 400.9 MHz, $CDCl_3$).

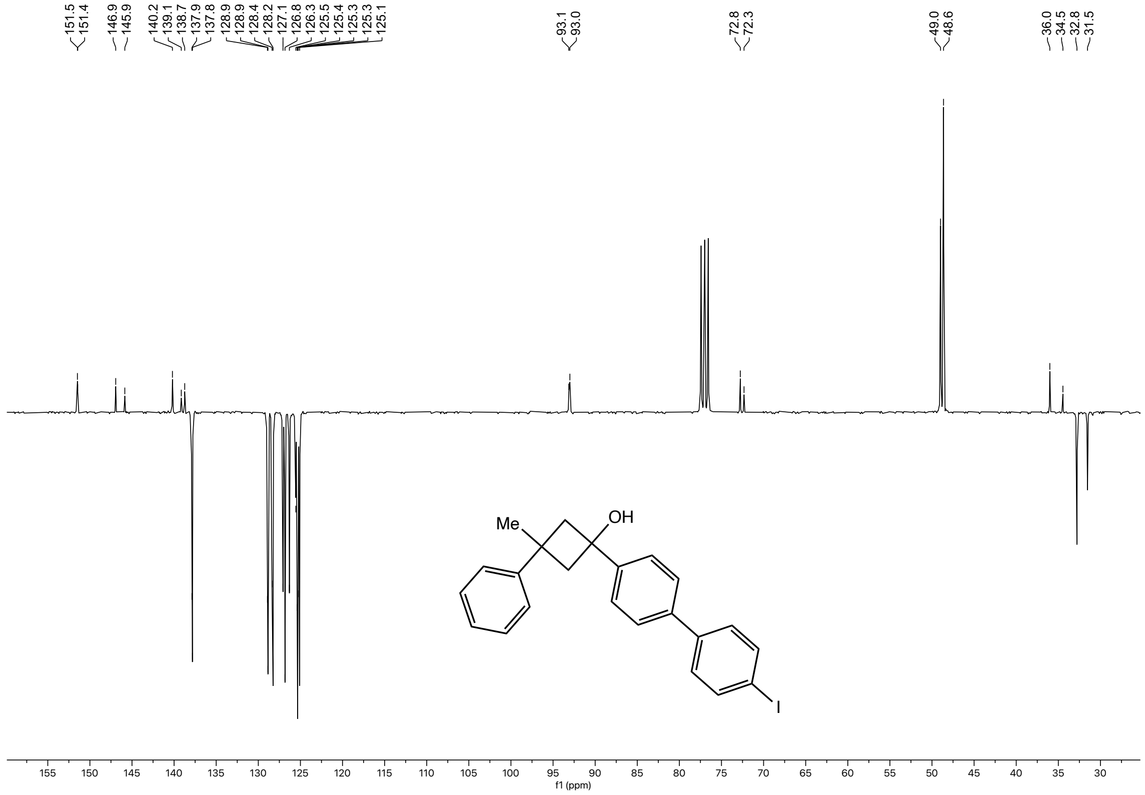

Figure S20. APT $^{13}C\{^1H\}$-NMR spectrum of **4** (ca. 1:0.6 approx. mixture of diastereoisomers, 75.5 MHz, $CDCl_3$).

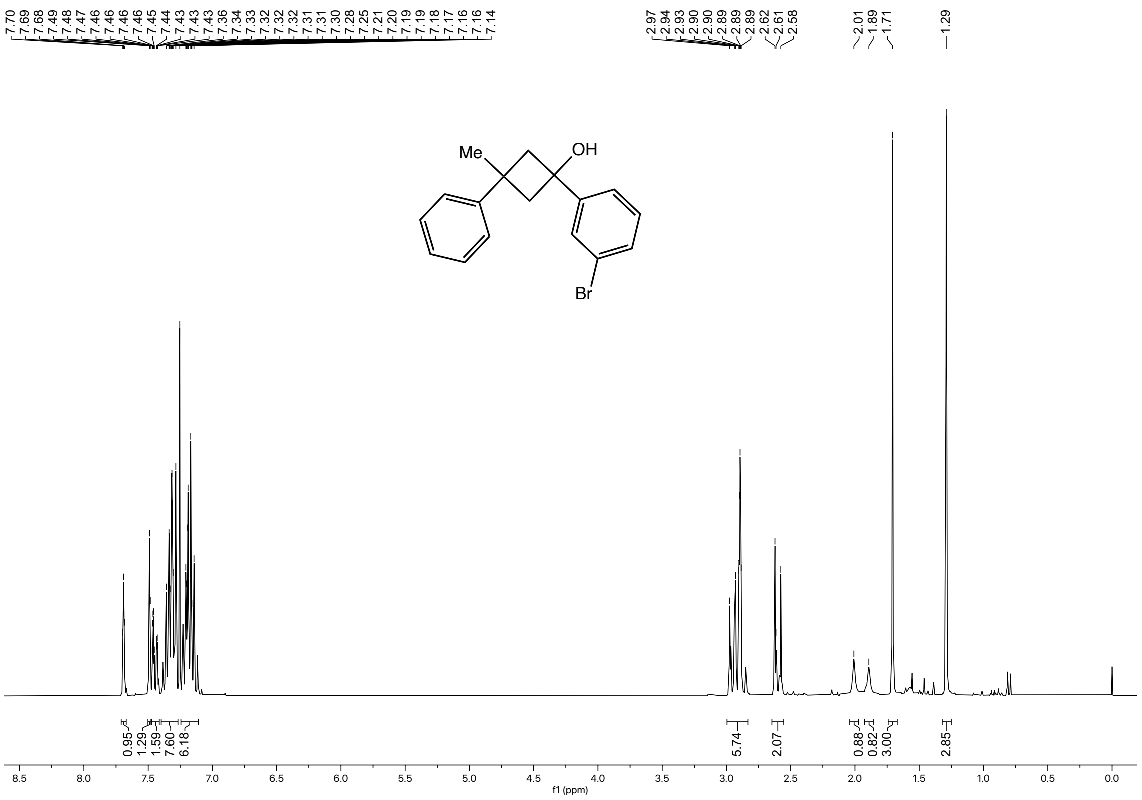

Figure S21. ^{1}H-NMR spectrum of **5** (ca. 1:1 approx. mixture of diastereoisomers, 300.1 MHz, $CDCl_3$).

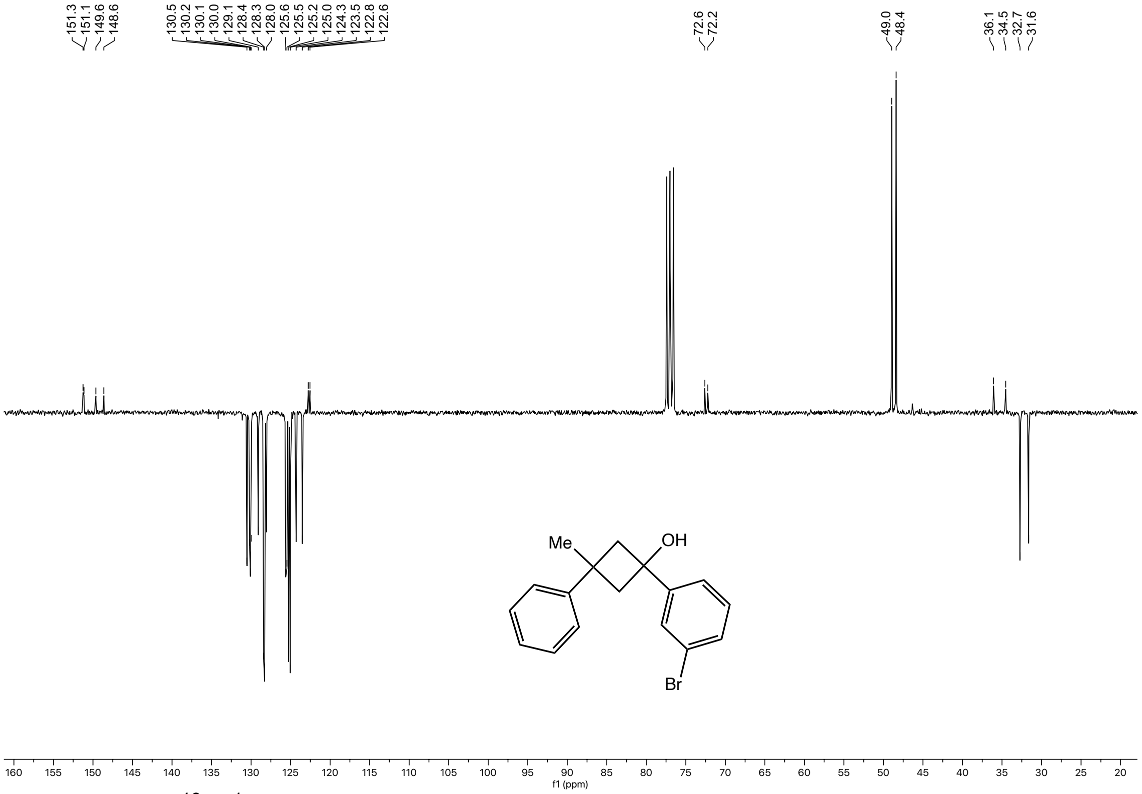

Figure S22. APT $^{13}C\{^1H\}$-NMR spectrum of **5** (ca. 1:1 approx. mixture of diastereoisomers, 75.5 MHz, $CDCl_3$).

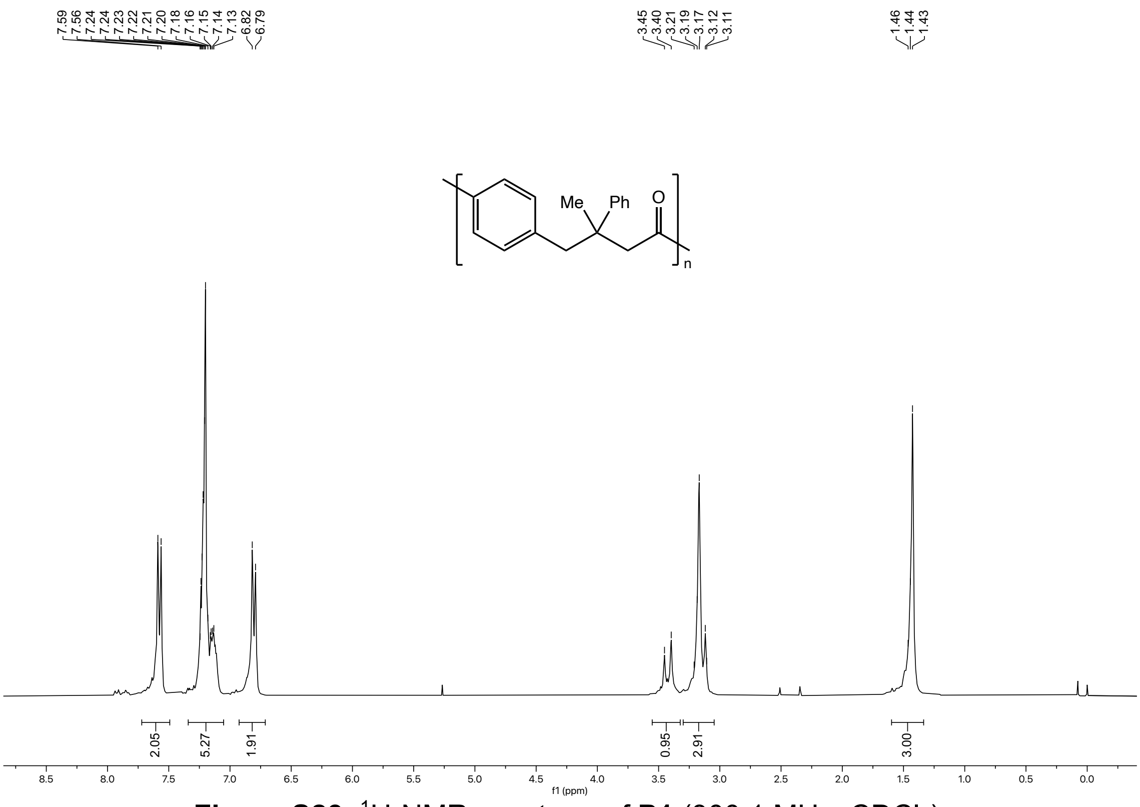

Figure S23. ^{1}H-NMR spectrum of **P1** (300.1 MHz, $CDCl_3$).

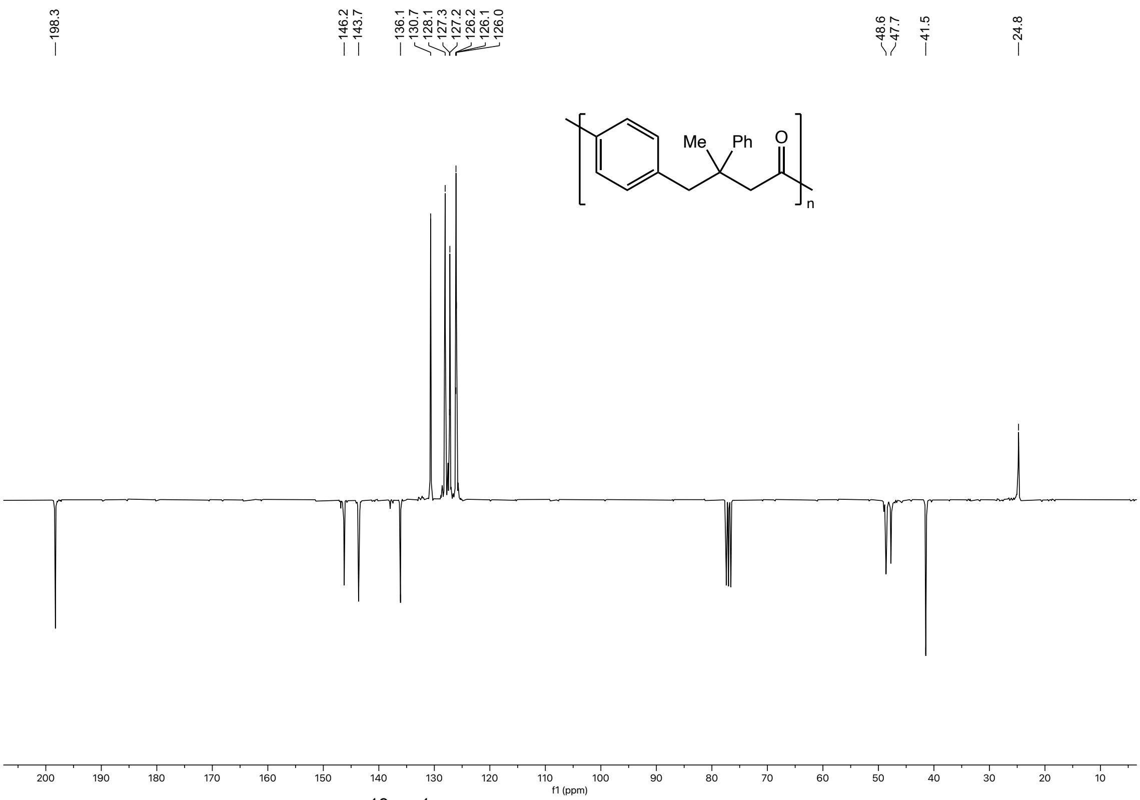

Figure S24. APT $^{13}C\{^{1}H\}$-NMR spectrum of **P1** (75.5 MHz, $CDCl_3$).

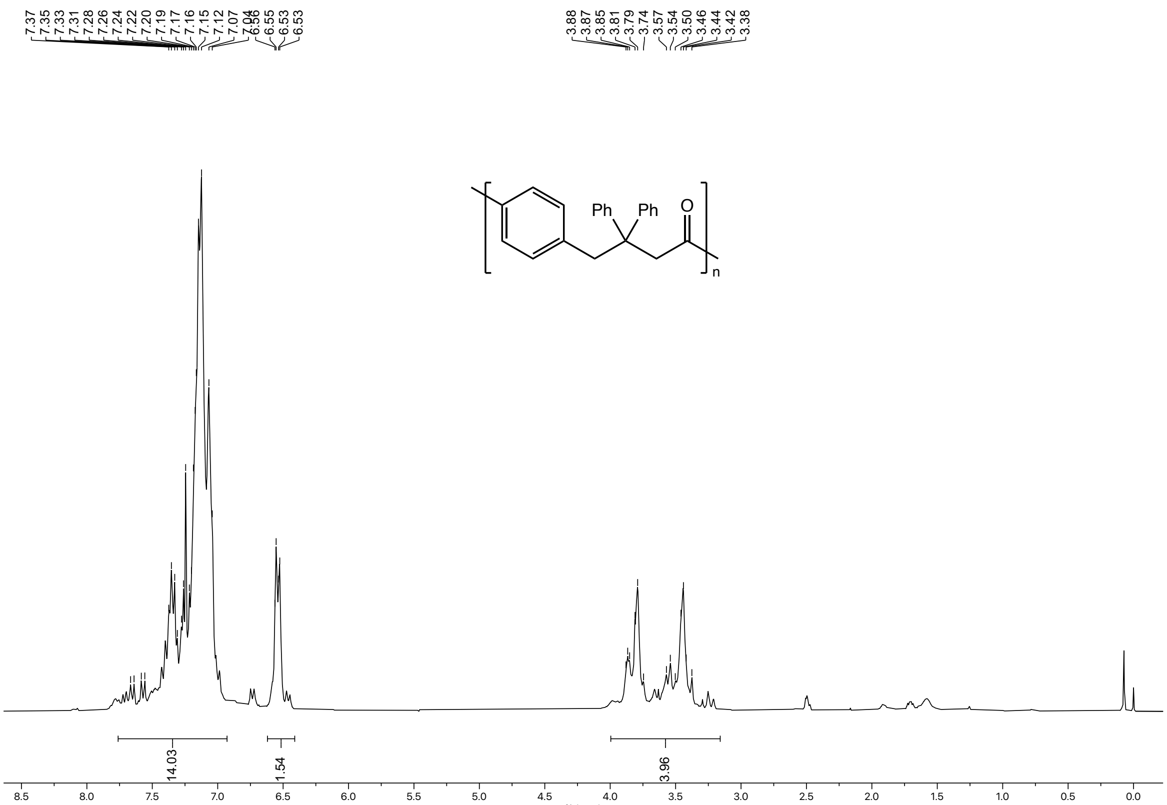

Figure S25. ^{1}H-NMR spectrum of **P2** (300.1 MHz, $CDCl_3$).

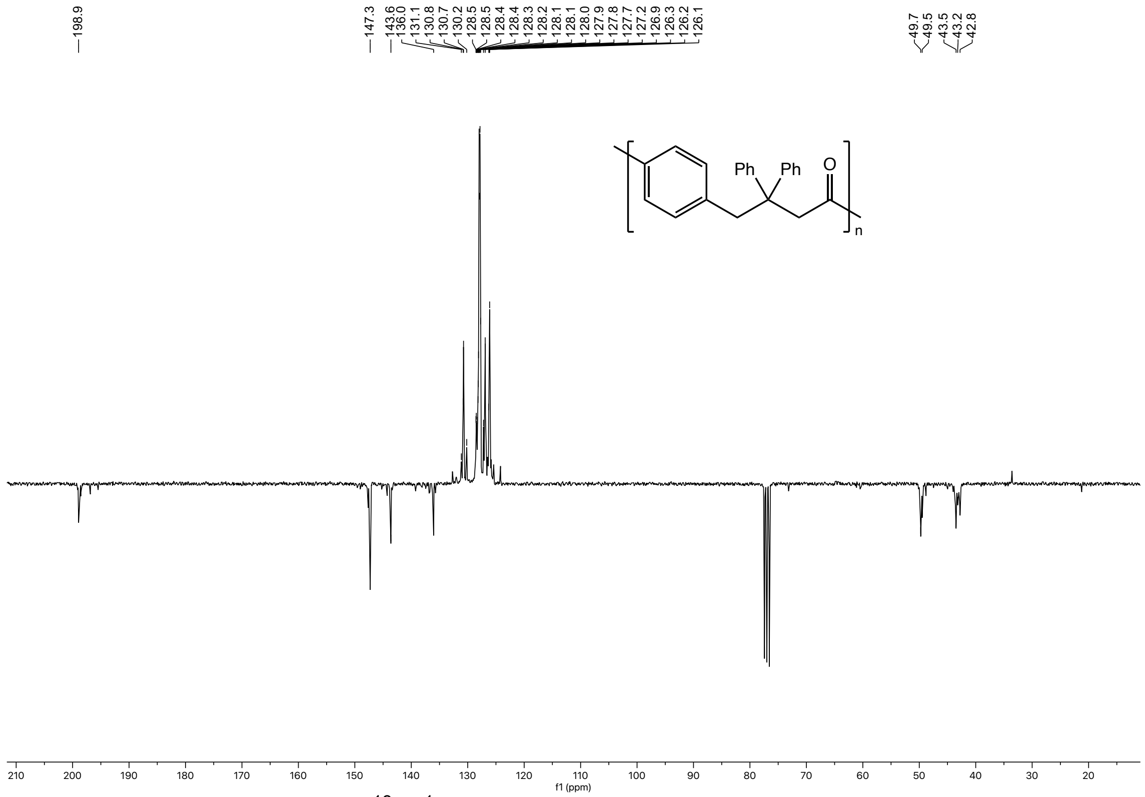

Figure S26. APT $^{13}C\{^{1}H\}$-NMR spectrum of **P2** (75.5 MHz, $CDCl_3$).

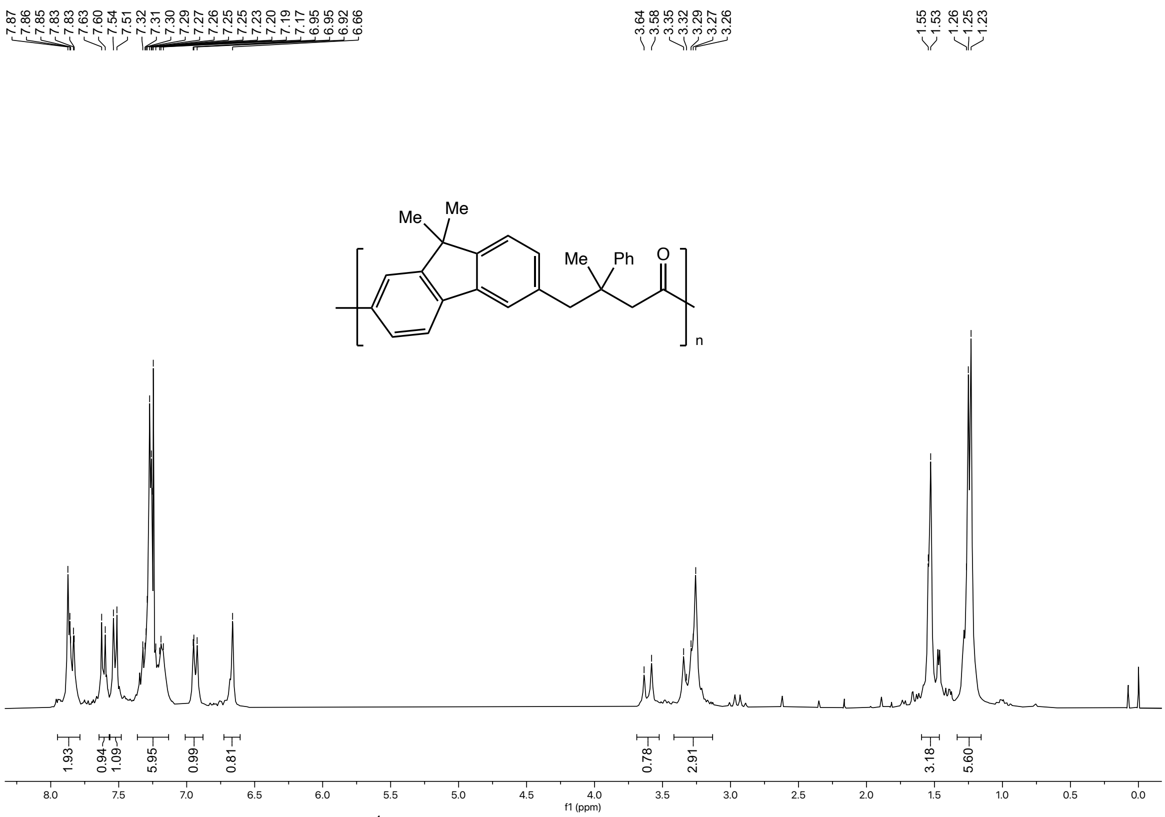

Figure S27. ^{1}H-NMR spectrum of **P3** (300.1 MHz, $CDCl_3$).

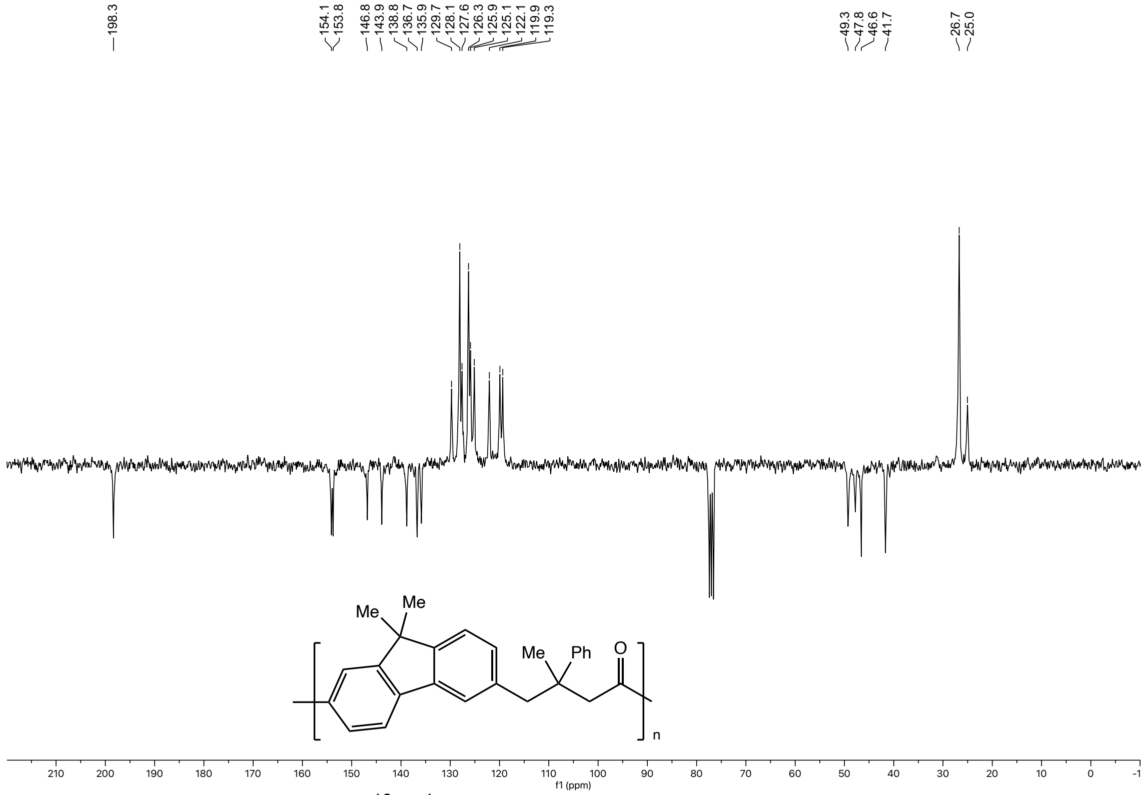

Figure S28. APT $^{13}C\{^{1}H\}$-NMR spectrum of **P3** (75.5 MHz, $CDCl_3$).

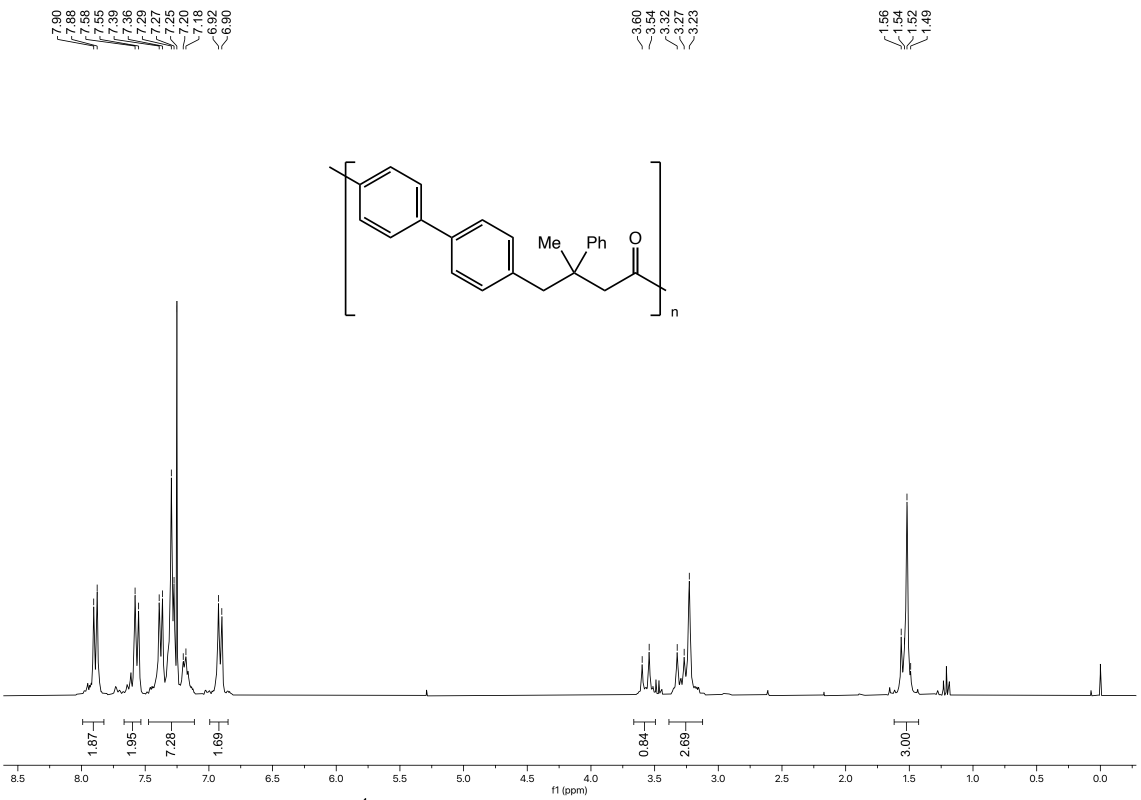

Figure S29. ^{1}H-NMR spectrum of **P4** (300.1 MHz, $CDCl_3$).

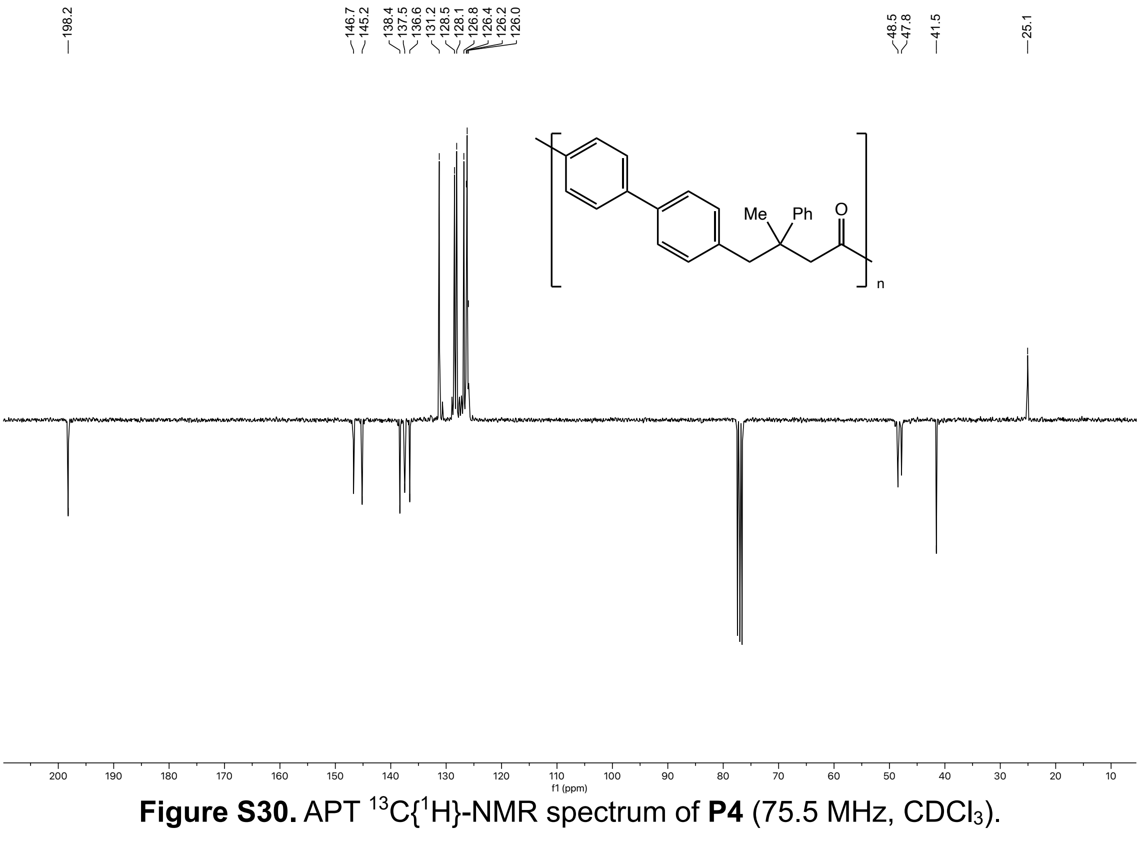

Figure S30. APT $^{13}C\{^1H\}$-NMR spectrum of **P4** (75.5 MHz, $CDCl_3$).

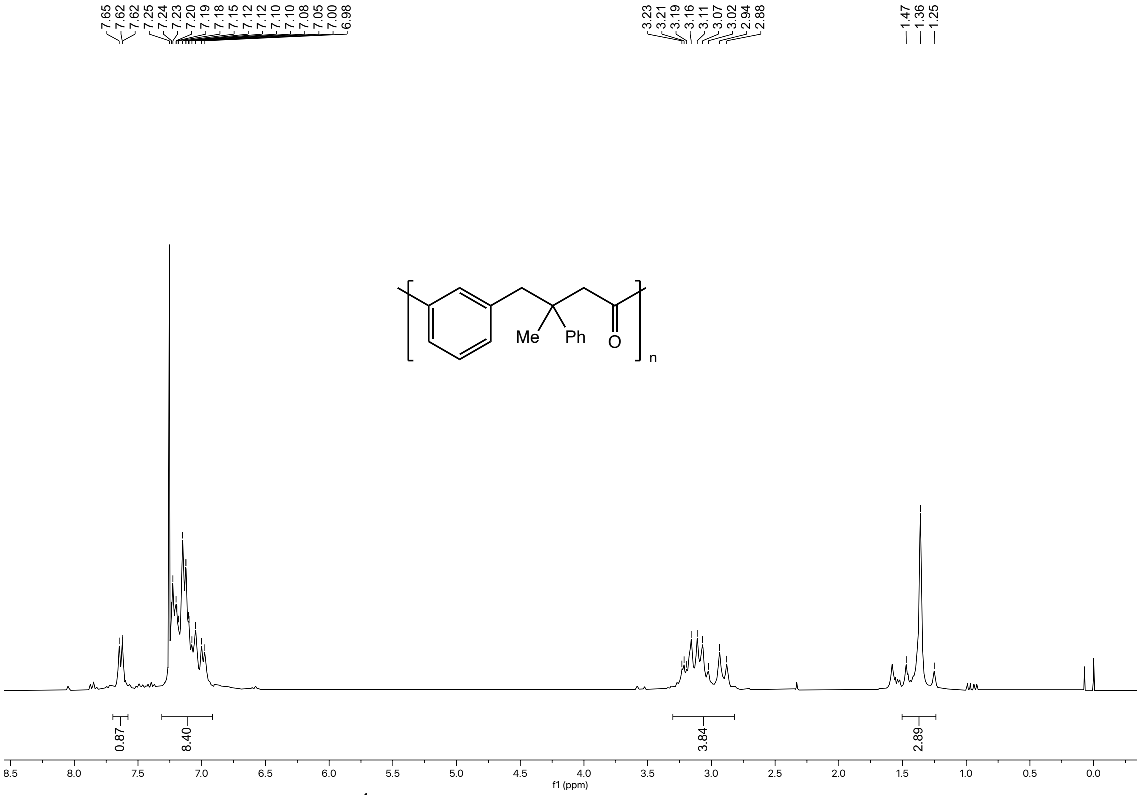

Figure S31. ^{1}H-NMR spectrum of **P5** (300.1 MHz, $CDCl_3$).

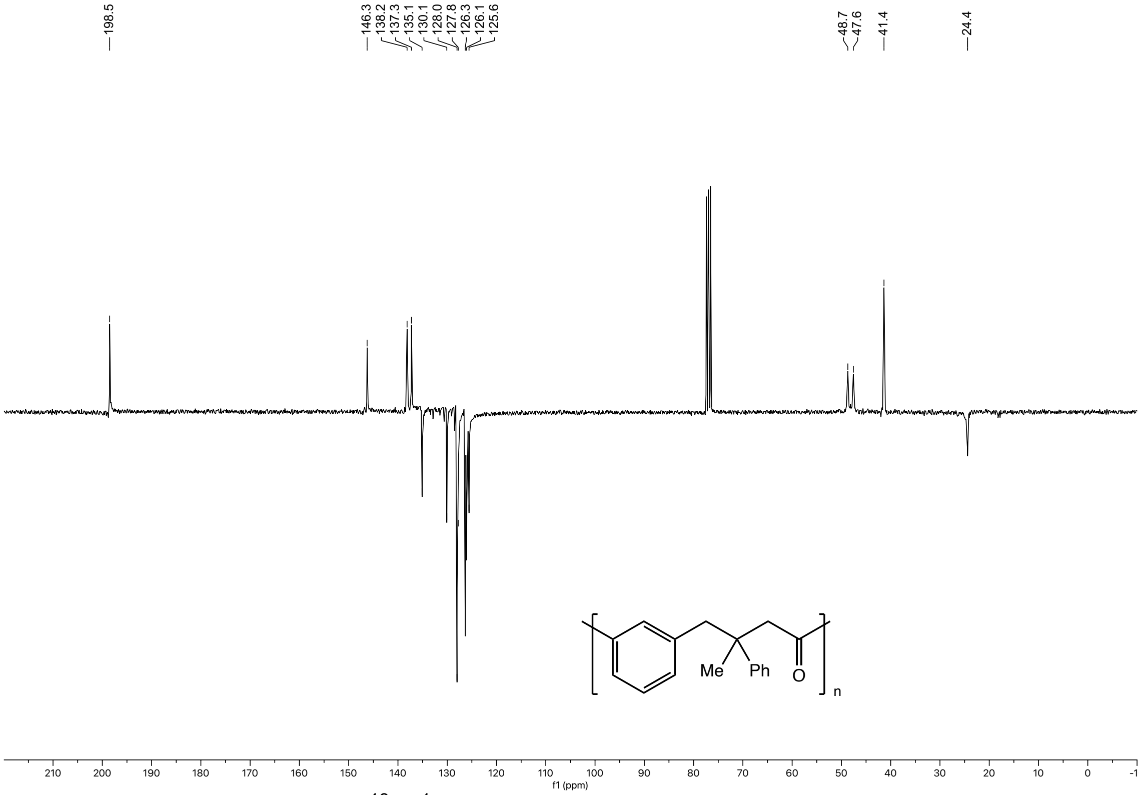

Figure S32. APT $^{13}C\{^{1}H\}$-NMR spectrum of **P5** (75.5 MHz, $CDCl_3$).

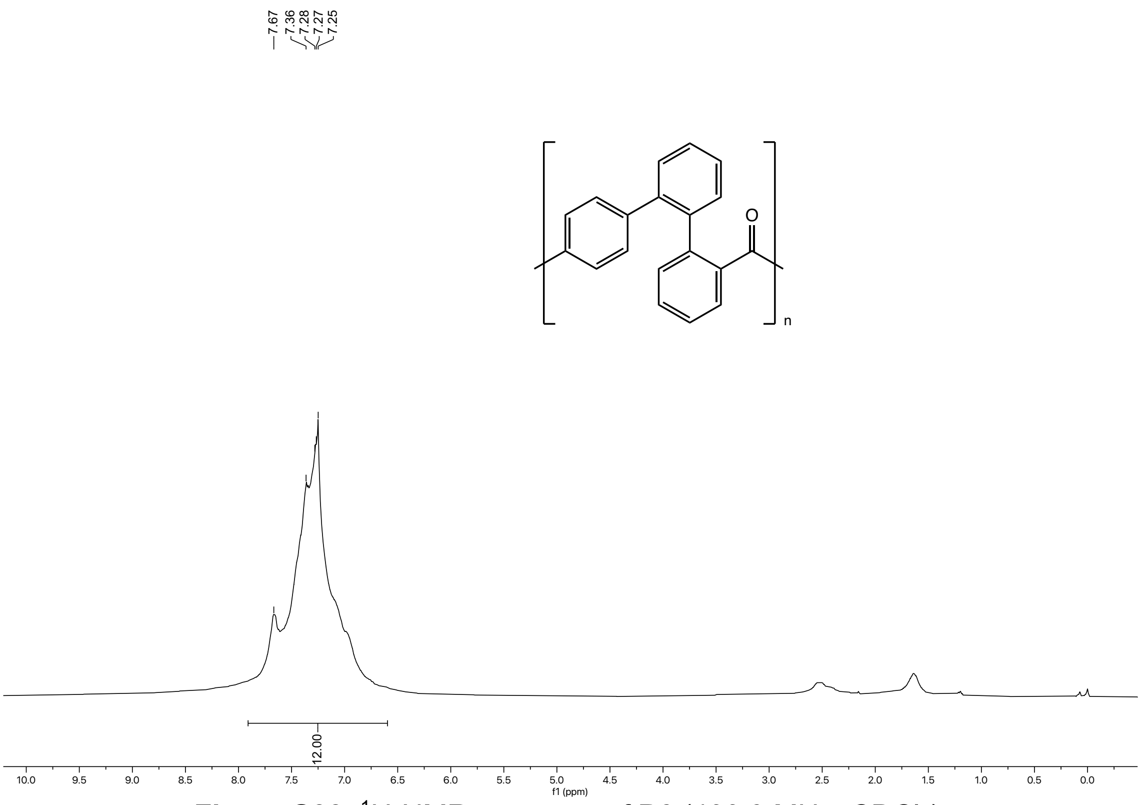

Figure S33. ^{1}H-NMR spectrum of **P6** (400.9 MHz, $CDCl_3$).

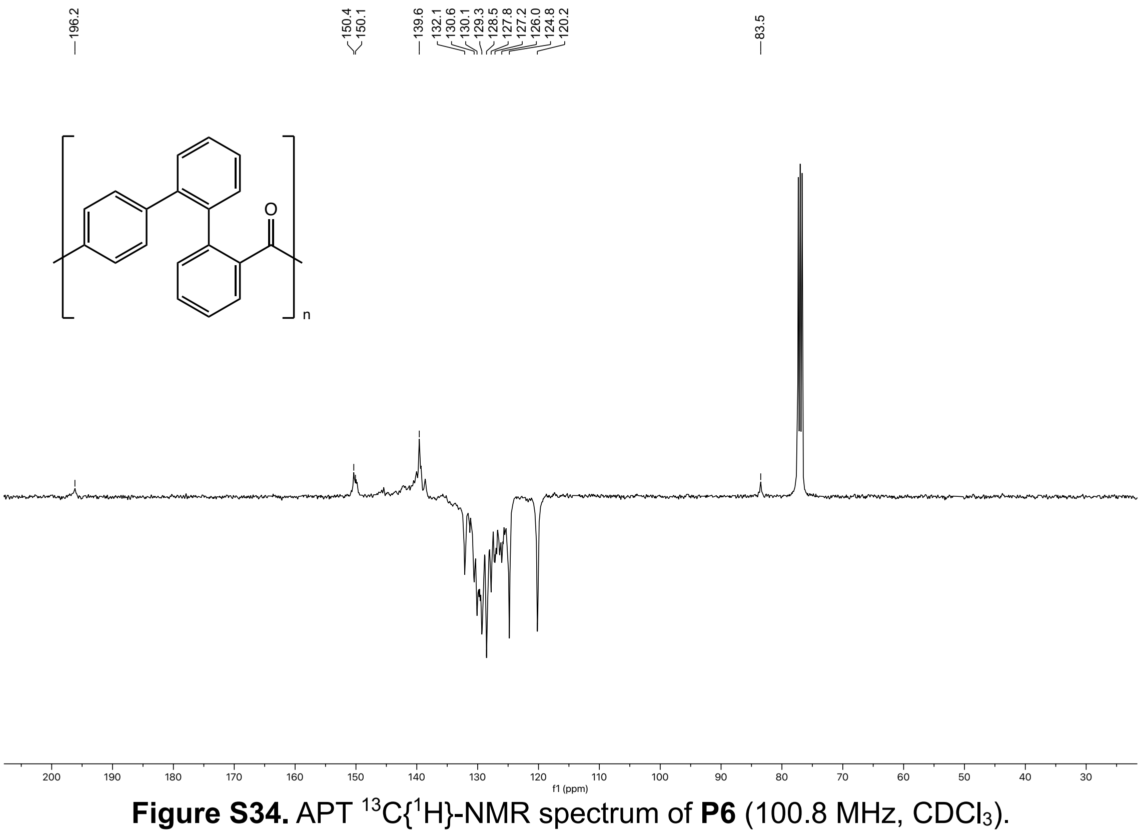

Figure S34. APT $^{13}C\{^{1}H\}$-NMR spectrum of **P6** (100.8 MHz, $CDCl_3$).

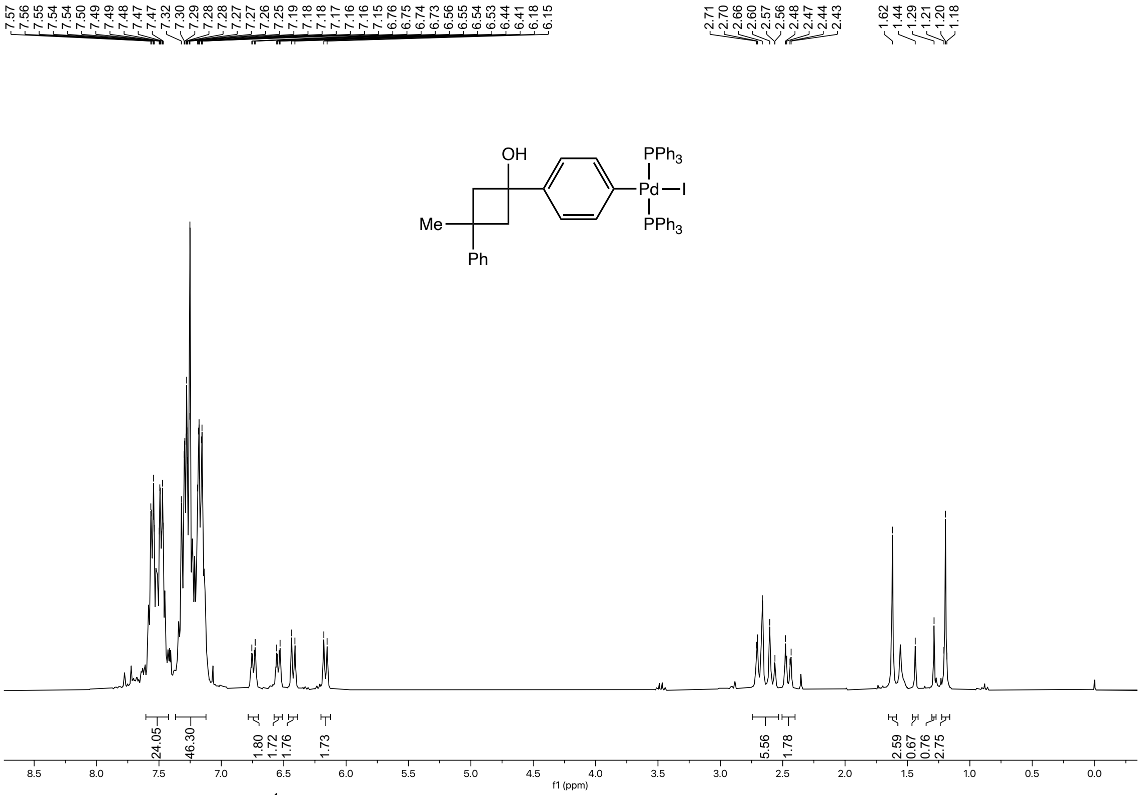

Figure S35. ^{1}H-NMR spectrum of crude complex **7** (300.1 MHz, $CDCl_3$).

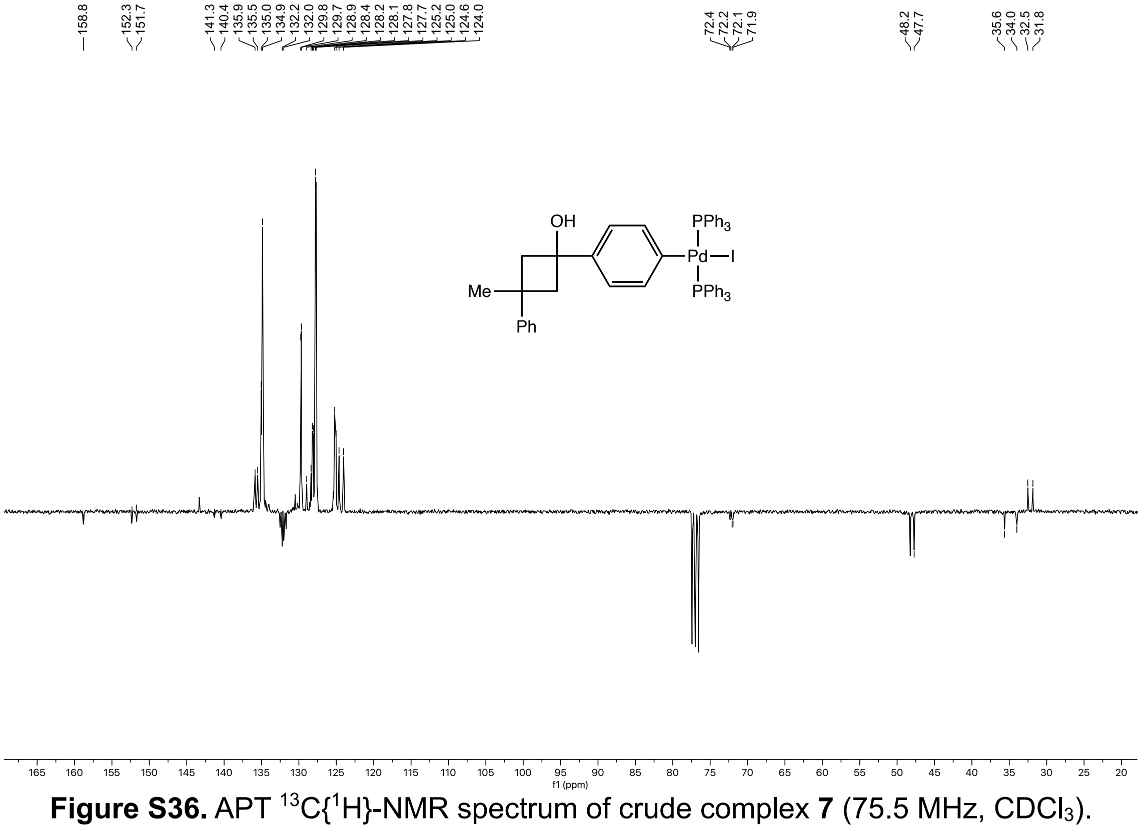

Figure S36. APT $^{13}C\{^1H\}$-NMR spectrum of crude complex **7** (75.5 MHz, $CDCl_3$).

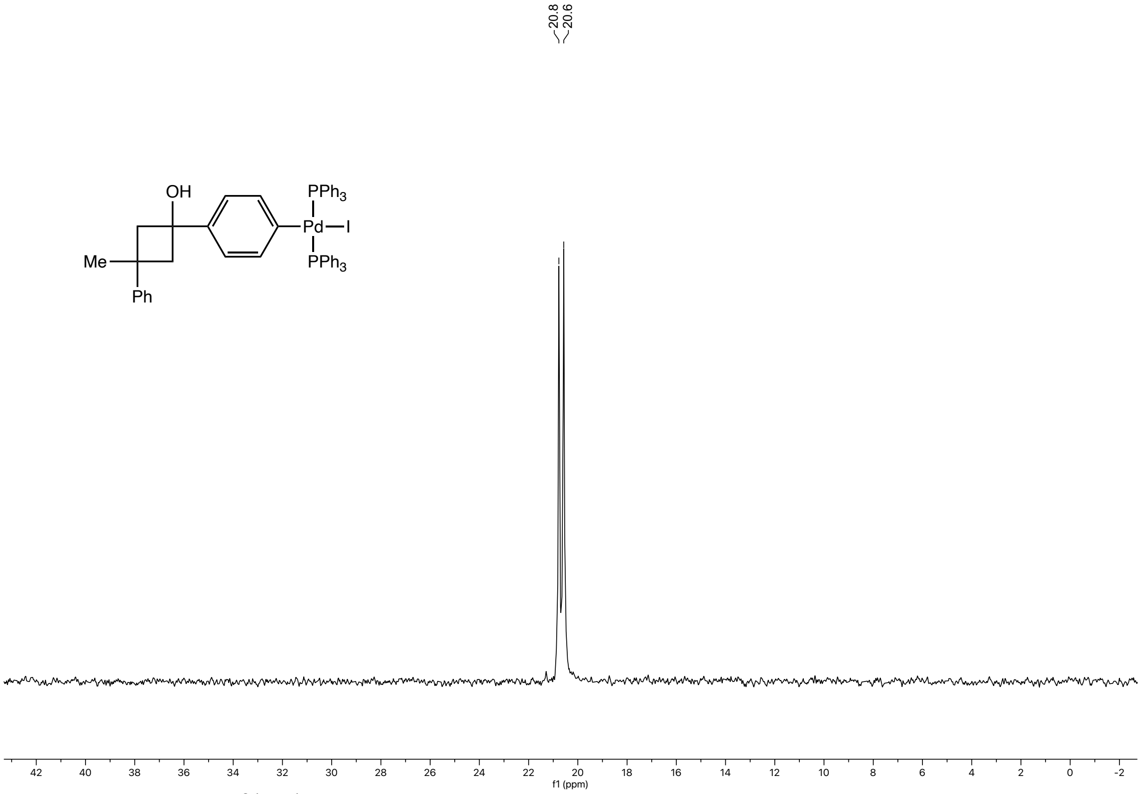

Figure S37. $^{31}P\{^{1}H\}$-NMR spectrum of crude complex **7** (121.5 MHz, $CDCl_3$).

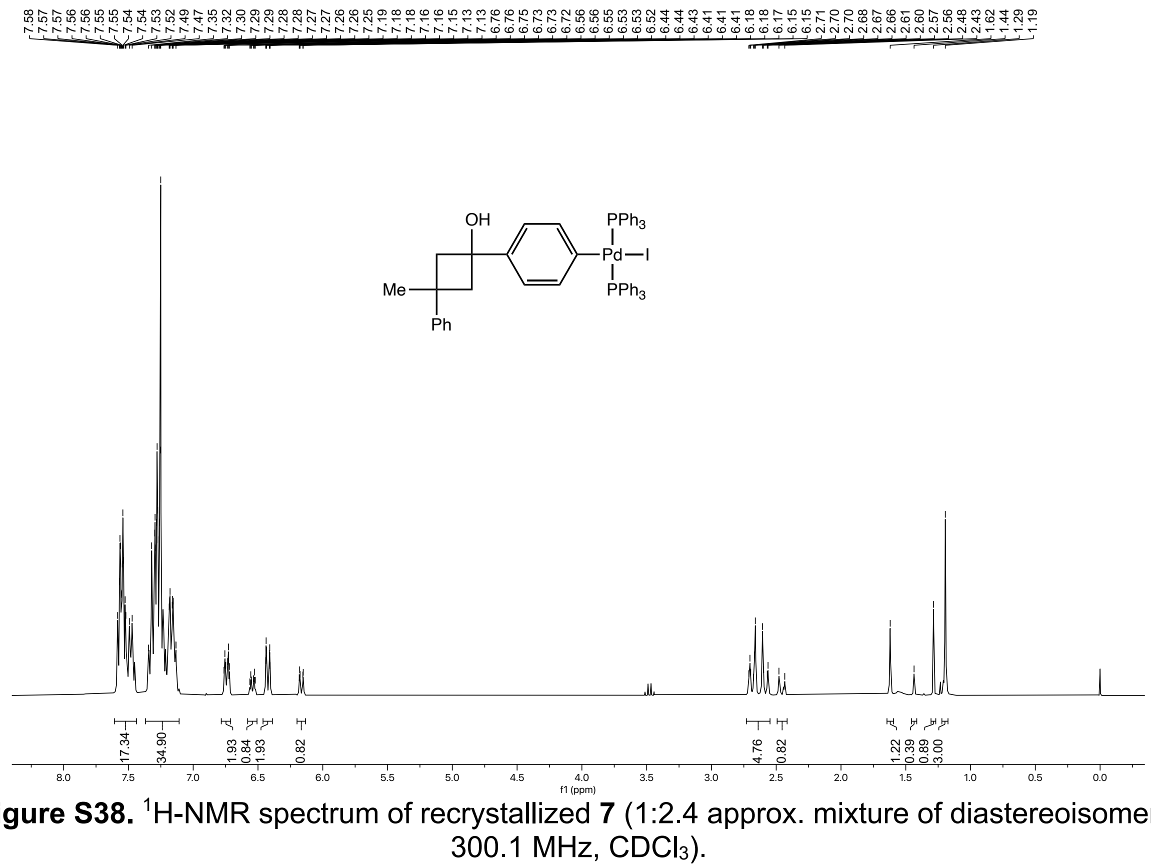

Figure S38. ^{1}H-NMR spectrum of recrystallized **7** (1:2.4 approx. mixture of diastereoisomers, 300.1 MHz, $CDCl_3$).

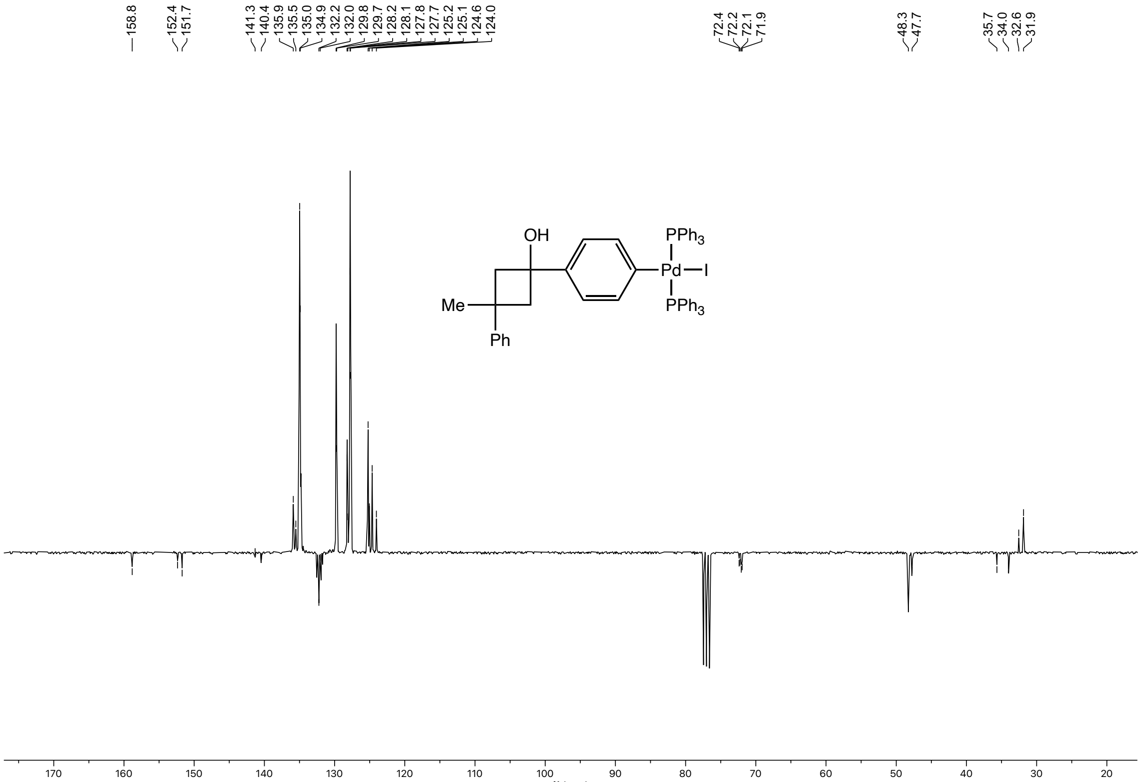

Figure S39. APT $^{13}C\{^1H\}$-NMR spectrum of recrystallized **7** (1:2.4 approx. mixture of diastereoisomers, 75.5 MHz, $CDCl_3$).

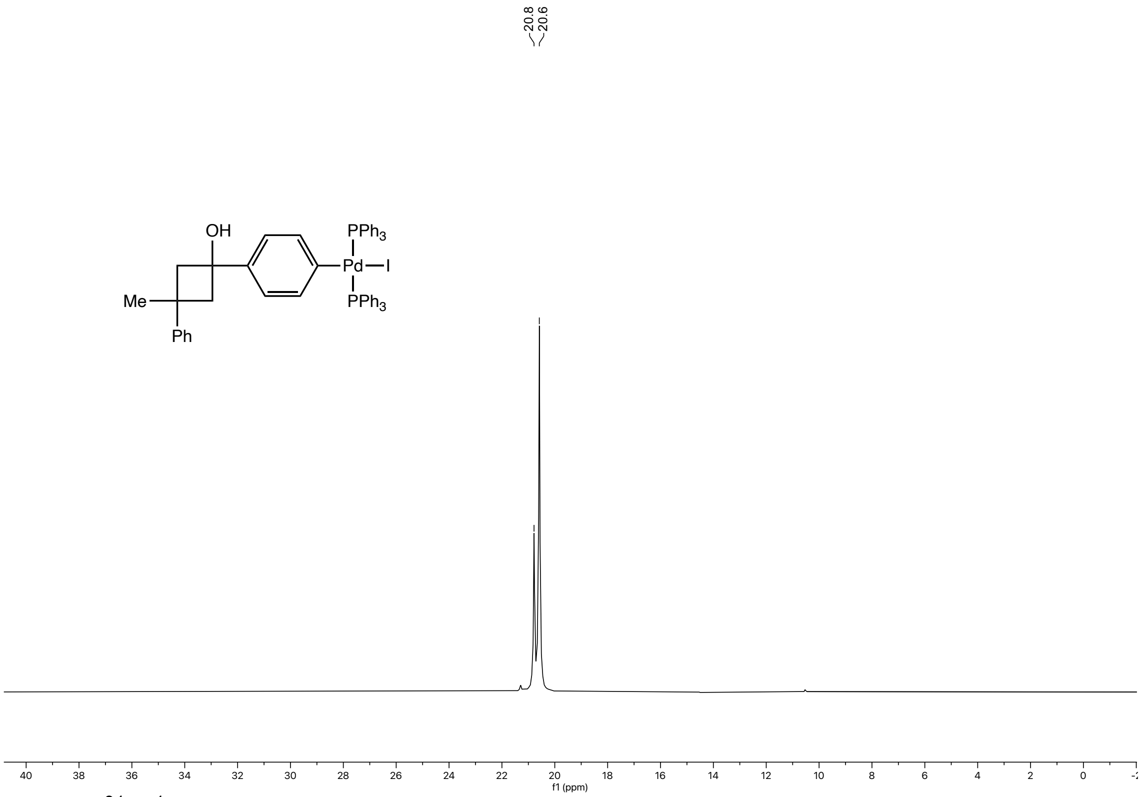

Figure S40. $^{31}P\{^1H\}$-NMR spectrum of recrystallized **7** (1:2.4 approx. mixture of diastereoisomers, 121.5 MHz, $CDCl_3$).

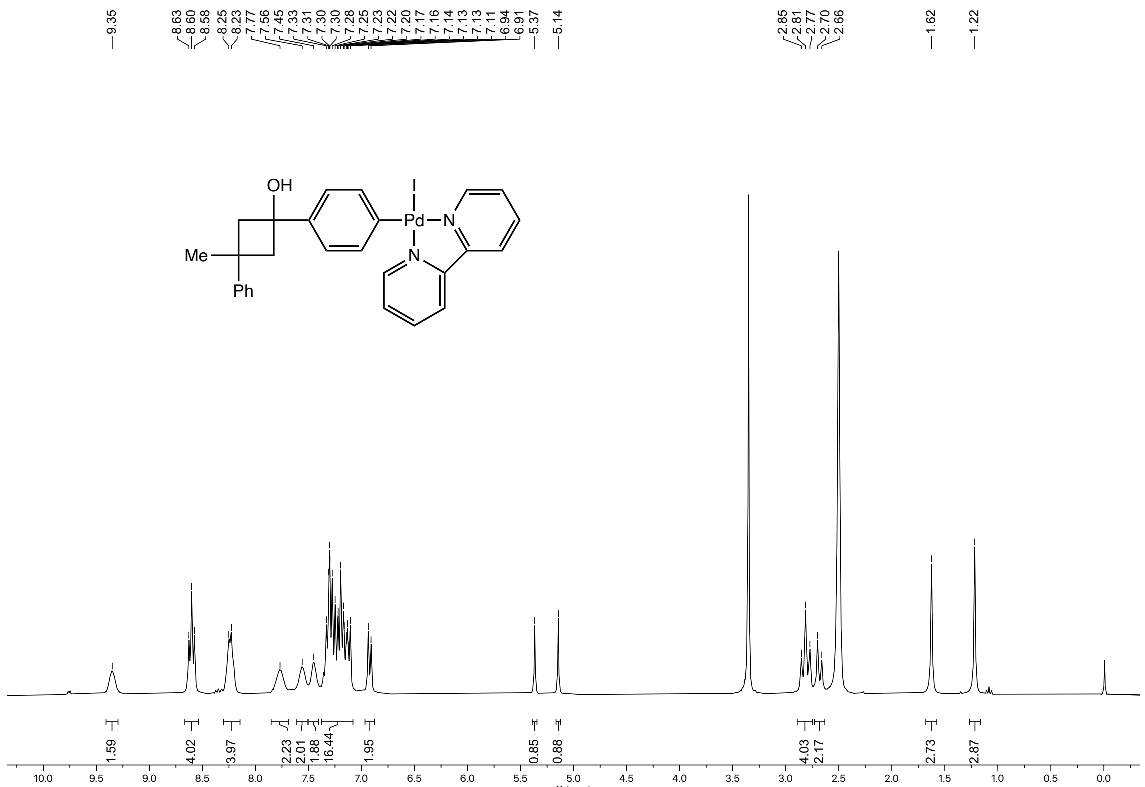

Figure S41. ^{1}H-NMR spectrum of a ca. 1:1 mixture of diastereoisomers of compound **8** (300.1 MHz, DMSO-d_6)

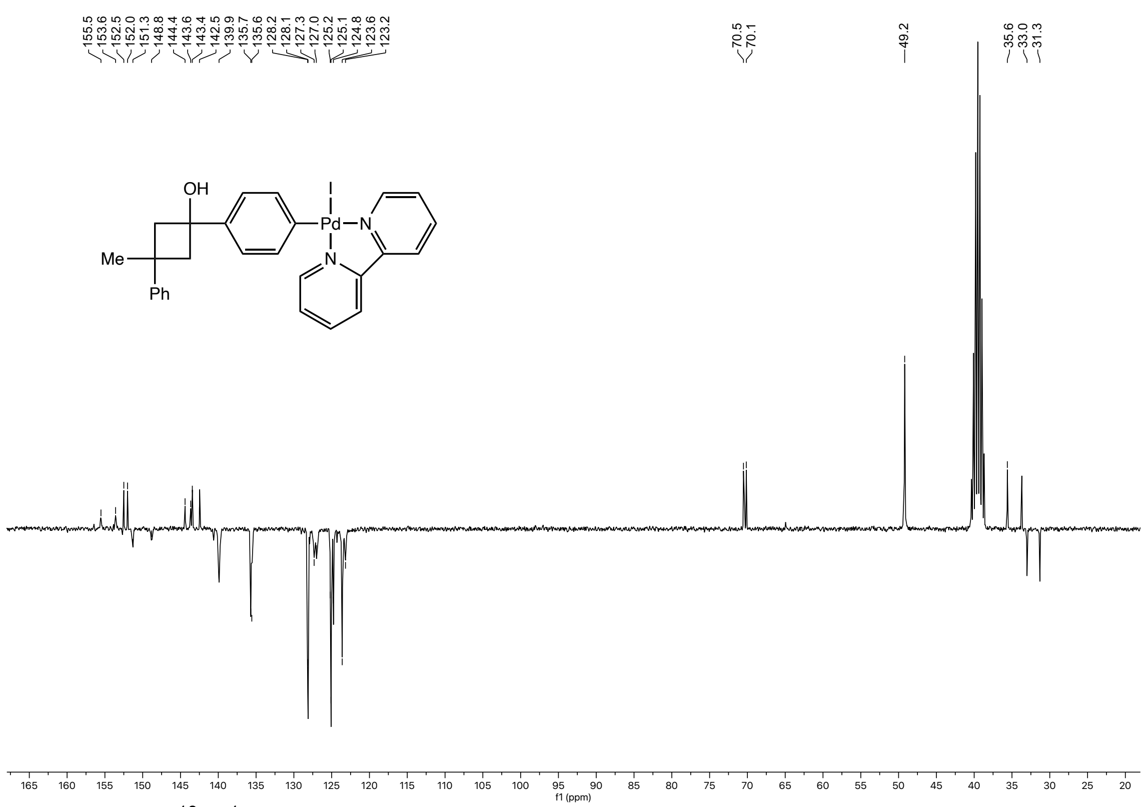

Figure S42. APT $^{13}C\{^{1}H\}$-NMR spectrum of a ca. 1:1 mixture of diastereoisomers of compound **8** (75.5 MHz, DMSO-d_6).

7. TGA and DSC traces of the polymers

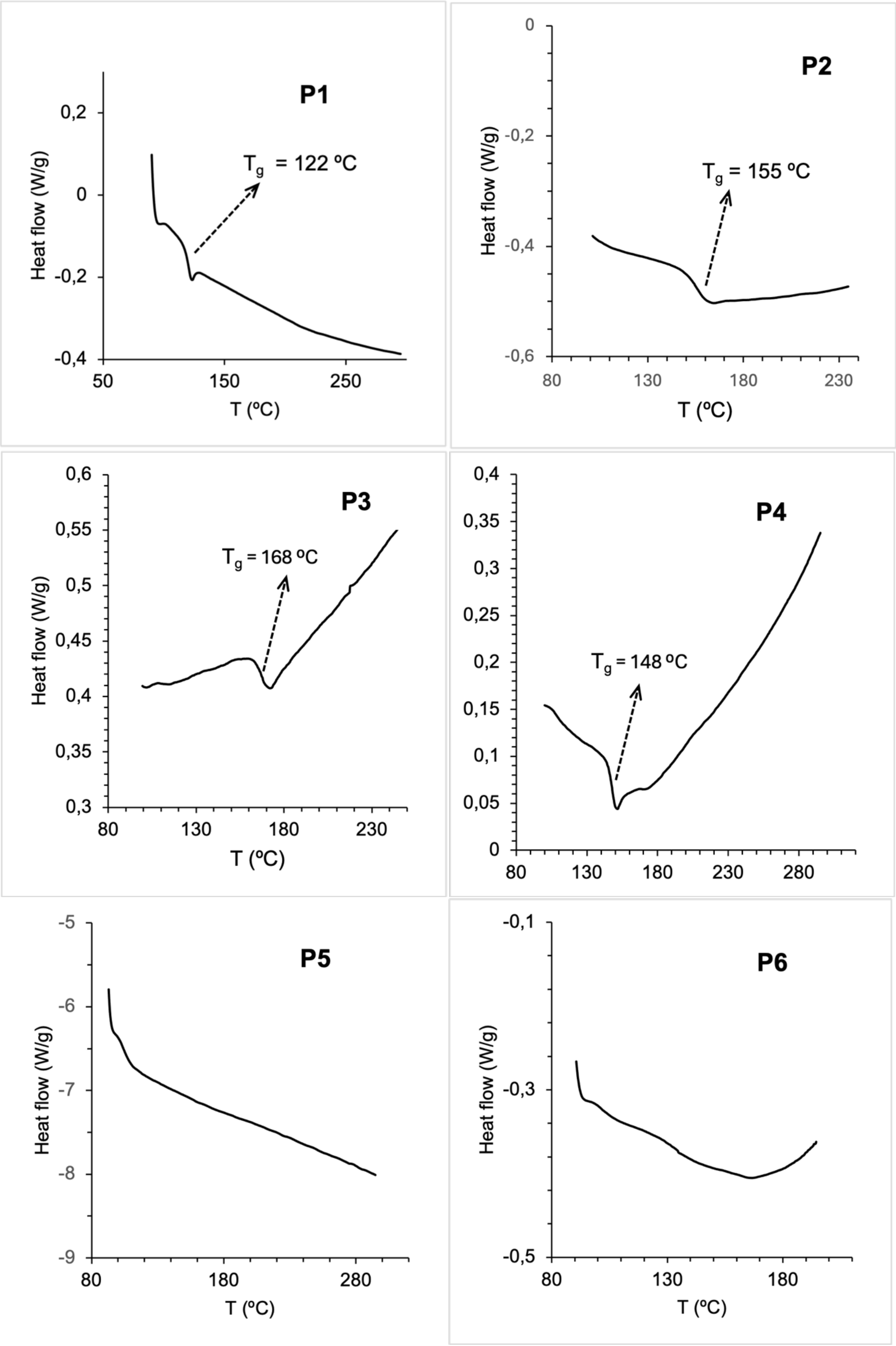

Figure S43. DSC traces of the polymers. Exothermic peaks point upward.

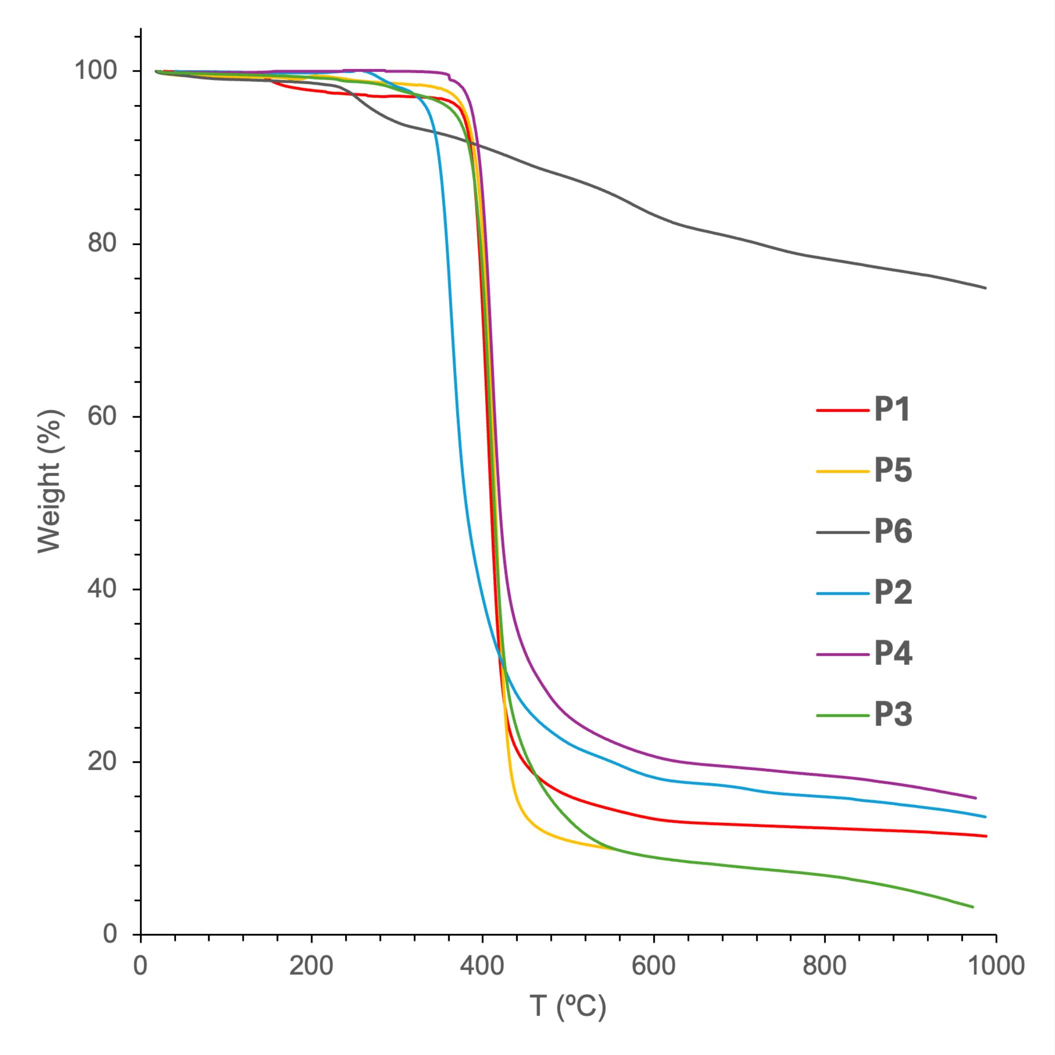

Figure S44. TGA traces of the polymers.

8. References

[1] Seiser, T.; Roth, O.A.; Cramer, N. *Angew. Chem. Int. Ed.* **2009**, *48*, 6320–6323.

[2] Parra-García, S.; Ballester-Ibáñez, M.; García-López, J.-A. *J. Org. Chem.* **2024**, *89*, 882–886.

[3] Hsieh, W.-C.; Shaikh, A.Y.; Perera, D.R.; Thadke, S.A.; Ly, D.H. *J. Org. Chem.* **2019**, *84*, 1276-1287.

[4] a) Grushin, V. V. *Organometallics* **2000**, *19*, 1888–1900. Lee, Y. H.; b) Wang, L.; Chen, H.; Duan, Z. *Chem. Asian J.* **2018**, *13*, 2164–217; c) Morandi, B. *Coord. Chem. Rev.* **2019**, *386*, 96–118.

[5] (a) Sheldrick, G. M. Crystal structure refinement with SHELXL. *Acta Crystallogr., Sect. C: Struct. Chem.* **2015**, *71*, 3−8. (b) Sheldrick, G. M. SHELXL-2018/3, Program for the Refinement of Crystal Structure; University of Göttingen: Göttingen, Germany, 2018.
